# Measuring anion binding at biomembrane interfaces

**DOI:** 10.1038/s41467-022-32403-z

**Published:** 2022-08-08

**Authors:** Xin Wu, Patrick Wang, William Lewis, Yun-Bao Jiang, Philip A. Gale

**Affiliations:** 1 School of Chemistry, The University of Sydney, Sydney, NSW 2006 Australia; 2 Department of Chemistry, College of Chemistry and Chemical Engineering, The MOE Key Laboratory of Spectrochemical Analysis and Instrumentation, and iChEM, Xiamen University, 361005 Xiamen, China

**Keywords:** Supramolecular chemistry, Coordination chemistry

## Abstract

The quantification of anion binding by molecular receptors within lipid bilayers remains challenging. Here we measure anion binding in lipid bilayers by creating a fluorescent macrocycle featuring a strong sulfate affinity. We find the determinants of anion binding in lipid bilayers to be different from those expected that govern anion binding in solution. Charge-dense anions H_2_PO_4_^–^ and Cl^–^ that prevail in dimethyl sulfoxide fail to bind to the macrocycle in lipids. In stark contrast, ClO_4_^–^ and I^–^ that hardly bind in dimethyl sulfoxide show surprisingly significant affinities for the macrocycle in lipids. We reveal a lipid bilayer anion binding principle that depends on anion polarisability and bilayer penetration depth of complexes leading to unexpected advantages of charge-diffuse anions. These insights enhance our understanding of how biological systems select anions and guide the design of functional molecular systems operating at biomembrane interfaces.

## Abstract (web summary)

Quantification of anion binding by molecular receptors within lipid bilayers is challenging. Here, the authors synthesise a high-affinity macrocyclic anion receptor which provides insights into determinants of anion binding within lipid bilayers.

## Introduction

Molecular interactions at biomembrane interfaces^1,2^ are ubiquitous in many biological processes and underpin several mechanisms of drug action^3–7^. In particular, anion binding to membrane-embedded proteins and transmembrane anion transport facilitated by channels, carriers and pumps regulate the electrochemical gradients of Cl^–^, HCO_3_^–^, and organic anions which are fundamental to biological functions such as cell volume regulation, pH homoeostasis, solute transport and neurotransmission. Apart from proteins, a few small-molecule metabolites (e.g., prodigiosin^8^) and several classes of synthetic molecular receptors^9^ that contain electron-deficient anion binding cavities are known to facilitate anion transport across lipid bilayer membranes. These molecules have been proposed as putative therapeutics for ion channel diseases^3,7^ and cancer^4,6,10^.

Despite the advances in the area of anion receptor chemistry and transmembrane anion transport, fundamental knowledge of anion binding to natural or synthetic molecular receptors within lipid bilayers is lacking^11^. The majority of anion binding studies of molecular receptors are conducted in organic solvents and occasionally in aqueous solutions^12–15^, typically by NMR titration techniques. In lipid bilayers, however, difficulties including poor solubility of receptors, signal broadening and weak anion affinities have so far impeded the application of NMR or other techniques to elucidating anion binding for anion receptors within the membrane environments.

**Fig. 1:**
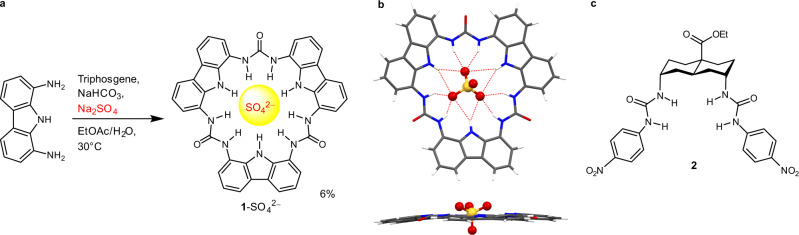
Compound structures, synthesis and crystal structure. **a** Synthesis of macrocycle **1**. The SO_4_^2–^ template could be removed by EtOAc/H_2_O extraction, which, however, led to partial degradation of the macrocycle. The free macrocycle with 80–90% purity was used in ^1^H NMR titrations in DMSO-*d*_6_/0.5% H_2_O. The pure SO_4_^2–^ complex was used in fluorescence and membrane transport studies in water where the complex completely dissociated at 50 nM due to the competitive aqueous conditions. **b** Top and side views of the crystal structure of the **1**–SO_4_^2–^ complex (CCDC: 2128483) with solvent removed by PLATON SQUEEZE and cation and disorder omitted for clarity. **c** Reference bis-urea anion receptor **2**.

In this work, we have successfully measured the anion binding affinities of a fluorescent macrocycle **1** (Fig. 1a) within lipid bilayers. This highly symmetric macrocycle has a ~3.5 Å cavity composed of a perfectly aligned array of nine strong NH hydrogen bond donors pointing inwards. This ensures hydrogen bonding interactions with large anions such as SO_4_^2–^ are sufficiently strong to be measurable in lipid bilayer environments, which we have found to be highly competitive. Here we report our discovery that despite the anion binding selectivity pattern of **1** strongly favouring charge-dense anions H_2_PO_4_^–^ and Cl^–^ in dimethyl sulfoxide (DMSO), when embedded within lipid bilayers, **1** demonstrates binding selectivity towards charge-diffuse ClO_4_^–^ and I^–^ anions (among monovalent anions). In lipid bilayers, preferential binding of charge-diffuse anions benefits from their higher polarisabilities and the deeper bilayer penetration of their complexes, the latter evidenced by fluorescence quenching studies using spin-labelled lipids. In comparison, the binding of charge-dense anions is hampered by severe electrostatic screening at the interfacial headgroup region. These findings correlate with the Hofmeister anion transport selectivity pattern typically observed for carrier-mediated transmembrane anion transport.

## Results and discussion

### Synthesis and crystal structure of macrocycle 1

The structure of trimeric carbazole-urea macrocycle **1** was initially proposed in a theoretical study in 2017^16^, but no synthetic progress was subsequently made despite the reported synthesis of the dimeric^17^ and the tetrameric^18^ analogues. We have developed a remarkably simple one-pot SO_4_^2–^-templated synthesis to access the uniquely flat and pre-organised trimeric macrocycle **1** (Fig. 1a). We obtained the crystal structure of the **1**–SO_4_^2–^ (Fig. 1b) complex modelled with severely disordered solvent molecules removed by PLATON SQUEEZE. The complex shows *D*_3d_ symmetry with a centrally bound SO_4_^2–^ ion forming six strong hydrogen bonds with the three urea units of **1** and additional stabilisation of the complex by six longer carbazole NH···SO_4_^2–^ hydrogen bonds. The macrocycle was slightly buckled (Fig. 1b right) to accommodate the large tetrahedral SO_4_^2–^ ion. Despite the limitation that the solvent molecules including those surrounding the cation could not be modelled, the crystal structure confirms the ability of **1** to bind a SO_4_^2–^ ion within its central cavity via multiple hydrogen bonds in the solid state.

### Anion binding in DMSO, micelles and lipid bilayers

**Fig. 2:**
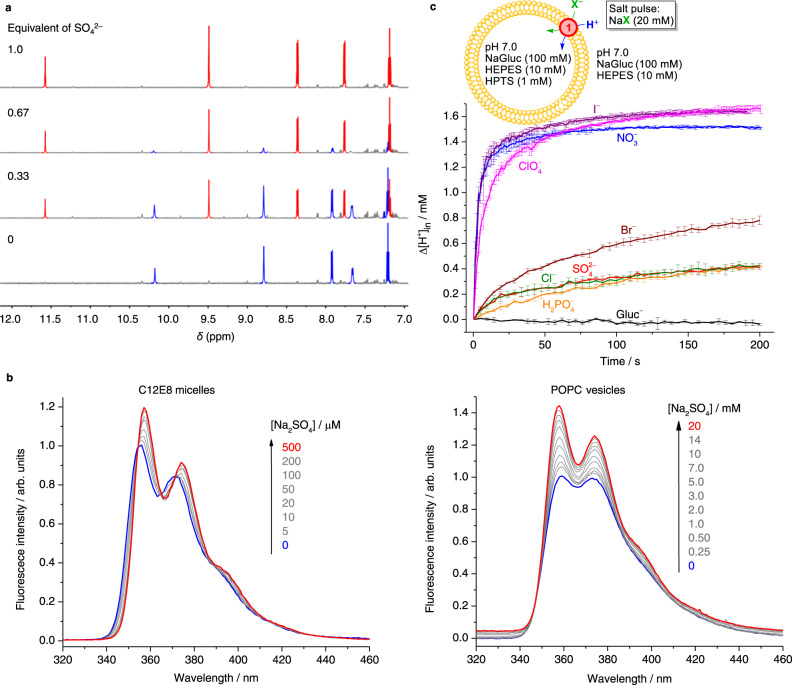
Anion binding and transmembrane anion transport by macrocycle 1. **a** ^1^H NMR (600 MHz) titration of **1** (0.3 mM) with TBA_2_SO_4_ in DMSO-*d*_6_/0.5% H_2_O. Signals from free **1** and the **1**–SO_4_^2–^ complex are shown in blue and red, respectively. Impurities are present from partial degradation of **1** during SO_4_^2–^ removal. **b** Fluorescence titration of **1** (50 nM) with Na_2_SO_4_ in C12E8 (2 mM) micelles (left) and POPC (0.2 mM) vesicles (right) in H_2_O. *λ*_ex_ = 265 nm. **c** Transmembrane H^+^/X^–^ symport (influx) facilitated by **1** (1 mol%) upon the external addition of a NaX (20 mM) salt in NaGluc vesicles buffered at pH 7.0 with 10 mM HEPES. Error bars represent SD from two experiments. C12E8 octaethylene glycol monododecyl ether, POPC 1-palmitoyl-2-oleoyl-sn-glycero-3-phosphocholine, NaGluc sodium gluconate, HEPES 4-(2-hydroxyethyl)-1-piperazineethanesulfonic acid, HPTS 8-hydroxypyrene-1,3,6-trisulfonate.

We conducted ^1^H NMR titrations of **1** with tetrabutylammonium (TBA^+^) salts of SO_4_^2–^, H_2_PO_4_^–^, Cl^–^, Br^–^, NO_3_^–^, I^–^ and ClO_4_^–^ in DMSO-*d*_6_/0.5% H_2_O (Table 1). Both SO_4_^2–^ (Fig. 2a) and H_2_PO_4_^–^ (Supplementary Fig. 14) lead to slow exchange ^1^H NMR responses, which were rarely observed^19^ in the relatively competitive organic solvent DMSO highlighting the anion binding potency of **1**. By contrast, the complexes of **1** with Cl^–^, Br^–^, NO_3_^–^ and I^–^ show fast exchange with the free macrocycle (Supplementary Figs. 5–12). The addition of ClO_4_^–^ even at >100 mM induced no discernible ^1^H chemical shift changes of **1** (Supplementary Fig. 13).

**Table 1:** Anion binding and transmembrane anion transport properties of compound **1**, along with literature values of anion hydration free energies and anion binding properties of compound **2** and PC vesicles

Anion	Δ*G*_hydr_ (kJ mol^–1^)^a^	Association constant *K*_a_ (M^–1^)					Transport rate by 1/anions s^–1^ carrier^–1 f^
		In DMSO-*d*_6_/0.5% H_2_O		In C12E8 micelles	In POPC vesicles	PC vesicles	
		1	2^b^	1	1		
SO_4_^2–^	–975	(7.4 ± 1.1)×10^9^	>10^5^	54,000 ± 3000	370 ± 10^c^	*ND*	0.042 ± 0.006
H_2_PO_4_^–^	–473	>10^5^	46,000	140 ± 20	<1	*ND*	0.031 ± 0.010
Cl^–^	–344	2000 ± 100	670	19 ± 1^c^	<1	0.2^d^	0.082 ± 0.016
Br^–^	–318	200 ± 10	70	29 ± 1^c^	2.6 ± 0.6^c^	2^d^	0.097 ± 0.018
NO_3_^–^	–286	340 ± 10	10	210 ± 10^c^	24 ± 4^c^	2.8^d^	2.1 ± 0.1
I^–^	–280	6.1 ± 0.6	3	200 ± 10^c^	24 ± 2^c^	32^e^	2.0 ± 0.4
ClO_4_^–^	–229	<1	*ND*	32 ± 1^c^	45 ± 9^c^	115^e^	0.83 ± 0.10

Errors represent SD from at least two experiments.

*ND* not determined.

^a^Gibbs energies of hydration at 25 °C, compiled by Marcus^25^.

^b^Reported by Jurček et al.^20^.

^c^Ionic strength fixed at 0.2 M.

^d^Reported by Tatulian^34^, using egg PC vesicles.

^e^Reported by Rydall and Macdonald^33^, using POPC vesicles.

^f^Determined at an anion concentration of 20 mM.

Macrocycle **1** has an exceptionally strong SO_4_^2–^ affinity in the sub-nanomolar range, which necessitates the use of a BaSO_4_ precipitation method for quantification. For monovalent anions, the anion binding affinity decreases in the order of H_2_PO_4_^–^ > Cl^–^ > NO_3_^–^ > Br^–^ > I^–^ > ClO_4_^–^ (Table 1). Compared with Davis’s acyclic bis-urea **2** (Fig. 1c)^20^, the macrocyclic receptor confers a modest 1–2 fold affinity enhancement for Cl^–^, Br^–^ and I^–^, but a significant 33-fold enhancement for NO_3_^–^. Geometrical optimisation of the anion complexes is evidence in support of the hypothesis  that **1** has the perfect size and shape complementary fit for NO_3_^–^ resulting in a precisely flat and *D*_3h_-symmeric complex (Supplementary Fig. 4b)^16^. By contrast, the macrocyclic cavity is slightly too large for I^–^ (Supplementary Fig. 4c) and consequently much too large for Br^–^ (Supplementary Fig. 4d) and Cl^–^ (Supplementary Fig. 4e). Notably, here without using **2** as a reference receptor for comparison, the macrocycle’s structural preference for NO_3_^–^ would have been lost in the binding constant data in DMSO, as charge-dense^21^ monovalent anions H_2_PO_4_^–^ and Cl^–^ have much greater affinities than NO_3_^–^ which only narrowly edges out Br^–^. Thus, in DMSO the anion binding affinities of **1** are dominated by the strength of electrostatic interactions leading to favourable binding of charge-dense anions. Interestingly, for a recently reported hexapodal cage that binds anions via less acidic alkyl amide NH and imine CH sites^19^, a weaker SO_4_^2–^/I^–^ selectivity of ~30 and a I^–^ > Cl^–^ selectivity were found in DMSO-*d*_6_ contrasting the behaviour of macrocycle **1** that binds anion via highly acidic aromatic urea and carbazole NH sites. This suggests that the extent of preference for charge-dense anions is influenced by the acidity of anion binding sites.

**Fig. 3:**
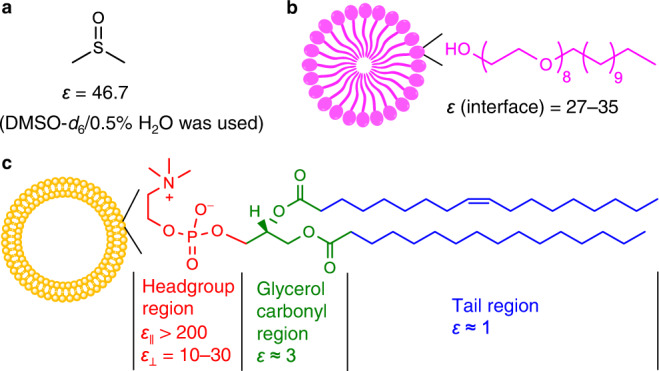
Dielectric properties of three media used for anion binding studies in this paper. **a** DMSO. **b** C12E8 micelles, where the interfacial dielectric constant was determined by Drummond et al. ^24^ based on p*K*_a_ shifts of lipoidal pH indicators. **c** POPC vesicles with theoretically modelled dielectric constants. The dielectric constants of the headgroup region were taken from Stern & Feller^28^ and Raudino & Mauzerall^29^, and the values of the remaining regions from Nymeyer & Zhou^27^.

To evaluate the anion binding strength of **1** in water, we next switched the medium from DMSO to non-ionic octaethyleneglycol monododecyl (C12E8, Fig. 3b left) micelles dispersed in water^22^, as neither **1** nor its anion complex is soluble in pure water. Macrocycle **1** can be solubilised at sub-μM concentrations in C12E8 micelles and demonstrates a fluorescence enhancement response to SO_4_^2–^ (Fig. 2b), giving a remarkable SO_4_^2–^ binding constant of 5.4 × 10^4^ M^–1^ in water.

Apart from H_2_PO_4_^–^ that induced a fluorescence response similar to SO_4_^2–^ (Supplementary Fig. 19), other anions produced either negligible fluorescence responses (Cl^–^) or fluorescence quenching responses (Br^–^, NO_3_^–^, I^–^ and ClO_4_^–^) partly attributable to dynamic quenching (Supplementary Fig. 21), rendering direct fluorescence titrations unfeasible. Instead, we conducted competition titrations with SO_4_^2–^ in the presence of these anions and calculated the affinities based on the attenuation of SO_4_^2–^ affinity using a competition binding model. In these analyses, it was necessary to correct the binding constant values against Boltzmann factors as anions adsorb to micellar surfaces^23^ leading to a negative surface potential (estimated by electrophoretic measurements) which then reduces the SO_4_^2–^ concentration at the surface by a Boltzmann factor compared within the bulk solution.

Table 1 demonstrates a modified anion selectivity in water/C12E8 compared with in DMSO, despite SO_4_^2–^ remaining as the most strongly bound anion. While H_2_PO_4_^–^, Cl^–^ and NO_3_^–^ are the top-3 strongest binding monovalent anions in DMSO, Cl^–^ drops out of this group in the water/C12E8 system and is replaced by I^–^. We rationalise the binding data on the basis of anion solvation free energies and medium polarity (Fig. 3). The interfacial dielectric constant of C12E8 micelles was estimated to be ~27–35^24^, which is similar to that of MeCN and lower than DMSO, and hence anion binding should not be weakened solely on medium polarity considerations. Here the diminished affinities of charge-dense SO_4_^2–^, H_2_PO_4_^–^ and Cl^–^ anions, and the shift of anion selectivity towards more charge-diffuse NO_3_^–^ and I^–^ anions are attributed to the heavier dehydration costs of charge-dense than charge-diffuse anions^25^. The augmented affinities of I^–^ and ClO_4_^–^ in water/C12E8 than in DMSO despite the anion dehydration cost in water/C12E8 can be rationalised by the high receptor desolvation cost in DMSO (see Supplementary Fig. 11 for evidence of desolvation)^14^. We have also performed ^1^H NMR of macrocycle **1** in water/C12E8 in the presence of anions (Supplementary Fig. 29), in which the observation of resonances from anion complexes provided unambiguous evidence of binding of all tested anions in the biphasic system.

Importantly, macrocycle **1** also demonstrated a fluorescence enhancement response to SO_4_^2–^ (Fig. 2b right) when incorporated at sub-μM concentrations in POPC (Fig. 3c) vesicles suspended in water. A further reduced SO_4_^2–^ affinity of 370 M^–1^ was found for **1** in POPC vesicles compared with in C12E8 micelles (Table 1). This attenuation could be in part due to competitive receptor binding to the phosphate headgroup of POPC^26^. To gain more evidence for this, we performed SO_4_^2–^ titrations of **1** in 10% and 20% POPC/C12E8 mixed micelles, which demonstrated 4.5-fold and 9-fold reduced SO_4_^2–^ affinities (Supplementary Figs. 19 and 20), respectively, compared with in pure C12E8 micelles. Further evidence was provided by direct observation of ^1^H NMR signals corresponding to the **1**–POPC complex in POPC/C12E8 mixed micelles (Supplementary Fig. 30).

Examination of binding constants of other anions in POPC vesicles (determined by competition binding experiments with surface potential effects corrected, Table 1) has however revealed a trend that cannot be explained solely by competitive headgroup binding which does not impact anion selectivity. Strikingly, while the divalent SO_4_^2–^ always remained the strongest binding anion, the top-three monovalent anion group changed again compared to C12E8 micelles, with H_2_PO_4_^–^ being knocked out by ClO_4_^–^ which joins NO_3_^–^ and I^–^. In POPC vesicles, H_2_PO_4_^–^ and Cl^–^ no longer showed appreciable affinities (*K*_a_ < 1 M^–1^). This trend also cannot be explained by anion dehydration costs alone, as anion binding is subject to the dehydration requirement in both water/C12E8 and water/POPC systems. The dielectric property of the lipid bilayers, on the other hand, could provide a clue to understanding the enhanced selectivity for charge-diffuse anions in POPC vesicles than in C12E8 micelles. Previously molecular dynamics simulation studies have estimated the dielectric constant of the zwitterionic headgroup region to be higher than that of bulk water (*ε*_||_ > 200, where *ε*_||_ is the dielectric constant component parallel to the bilayer surface and affects half of the NH-binding sites on average assuming perpendicular insertion of the macrocycle; the perpendicular component *ε*_⊥_ was estimated to be ~10–30, which affects the remaining half of the NH sites)^27–29^. Flood and coworkers have shown in a Cl^−^-binding macrocycle that with a higher solvent dielectric constant, the energetic contribution of electrostatic interactions reduces while non-electrostatic induction and dispersion contributions start to dominate^13^. Thus the reason that should anion binding occur at the high dielectric constant headgroup region, charge-dense anions SO_4_^2–^, H_2_PO_4_^–^ and Cl^–^ that mainly rely on electrostatic interactions to bind would be disadvantaged over large charge-diffuse anions ClO_4_^–^ and I^–^ that have favourable induction and dispersion terms due to their polarisabilities. This effect would add to the chaotropic preference that arises from dehydration cost alone as we have already seen in C12E8 micelles.

### Location of macrocycle 1 in lipid bilayers

Key information on the location of anion binding in POPC vesicles was then obtained by fluorescence penetration-depth studies using spin-labelled lipids to quench the fluorescence of **1** at different locations (Supplementary Figs. 43–46)^30^. Without anions, the most probable location of macrocycle **1** was determined to be 19 Å from the bilayer centre, corresponding to the headgroup region. This is consistent with the hypothesis that free anion receptors can bind to the phosphate headgroup of lipids^26^. Macrocycle **1** remains at the headgroup region upon binding to SO_4_^2–^, but upon binding to ClO_4_^–^ penetrates deeper (16 Å) into the carbonyl/glycerol region with a lower dielectric constant of 3–4^27^. Here we have confirmed that the binding of SO_4_^2–^ occurs at the high dielectric constant headgroup region, supporting the hypothesis that the binding of charge-dense anions is subject to severe electrostatic screening which diminishes their affinities (note that the SO_4_^2–^ selectivity persists but is much weaker than in DMSO and in C12E8 micelles). We have shown an additional benefit for charge-diffuse anions such as ClO_4_^–^ that their complexes (and likely also the free anions^31,32^) can penetrate deeper into a more hydrophobic microenvironment where anion binding is enhanced.

It is of interest to compare the anion binding properties of macrocycle **1** in lipids against anion binding by lipids themselves. Lipid bilayers preferentially adsorb charge-diffuse anions and exhibit a Hofmeister selectivity pattern of ClO_4_^–^ > I^–^ » NO_3_^–^ > Br^–^ > Cl^–^ > H_2_PO_4_^–^ (see also Supplementary Table 3)^33,34^. Table 1 shows that macrocycle **1** binds Br^–^, I^–^ and ClO_4_^–^ with similar or weaker affinities than lipids, but binds NO_3_^–^ ~8 times more strongly than lipids, again manifesting the perfect size and shape matching of the macrocycle for NO_3_^–^ (Supplementary Fig. 3b). The ability of lipids to preferentially accumulate ClO_4_^–^ below the headgroup region^31,32^, on the other hand, has overwhelmed the macrocycle’s preference for NO_3_^–^.

Further information came to light when we compare the abovementioned phenomena to cation binding to lipids and to the cation receptor/carrier valinomycin in lipids. The cation affinities of PC lipids among alkali metal cations from Li^+^ to Rb^+^ are very similar^35^, while being far weaker than lipids binding charge-diffuse anions^33,34^. For valinomycin, although cation binding affinities dropped by several orders of magnitude when the medium switched from organic solvents to lipids, no drastic alteration of cation selectivity was found in lipids^36^ in contrast to the behaviour of “anti-valinomycin” **1**. As shown by previous theoretical investigations, the greater polarisability of large charge-diffuse anions^37^ is central to their strong interfacial adsorption^38,39^ and in the cases of water/lipid interfaces, this then benefits anion binding to an anion receptor embedded in lipids due to increased local anion concentrations, in addition to highly polarisable anions having favourable induction and dispersion interactions with an anion receptor. This effect is absent in cation binding because of the poor polarisabilities of cations.

### Transmembrane anion transport by macrocycle 1

Finally, to gain a better understanding of the biomedically relevant topic of carrier-mediated anion transport^4,6,7,9,10^ based on our current findings, we studied macrocycle **1** as an anion transporter in POPC vesicles (Fig. 2c)^40^. Macrocycle **1** functions as an H^+^/anion^–^ symporter but not as an anion uniporter (Supplementary Fig. 50) presumably due to the strong headgroup binding that inhibited transmembrane diffusion of the free receptor^26^. An anion transport selectivity of NO_3_^–^ ≈ I^–^ > ClO_4_^–^ > Br^–^ > Cl^–^ > SO_4_^2–^ > H_2_PO_4_^–^ was observed by initial rate comparison (Table 1, Fig. 2c), which correlates with, but is not identical to the anion binding selectivity in lipids. Carrier-mediated ion transport rates depend both on the ion binding affinity and the rate of ion-carrier complex diffusion through the membrane^41^, the latter being unfavourable for the doubly charged SO_4_^2–^. While NO_3_^–^ and I^–^ can be fully embedded into the macrocyclic plane (Supplementary Fig. 4b, c), ClO_4_^–^ has an exposed oxygen atom after binding to **1** (Supplementary Fig. 4a), likely slowing down ClO_4_^–^ transport than the transport of NO_3_^–^ and I^–^. In light of the ClO_4_^–^, I^–^ » NO_3_^–^ transport selectivity commonly observed for structurally simple hydrogen bond-based anion transporters following Hofmeister series^42^, here the clear NO_3_^–^ > ClO_4_^–^ transport selectivity of **1** again reflects macrocycle’s structural fit for NO_3_^–^, which, however, is insufficient to confer a significant NO_3_^–^ > I^–^ selectivity due to the preference of the lipid environment for the more hydrophobic I^–^. Our results in lipid bilayers thus explain the difficulty^42^ of overcoming the Hofmeister bias to facilitate selective membrane transport of more hydrophilic anions such as Cl^–^ which shows a deceptively strong affinity of 2000 M^–1^ for **1** in DMSO. In addition, as high-efficacy anion transporters typically have Cl^–^ affinities in the range of 10^2^–10^4^ M^–1^ in DMSO^7^, our results imply that those systems likely bind Cl^–^ with low affinities of <10 M^–1^ in lipid bilayers and hence should operate far from ion binding saturation when transporting Cl^–^ under physiologically relevant conditions.

In summary, we have gained access to the intricacies of anion binding at biomembrane interfaces taking advantage of a strong SO_4_^2–^ binding macrocycle **1** showing fluorescence perturbation upon binding SO_4_^2–^ at interfaces. We show that in organic solvents such as DMSO, electrostatic effects dominate leading to preferential binding of charge-dense anions. In biphasic systems with a moderate interfacial polarity such as non-ionic micelles, both electrostatic and dehydration effects operate such that a range of anions across the Hofmeister scale can bind. Contrastingly, we show that anion binding in lipid bilayers behaves differently from the above two scenarios in that anion polarisability, electrostatic screening and penetration depth underlie anion binding strength/selectivity leading to surprisingly favourable binding of charge-diffuse anions, in particular, ClO_4_^–^. In all tested media, we have seen the intrinsic size/shape matching selectivity of **1** for NO_3_^–^ struggling to manifest itself amid the electrostatic, solvation and polarisability effects characteristic of the anions and medium conditions. The elucidation of anion binding principles at lipid bilayers is important to diverse research topics ranging from ion interactions with membrane-embedded proteins/peptides to the development of drug delivery vehicles and synthetic receptors/transporters/assemblies functioning at biomembrane interfaces.

## Methods

### General

All reagents were purchased from commercial sources and used as received. NMR spectra were recorded on a Bruker Avance III 400 NMR Spectrometer equipped with a BBFO room temperature probe or a Bruker Avance III 600 NMR Spectrometer equipped with a TCI cryoprobe. High-resolution mass spectra (HRMS) were recorded on a Bruker Apex Qe 7 T Fourier Transform Ion Cyclotron Resonance Mass Spectrometer. Inductively coupled plasma mass spectrometry (ICP-MS) was acquired on a Perkin Elmer Nexion 350X Inductively Coupled Plasma Mass Spectrometer. Fluorescence studies were performed on a Horiba Fluoromax-4 or an Agilent Cary Eclipse Fluorescence Spectrometer equipped with a magnetic stirrer and a temperature controller. Dynamic light scattering (DLS) and electrophoretic mobility measurements were performed on a Malvern Zetasizer Nano ZS. Single crystal X-ray diffraction was performed on a Bruker APEX-II CCD diffractometer.

### ^1^H NMR titrations in DMSO-d_6_/0.5% H_2_O

A DMSO-*d*_6_ stock solution of free macrocycle **1** was diluted to 0.3 mM in 0.5 mL of DMSO-*d*_6_/0.5% H_2_O. The solution was titrated against TBA^+^ salts of anions and the ^1^H NMR spectra were acquired. The chemical shifts of the NH and CH resonances were plotted against the anion concentration and the data were fitted to a suitable binding model (1:1 or 1:2 host–guest) to determine the binding constants. For SO_4_^2–^, a competition BaSO_4_ precipitation method was used due to strong binding. See Supplementary Information for details.

### Fluorescence titrations in C12E8 micelles and POPC vesicles

A DMSO stock solution of **1**–SO_4_^2–^ complex (10 μM) was diluted to 50 nM in 2.5 mL of H_2_O containing C12E8 (2 mM) micelles or POPC (0.2 mM) vesicles, the latter extruded with 100 nm polycarbonate membranes. The solution was titrated against Na_2_SO_4_ and the fluorescence spectra were recorded. The fluorescence intensity at 357 nm (in C12E8 micelles) or 358 nm (in POPC vesicles) was plotted against the anion concentration and fitted to a 1:1 binding model to calculate the SO_4_^2–^ binding constant.

For both systems (C12E8 micelles and POPC vesicles), the SO_4_^2–^ titration was also performed in the presence of a competing anion (as a Na^+^ salt, with the ionic strength fixed at 0.2 M) to indirectly determine the binding constant of the competing anion using a competition binding model. The zeta potentials of C12E8 micelles and POPC vesicles in the presence of the competing anion were determined by electrophoretic measurements, from which the surface potential values were estimated based on the Gouy–Chapman model^43^. The Boltzmann factor for SO_4_^2–^ calculated from the surface potential value was used to correct the binding data against the surface potential effect. See Supplementary Information for details.

### Transmembrane anion transport

A “salt-pulse” assay was used to determine the rate of H^+^/anion symport facilitated by macrocycle **1**. A DMSO solution of **1**–SO_4_^2–^ complex (final concentration 1 μM) was added to a suspension of POPC (0.1 mM) vesicles (diameter ~200 nm) loaded with and suspended in sodium gluconate (NaGluc, 100 mM) buffered at pH 7.0 with HEPES (10 mM). NaX (20 mM, X^–^ = Gluc^–^, H_2_PO_4_^–^, Cl^–^, Br^–^, NO_3_^–^, I^–^ or ClO_4_^–^) or Na_2_SO_4_ (20 mM) was added to the vesicle suspension. H^+^/anion symport facilitated by macrocycle **1** leads to intra-vesicular acidification monitored by an intra-vesicular fluorescence pH indicator HPTS. The fluorescence data were converted to H^+^ influx (Δ[H]_in_) as detailed in Supplementary Information.

## Supplementary information

Supplementary Information

Description of Additional Supplementary Files

Supplementary Data 1

## Data Availability

Data supporting the findings of this study are available from the manuscript and its supplementary information. Supplementary Data File 1 contains the xyz coordinates for the optimised structures. Files containing the raw data have been deposited in figshare with the 10.6084/m9.figshare.20155097 accession code. The crystal structure of **1**-SO_4_^2–^ complex has been deposited at the Cambridge Crystallographic Data Centre (CCDC), under deposition number 2128483. These data can be obtained free of charge from The Cambridge Crystallographic Data Centre via www.ccdc.cam.ac.uk/data_request/cif.
