# Supplementary material for: Measuring anion binding at biomembrane interfaces
Source: Nat Commun. 2022 Aug 8;13:4623. doi: 10.1038/s41467-022-32403-z (PMC9359984; doi:10.1038/s41467-022-32403-z)
Supplement: Supplementary file 1 — Supplementary Information [file 41467_2022_32403_MOESM1_ESM.pdf]

**Supplementary Information for**

# Measuring Anion Binding at Biomembrane Interfaces

Xin Wu[a], Patrick Wang[a], William Lewis[a], Yun-Bao Jiang[b], and Philip A. Gale[a,*]

[a] School of Chemistry, The University of Sydney, NSW 2006, Australia
[b] Department of Chemistry, College of Chemistry and Chemical Engineering, The MOE Key Laboratory of Spectrochemical Analysis and Instrumentation, and *i*ChEM, Xiamen University, Xiamen 361005, China

[*]Corresponding author. E-mail address: philip.gale@sydney.edu.au

## Table of Contents

Supplementary Methods ........ S2
S1. Synthesis of **1** ........ S2
S3. X-ray crystallography ........ S3
S4. Computational modelling ........ S5
S5. $^1$H NMR titrations in DMSO ........ S6
S5.1 $^1$H NMR titrations with non-sulfate anions ........ S6
S5.2 Determination of sulfate affinity ........ S13
S6. Fluorescence titrations in C12E8 micelles ........ S15
S6.1 Determination of sulfate and phosphate affinity ........ S15
S6.2 Determination of affinities of other anions ........ S18
S7. $^1$H NMR evidence of anion binding in C12E8 micelles ........ S23
S7.1 Evidence of anion binding ........ S23
S7.2 Evidence of lipid headgroup binding ........ S25
S8. Fluorescence titrations in POPC vesicles ........ S26
S8.1 Determination of sulfate affinity ........ S26
S8.2 Determination of affinities of other anions ........ S27
S9. Control fluorescence studies ........ S32
S10. Fluorescence penetration depth studies ........ S34
S11. Transmembrane anion transport ........ S37
S11.1 HPTS assay ........ S37
S11.2 Osmotic response assay ........ S39
Supplementary References ........ S41

# Supplementary Methods

## S1. Synthesis of **1**

$Na_2SO_4$ (24 g) and $NaHCO_3$ (0.15 g, 1.8 mmol) were dissolved in $H_2O$ (80 mL) at 30 °C. To this aqueous solution was added an EtOAc solution (40 mL) of 1,8-diaminocarbazole[1] (79 mg, 0.4 mmol) to form a biphasic mixture. Under vigorous stirring at 30 °C, triphosgene (83 mg, 0.28 mmol) was added to the biphasic mixture, and the stirring continued for 3 h at 30 °C. Afterwards, the organic phase which contained precipitates was separated from the aqueous phase using a separation funnel and carefully loaded into a prep-TLC plate (Analtech 02013) with frequent drying under a stream of $N_2$ gas. The plate was initially run with MeCN for two hours to push the amine starting material to the top of plate, and then dried in a fumehood overnight. Triethylamine (TEA) was then pipetted into the baseline, and the prep-TLC was run with 9:1 (v:v) MeCN-TEA which led to separation of macrocycle **1** as a brown band from the baseline. The TEA treatment and the run with 9:1 (v:v) MeCN-TEA were repeated to isolate more macrocycle **1** from the baseline. After the plate was dry, the product band was scrapped and then treated with DMF (15 mL) to retrieve the product. The silica gel was removed by filtration and the DMF solution was evaporated under a stream of $N_2$ gas overnight. The residue was triturated with EtOAc (2 mL) and dried under vacuum to give the **1**-$SO_4^{2-}$ complex as a grey powder (10 mg, 6%). The product contained 3–4 equivalents of TEA (Supplementary Figure 1), which could not be removed by vacuum drying or EtOAc trituration suggesting their strong interactions in the solid-state. Although $Na_2SO_4$ was used as the template, the $Na^+$ ions exchanged with metal ion impurities in the silica gel during prep-TLC separation. Therefore, the cationic component of the **1**-$SO_4^{2-}$ complex was a mixture of metal ions, determined to be (in mol%) 75% Zn, 12% Na, 7% Ca, 2% Mn, 2% Fe, 1% Al and 1% Mg by ICP-MS.

$^1H$ NMR (400 MHz, DMSO-$d_6$) δ 11.59 (s, 3H, carbazole-NH), 9.50 (s, 6H, urea-NH), 8.37 (d, $J$ = 7.7 Hz, 6H, ArH), 7.76 (d, $J$ = 7.7 Hz, 6H, ArH), 7.19 (t, $J$ = 7.8 Hz, 6H, ArH), 2.98 (d, $J$ = 6.5 Hz, 22H, TEA-$CH_2$), 1.12 (t, J = 7.2 Hz, 33H, TEA-$CH_3$).

$^{13}C$ NMR (101 MHz, DMSO-$d_6$) δ 152.18, 129.43, 125.08, 123.64, 119.62, 113.66, 113.10, 45.76, 9.03.

HRMS (ES$^-$) $m/z$: $[M+SO_4]^{2-}$ calculated 388.5883, found 388.5886.

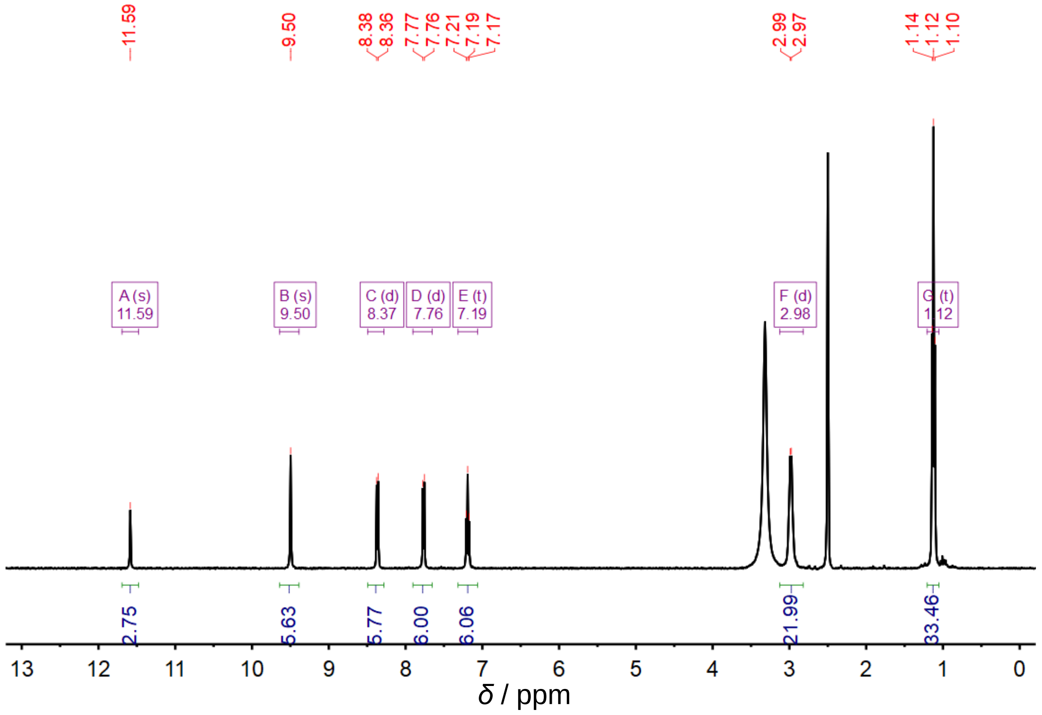


**Supplementary Figure 1** $^1H$ NMR (400 MHz) of **1**-$SO_4^{2-}$ in DMSO-$d_6$.

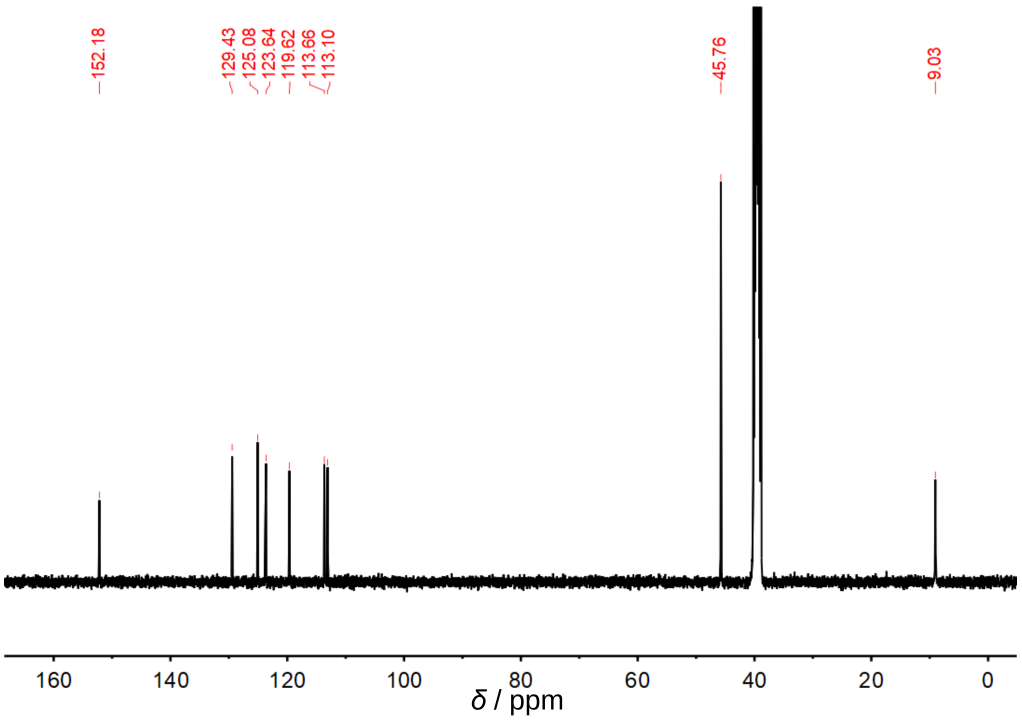


**Supplementary Figure 2** $^{13}C$ NMR (101 MHz) of **1**-$SO_4^{2-}$ in DMSO-$d_6$.

## S2. X-ray crystallography

Single crystals of the **1**-$SO_4^{2-}$ complex (CCDC 2128483) was obtained by slow vapour diffusion of $Et_2O$ into an MeCN solution of **1**-$SO_4^{2-}$. A suitable crystal was selected and mounted with paratone on a MiTeGen Micromount. Data collection was performed on a Bruker APEX-II CCD diffractometer at 100(2) K. The highly disordered solvent molecules were removed using PLATON SQUEEZE. Using Olex2[2], the structure was solved with the olex2.solve[3] structure solution program using Charge Flipping and refined with the SHELXL[4] refinement package using Least Squares minimisation.

**Supplementary Table 1 Crystal data and structure refinement.**

| | |
|---|---|
| Empirical formula | $C_{61}H_{93}MgN_9O_{18}S$ |
| Formula weight | 1296.81 |
| Temperature / K | 100(2) |
| Crystal system | cubic |
| Space group | $I\bar{4}3m$ |
| $a$ /Å | 25.0198(18) |
| $b$ / Å | 25.0198(18) |
| $c$ / Å | 25.0198(18) |
| $\alpha$ / ° | 90 |
| $\beta$ / ° | 90 |
| $\gamma$ / ° | 90 |
| Volume / Å$^3$ | 15662(3) |
| $Z$ | 8 |
| $\rho_{calc}$ g /cm$^3$ | 1.100 |
| $\mu$ / mm$^{-1}$ | 0.113 |
| $F$(000) | 5552.0 |
| Crystal size / mm$^3$ | 0.1 × 0.1 × 0.1 |

| | |
|---|---|
| Radiation | Mo Kα ($\lambda$ = 0.71073) |
| $2\theta$ range for data collection / ° | 2.302 to 50.08 |
| Index ranges | $-29 \leq h \leq 25$, $-29 \leq k \leq 10$, $-16 \leq l \leq 27$ |
| Reflections collected | 38445 |
| Independent reflections | 2551 [$R_{\mathrm{int}}$ = 0.0731, $R_{\mathrm{sigma}}$ = 0.0377] |
| Data/restraints/parameters | 2551/177/104 |
| Goodness-of-fit on $F^2$ | 1.411 |
| Final $R$ indexes [$I >= 2\sigma (I)$] | $R_1$ = 0.1394, $wR_2$ = 0.3456 |
| Final $R$ indexes [all data] | $R_1$ = 0.2281, $wR_2$ = 0.4390 |
| Largest diff. peak/hole / e Å$^{-3}$ | 0.62/–0.44 |
| Flack parameter | –0.13(16) |

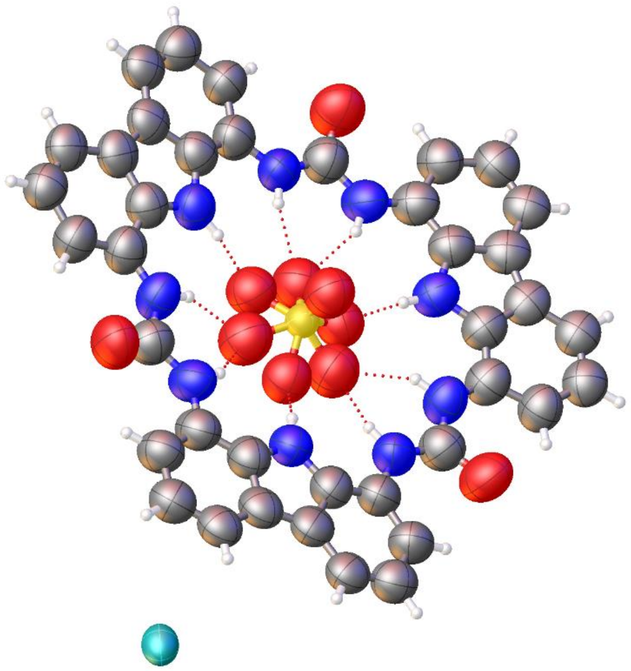

**Supplementary Figure 3** Crystal structure of **1**-$SO_4^{2-}$ with a $Mg^{2+}$ counterion and thermal ellipsoids shown at 50% probability. The solvents surrounding the cation could not be modelled due to severe disorder.

# S3. Computational modelling

Complexes of **1** with $ClO_4^-$, $NO_3^-$,[5] $I^-$,$Br^-$ and $Cl^-$ and were optimised at the B3LYP/6-31G* level of theory, using Spartan'14. As shown in Supplementary Figure 4, $NO_3^-$ fits perfectly into the macrocyclic cavity forming strong hydrogen bonds with all NH donors of **1** (b), leading to a perfectly flat and $D_{3h}$-symmetric macrocycle, consistent with Mooibroek et al.[5] Although $ClO_4^-$ also fits well (a), the macrocycle is slightly buckled and one of the oxygen atoms of $ClO_4^-$ does not interact with the macrocycle. The three halide ions are too small for strong hydrogen bonding interactions with the macrocycle (c–e) as the NH donors only slightly (c, d) or hardly (e) overlap with the van der Waals sphere of the central halide ion.

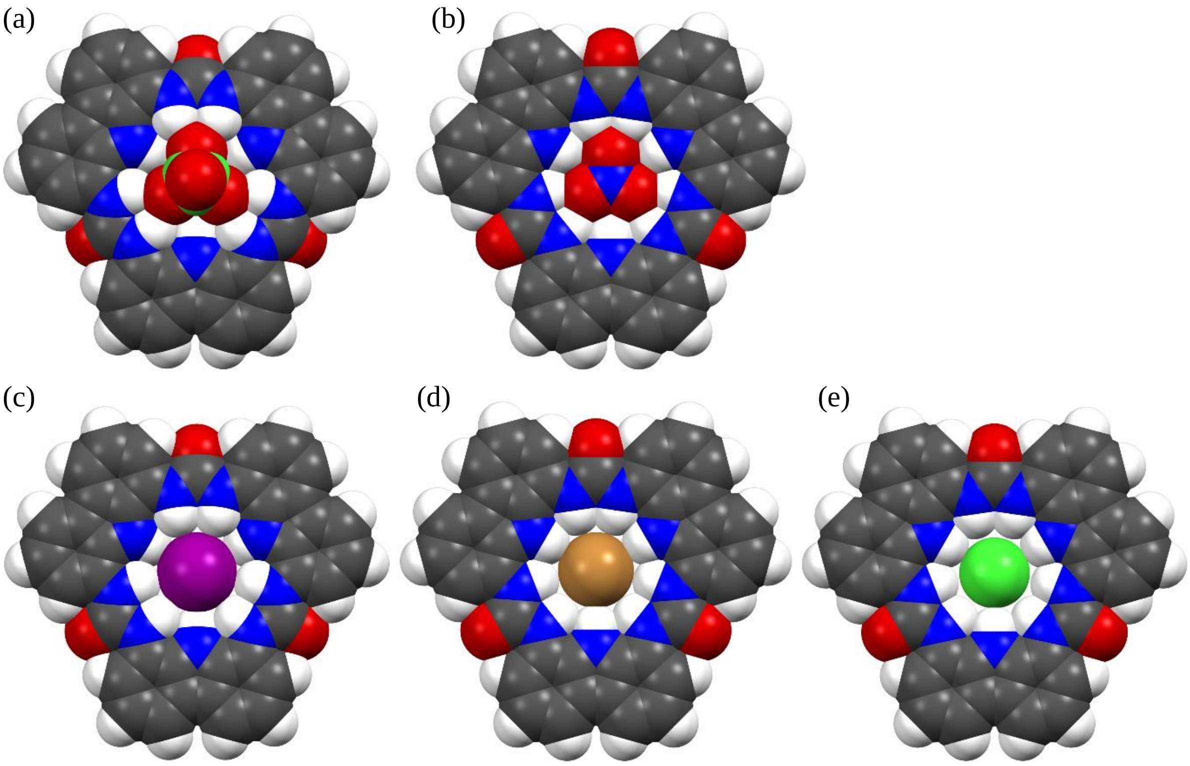


**Supplementary Figure 4** Optimised structures of complexes of **1** with $ClO_4^-$ (a), $NO_3^-$ (b), $I^-$ (c), $Br^-$ (d) and $Cl^-$ (e).

# S4. $^1$H NMR titrations in DMSO

## S4.1 $^1$H NMR titrations with non-sulfate anions

$^1$H NMR titrations of free macrocycle **1** with $TBA^+$ salts of anions were performed in DMSO-$d_6$/0.5% $H_2O$. To obtain free macrocycle **1**, an EtOAc solution of **1**-$Na_2SO_4$ obtained after the reaction (see Section S1) was filtered to remove insoluble by-products and then the $Na_2SO_4$ was removed by extraction with Milli-Q water (40 mL). The EtOAc phase was evaporated at room temperature, and then the residue (free macrocycle **1**) was immediately dissolved in DMSO-$d_6$ (0.5 mL) and stored in a freezer for use in $^1$H NMR titrations. Storage as a DMSO solution is necessary because free macrocycle **1** is unstable either as an EtOAc solution or in the solid state, but gains stability in DMSO likely due to the strong hydrogen bond accepting ability of DMSO. Because of partial degradation of the macrocycle in EtOAc upon $Na_2SO_4$ removal, the free macrocycle was 80–90% pure. This does not affect the validity of $^1$H NMR binding studies because the signals from **1** and from impurities are well separated. The concentration of **1** in the DMSO-$d_6$ stock solution was unknown, so this was determined by $^1$H NMR integration against TBACl (as an internal reference) added to a 50-fold diluted DMSO-$d_6$ solution of **1**. For each titration experiment, a 0.3 mM solution of free macrocycle **1** was prepared in DMSO-$d_6$ with 0.5% (vol%) of added $H_2O$, and then $^1$H NMR spectra in the absence and presence of increasing concentrations of $TBA^+$ salts of anions were acquired. The chemical shifts of the carbazole NH, urea NHs and one aromatic CH that was the most sensitive to anion addition among the three CHs were plotted against the anion concentration. In the case of $NO_3^–$, the aromatic CH was not used for analysis because it converged and then swapped positions with another aromatic CH during the titration. Data fitting was performed using online software Bindfit[6] to calculate the binding constants.

It should be noted that we found the commercial DMSO-$d_6$ to contain ~15 μM of $SO_4^{2–}$ impurities, and therefore signals of **1**-$SO_4^{2–}$ complex (in slow exchange with other species) were always observed except with excess of $HPO_4^{2–}$ which could fully displace $SO_4^{2–}$ from **1** (Supplementary Figure 14 top spectrum).

Selection of the binding model (1:1 or 1:2) was based on the observed shape of the binding isotherm, as well as the knowledge of the charge-density of the anions and their extent of size/shape-matching with the macrocycle. In the case of $NO_3^–$, the binding isotherm is characteristic of a 1:1 equilibrium with saturation observed at high anion concentrations (Supplementary Figure 10). This, in combination with the perfect size/shape-matching of $NO_3^–$ with the macrocycle (Supplementary Figure 4b) and the weak charge-density of $NO_3^–$ renders the binding of a second $NO_3^–$ (which does not benefit from macrocyclic pre-organisation and multivalency) unlikely, justifying the use of the 1:1 binding model. By contrast, for more charge-dense and less structurally fitting $Cl^–$ and $Br^–$ anions (Supplementary Figures 4c, 4e), some of the $^1$H signals continued shifting at high anion concentrations after initially approaching saturation (Supplementary Figures 6, 8), indicating a stepwise 1:2 equilibria. This is plausible because the affinity of the first $Cl^–$/$Br^–$ is compromised by the poor structural fitting, whereas the binding of second anion is more pronounced than in the case of $NO_3^–$ due to higher charge densities of $Cl^–$ and $Br^–$. For $I^–$, the 1:1 model was used as the low affinity renders the binding of a second $I^–$ unlikely to be significant at the tested concentration range.

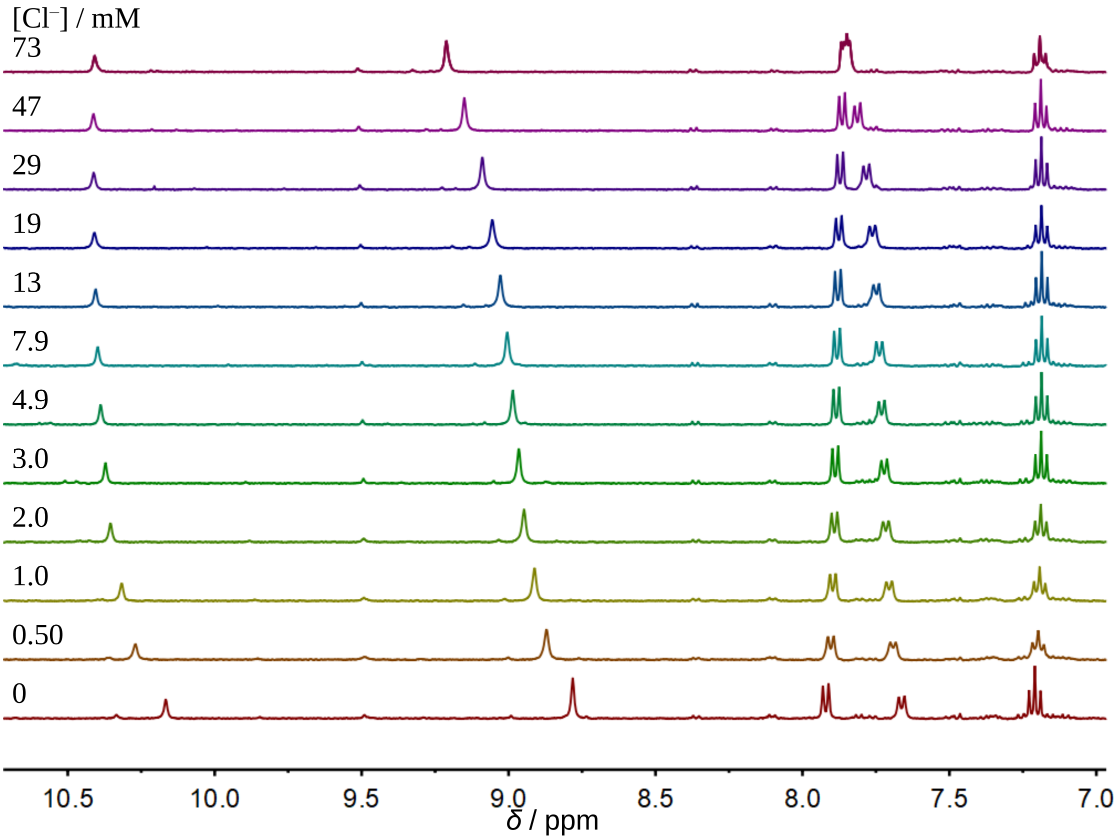


**Supplementary Figure 5** $^{1}$H NMR (400 MHz) titration of **1** (0.3 mM) with TBACl in DMSO-$d_6$/0.5% $H_2O$.

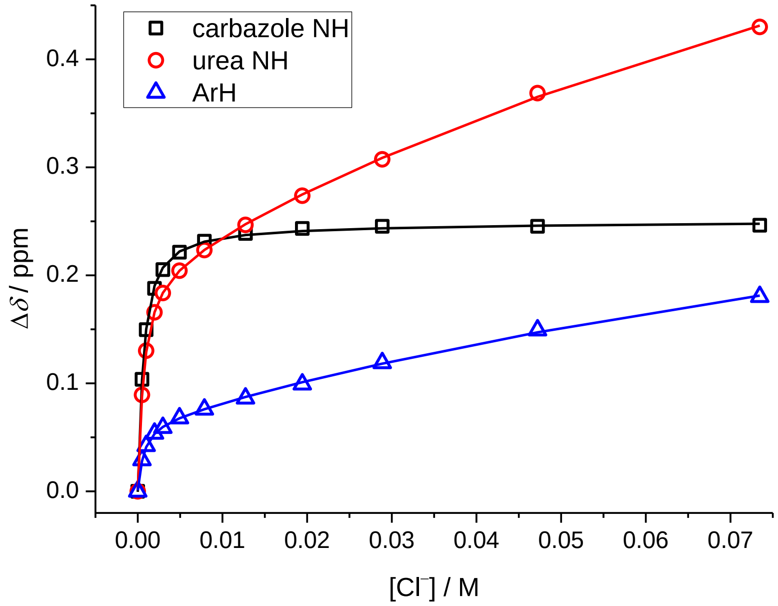


**Supplementary Figure 6** Global fitting of TBACl binding isotherms to a 1:2 (host:guest) binding model, giving $K_{11}$ = 2000 ± 100 $M^{-1}$ and $K_{12}$ = 5.7 ± 0.4 $M^{-1}$ from two titrations.

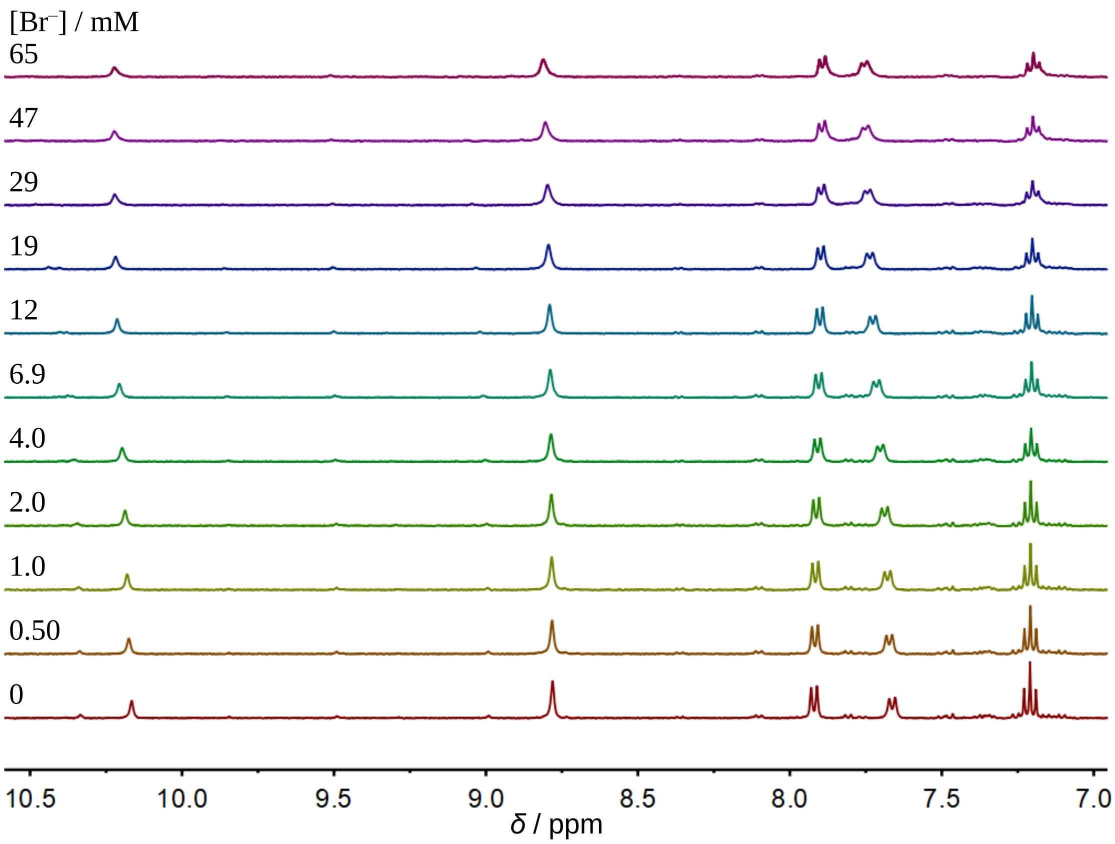


**Supplementary Figure 7** $^{1}$H NMR (400 MHz) titration of **1** (0.3 mM) with TBABr in DMSO-$d_6$/0.5% $H_2O$.

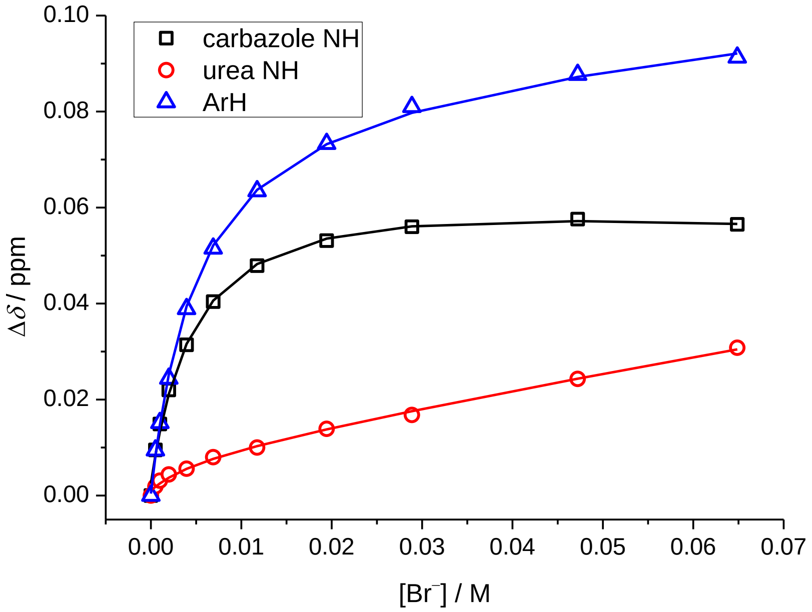


**Supplementary Figure 8** Global fitting of TBABr binding isotherms to a 1:2 (host:guest) binding model, giving $K_{11}$ = 200 ± 10 $M^{-1}$ and $K_{12}$ = 1.4 ± 0.3 $M^{-1}$ from two titrations.

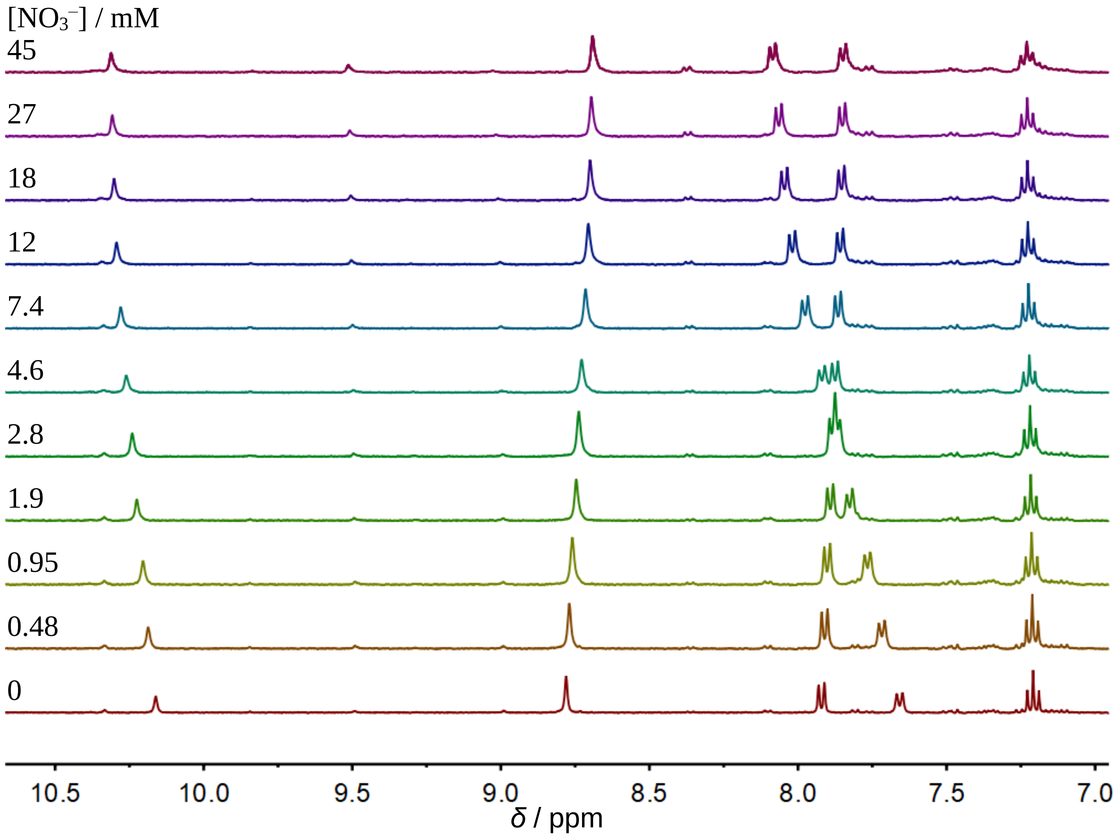


**Supplementary Figure 9** $^{1}$H NMR (400 MHz) titration of **1** (0.3 mM) with $TBANO_3$ in DMSO-$d_6$/0.5% $H_2O$. The upfield shift of the urea NH induced by $TBANO_3$ is attributed to receptor desolvation.

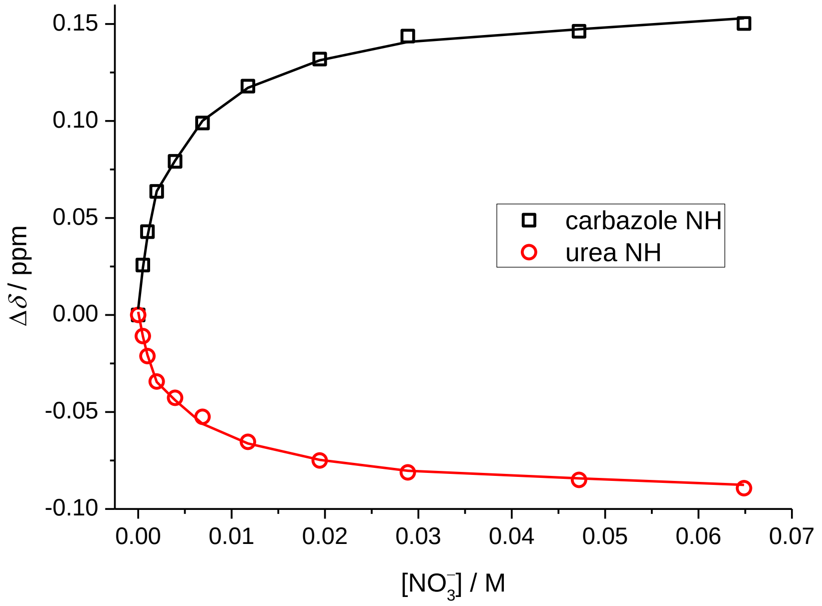


**Supplementary Figure 10** Global fitting of $TBANO_3$ binding isotherms to a 1:1 (host:guest) binding model, giving $K_{11}$ = 340 ± 10 $M^{-1}$ from two titrations.

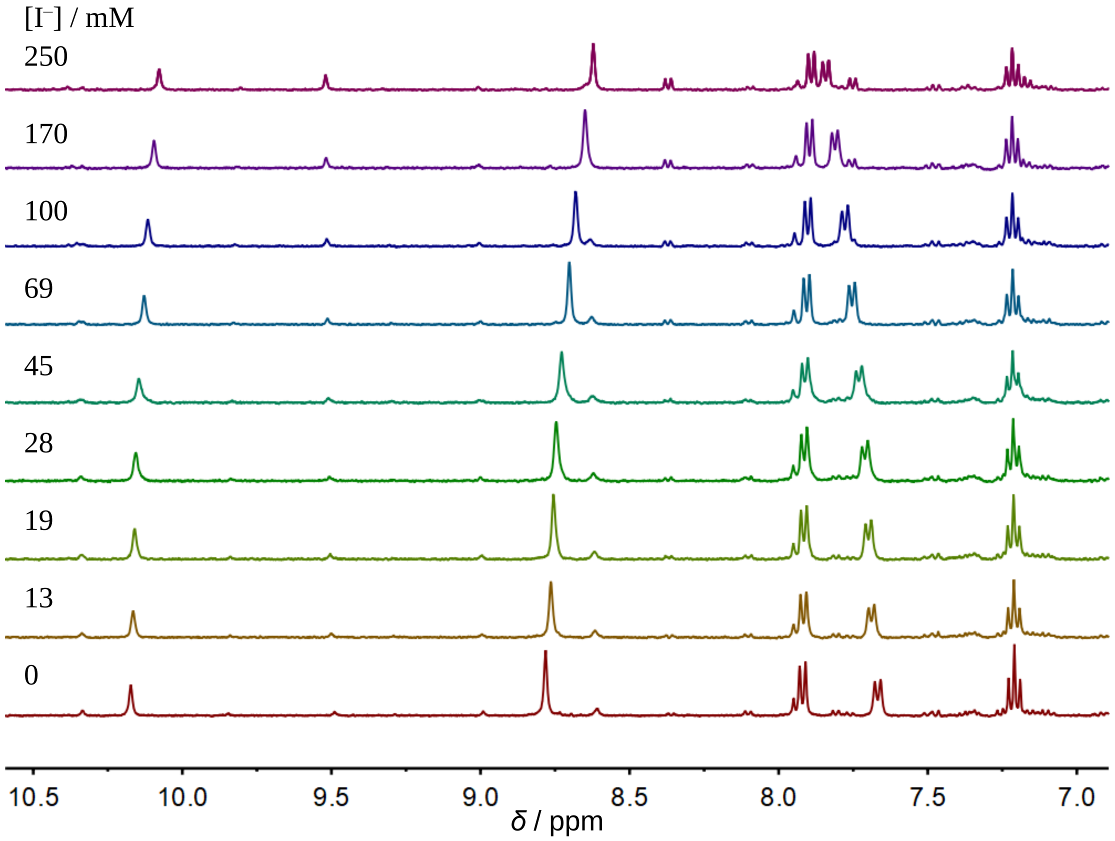


**Supplementary Figure 11** $^{1}H$ NMR (400 MHz) titration of **1** (0.3 mM) with TBAI in DMSO-$d_6$/0.5% $H_2O$. The upfield shift of both the carbazole NH and the urea NH induced by TBAI is attributed to receptor desolvation. Note that at high TBAI concentrations, the intensities of signals from the **1**-$SO_4^{2-}$ complex increased due to $SO_4^{2-}$ impurity in TBAI.

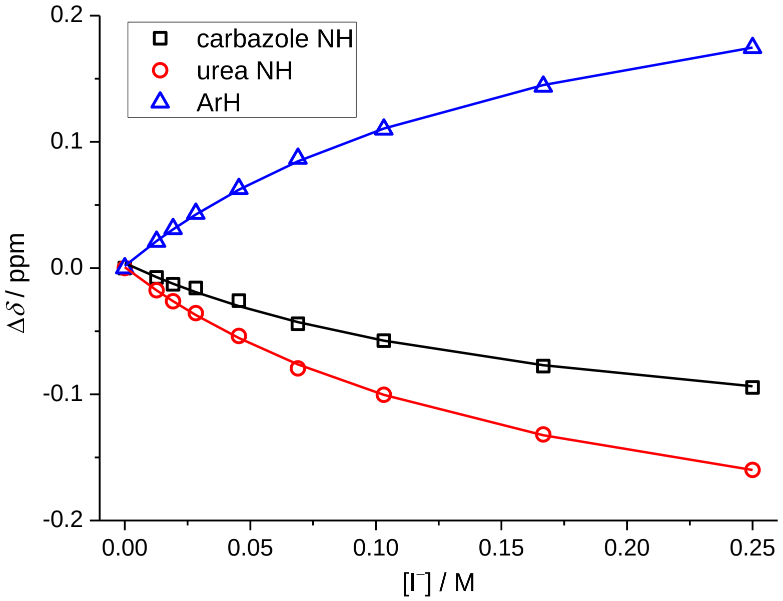


**Supplementary Figure 12** Global fitting of TBAI binding isotherms to a 1:1 (host:guest) binding model, giving $K_{11}$ = 6.1 ± 0.6 $M^{-1}$ from two titrations.

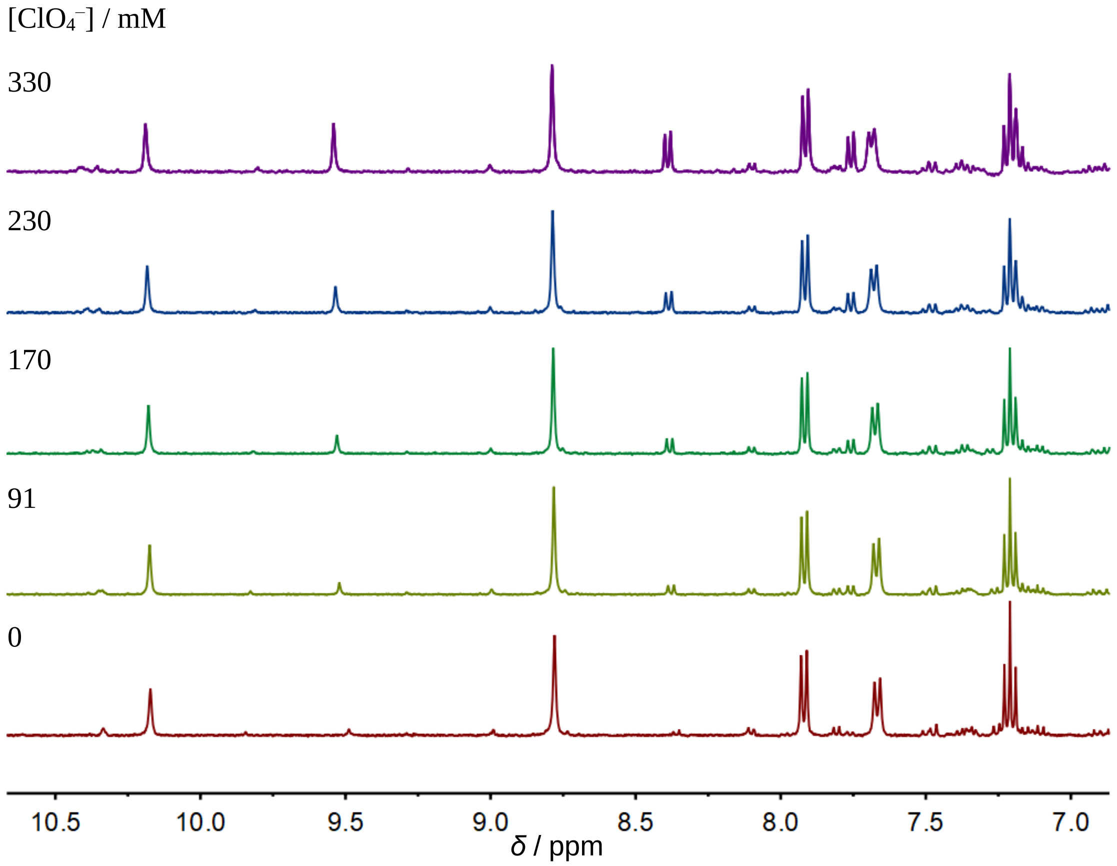


**Supplementary Figure 13** $^1$H NMR (400 MHz) titration of **1** (0.3 mM) with $TBAClO_4$ in DMSO-$d_6$/0.5% $H_2O$. Note that at high $TBAClO_4$ concentrations, the intensities of signals from the **1**-$SO_4^{2-}$ complex increased due to $SO_4^{2-}$ impurity in $TBAClO_4$. Assuming a $ClO_4^-$ affinity of 1 $M^{-1}$, a $ClO_4^-$ concentration of 330 mM corresponds to 25% saturation of anion binding, which would have shifted the urea NH upfield by at least 0.068 ppm due to receptor desolvation as estimated from the $I^-$ binding data. However, none of the peaks shifted significantly throughout the titration, and hence $ClO_4^-$ affinity is < 1 $M^{-1}$.

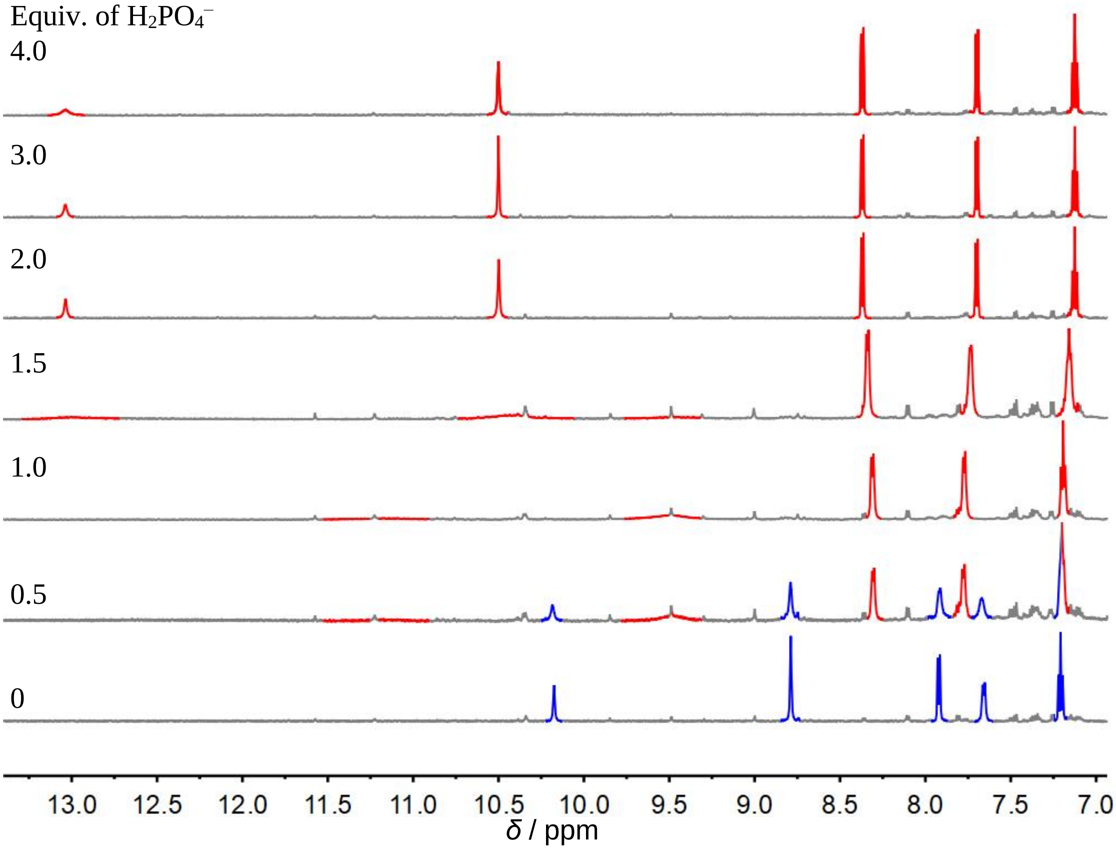


**Supplementary Figure 14** $^1$H NMR (600 MHz) titration of **1** (0.3 mM) with $TBAH_2PO_4$ in DMSO-$d_6$/0.5% $H_2O$. At 0.5 equiv. of $H_2PO_4^-$, a new set of signals can be observed (red) with similar intensities to those of free macrocycle **1** (blue), indicating the formation of **1**-$H_2PO_4^-$ complex in slow exchange with free macrocycle **1**. The free macrocycle signals completely disappeared at 1.0 equiv. of $H_2PO_4^-$, indicating a strong $H_2PO_4^-$ affinity of > $10^5$ $M^{-1}$. With > 1 equiv. of $H_2PO_4^-$, the signals shifted and saturated at 2 equiv., which are assigned to the **1**-$HPO_4^{2-}$ complex, formed via deprotonation of $H_2PO_4^-$ by a second $H_2PO_4^-$.[7] The **1**-$HPO_4^{2-}$ complex features more downfield shifted carbazole and urea NH resonances (13.0 and 10.5 ppm, respectively) than the **1**-$SO_4^{2-}$ complex (11.6 and 9.5 ppm, respectively), which is reasonable because $HPO_4^{2-}$ is more charge-dense than $SO_4^{2-}$.

## S4.2 Determination of sulfate affinity

Slow exchange signals were observed upon titration of the free macrocycle **1** with $TBA_2SO_4$ (Fig. 2a). Integration of the $TBA^+$ and the **1**-$SO_4^{2-}$ signals confirms the 1:1 stoichiometry. Free macrocycle **1** was completely converted to the $SO_4^{2-}$ complex at 1.0 equiv. of $TBA_2SO_4$, indicating a very strong $SO_4^{2-}$ affinity.

To determine the $SO_4^{2-}$ affinity of **1**, we used a $BaSO_4$ precipitation method taking advantage of a recently reported $K_{sp}$ value of $BaSO_4$ in DMSO.[8] To two solutions of **1**-$SO_4^{2-}$ (100 μM) in DMSO-$d_6$/0.5% $H_2O$ was added $BaCl_2$ (solids) to 50 mM and 100 mM, respectively. The solutions were sonicated until the $BaCl_2$ dissolved and then incubated for a week for $Ba^{2+}$ to extract $SO_4^{2-}$ from **1** and form $BaSO_4$. The $^1$H NMR of the two solutions were acquired, both of which showed separate sets of signals from **1**-$SO_4^{2-}$ and **1**-$Cl^-$ (here **1**-$Cl^-$ refers to a fast-exchanged mixture of **1**, **1**-1$Cl^-$ and **1**-2$Cl^-$), respectively (Supplementary Figure 15). By integration of the urea NH, we calculated the [**1**-$SO_4^{2-}$] / [**1**-$Cl^-$] ratio to be 100/7.1 and 100/15, for $BaCl_2$ at 50 mM and 100 mM, respectively. We have also prepared solutions of free **1** in the presence of 50 mM and 100 mM of $BaCl_2$ and verified the assignment of the new signals to **1**-$Cl^-$ that formed by competitive $Ba^{2+}$ binding to $SO_4^{2-}$ and subsequent $Cl^-$ binding to free **1**. The ionic strengths of the solutions are high and hence the reduced activity coefficients of $Ba^{2+}$ and $Cl^-$ need to be accounted for. By comparing the chemical shifts of the urea NH of **1** in the presence of $BaCl_2$ against the $Cl^-$ binding isotherm of **1** (Supplementary Figure 6), we determined the effective $Cl^-$ concentrations to be 49 mM and 79 mM, for $BaCl_2$ at 50 mM and 100 mM, respectively, which correspond to $Cl^-$ activity coefficients 0.49 and 0.40. These values are similar to the predictions of the extended Debye-Hückel equation previously used for electrolyte solutions in DMSO,[9] which gives $Cl^-$ activity coefficients of 0.53 and 0.47 for $BaCl_2$ at 50 mM ($I$ = 0.15 M) and 100 mM ($I$ = 0.3 M), respectively. The slight reduction of $Cl^-$ activity coefficients was modelled using an ion-pairing equilibrium between $Ba^{2+}$ and $Cl^-$, which gives a consistent apparent ion pairing equilibrium constant of 1.7 $M^{-1}$ for the formation of $[BaCl]^+$ ion pair under the two $BaCl_2$ concentration conditions. Using the extended Debye-Hückel equation[9] taking into account the apparent ion-pairing equilibrium constant ($K_{ip}$ = 1.7 $M^{-1}$), we calculated the $Ba^{2+}$ activity coefficients to be 0.027 and 0.059 for $BaCl_2$ at 50 mM and 100 mM, respectively. Finally, the binding constant of **1** for $SO_4^{2-}$ was calculated using the following equation:

$$K_a(\mathrm{SO_4^{2-}}) = \frac{[\mathbf{1}\cdot\mathrm{SO_4^{2-}}]\,\gamma(\mathrm{Ba^{2+}})\,[\mathrm{Ba^{2+}}]}{[\mathbf{1}\cdot\mathrm{Cl^-}]\,K_{sp}(\mathrm{BaSO_4})}\,[1{+}K_{11}(\mathrm{Cl^-})\,\gamma(\mathrm{Cl^-})\,[\mathrm{Cl^-}](1{+}\,K_{12}(\mathrm{Cl^-})\,\gamma(\mathrm{Cl^-})\,[\mathrm{Cl^-}])]$$

Supplementary Equation 1

Where $\frac{[\mathbf{1}\cdot\mathrm{SO_4^{2-}}]}{[\mathbf{1}\cdot\mathrm{Cl^-}]}$ is determined by $^1$H NMR integration to be 100/7.1 and 100/15, for $BaCl_2$ at 50 mM and 100 mM, respectively.
$\gamma(\mathrm{Ba^{2+}})$ is the $Ba^{2+}$ activity coefficient, calculated to be 0.027 and 0.059 for $BaCl_2$ at 50 mM and 100 mM, respectively.
$K_{sp}(\mathrm{BaSO_4})$ in DMSO was determined to be 5.5–7.7 × $10^{-10}$ $M^2$ by Fillingham et al.[8] Here we used the median value of 6.6 × $10^{-10}$ $M^2$.
$\gamma(\mathrm{Cl^-})$ is the $Cl^-$ activity coefficient, determined to be 0.49 and 0.40, for $BaCl_2$ at 50 mM and 100 mM, respectively.
$K_{11}(\mathrm{Cl^-})$ and $K_{12}(\mathrm{Cl^-})$ are 2000 $M^{-1}$ and 5.7 $M^{-1}$, respectively (Supplementary Figure 6).

Using Supplementary Equation 1, $K_a(\mathrm{SO_4^{2-}})$ was determined to be 7.9 × $10^9$ $M^{-1}$ and 6.4 × $10^9$ $M^{-1}$ with $BaCl_2$ used at 50 mM and 100 mM, respectively. We therefore report $K_a(\mathrm{SO_4^{2-}})$ to be (7.2 ± 1.1) × $10^9$ $M^{-1}$ in Table 1.

The derivation of Supplementary Equation 1 is shown as follows:

For $SO_4^{2-}$ binding to macrocycle **1**:

$$K_a(\mathrm{SO_4^{2-}}) = \frac{[\mathbf{1}\cdot\mathrm{SO_4^{2-}}]}{[\mathbf{1}]\,\gamma(\mathrm{SO_4^{2-}})[\mathrm{SO_4^{2-}}]} \qquad \text{Supplementary Equation 2}$$

For the $BaSO_4$ precipitation equilibrium:

$$\gamma(\mathrm{SO_4^{2-}})[\mathrm{SO_4^{2-}}] = \frac{K_{sp}(\mathrm{BaSO_4})}{\gamma(\mathrm{Ba^{2+}})[\mathrm{Ba^{2+}}]} \qquad \text{Supplementary Equation 3}$$

For $Cl^-$ binding to macrocycle **1**:

$$K_{11}(\mathrm{Cl^-}) = \frac{[\mathbf{1}\cdot 1\mathrm{Cl^-}]}{[\mathbf{1}]\gamma(\mathrm{Cl^-})[\mathrm{Cl^-}]} \qquad \text{Supplementary Equation 4}$$

$$K_{12}(\mathrm{Cl^-}) = \frac{[\mathbf{1}\cdot 2\mathrm{Cl^-}]}{[\mathbf{1}\cdot 1\mathrm{Cl^-}]\gamma(\mathrm{Cl^-})[\mathrm{Cl^-}]} \qquad \text{Supplementary Equation 5}$$

Combining Supplementary Equations 4 and 5 gives Supplementary Equation 6:

$$[\mathbf{1}] = \frac{[\mathbf{1}]+[\mathbf{1}\cdot 1\mathrm{Cl^-}]+[\mathbf{1}\cdot 2\mathrm{Cl^-}]}{[1+K_{11}(\mathrm{Cl^-})\,\gamma(\mathrm{Cl^-})\,[\mathrm{Cl^-}](1+K_{12}(\mathrm{Cl^-})\,\gamma(\mathrm{Cl^-})\,[\mathrm{Cl^-}])]} = \frac{[\mathbf{1}\cdot\mathrm{Cl^-}]}{[1+K_{11}(\mathrm{Cl^-})\,\gamma(\mathrm{Cl^-})\,[\mathrm{Cl^-}](1+K_{12}(\mathrm{Cl^-})\,\gamma(\mathrm{Cl^-})\,[\mathrm{Cl^-}])]}$$

Supplementary Equation 6

where $[\mathbf{1}\cdot\mathrm{Cl^-}]$ denotes the total concentration of a fast-exchanged mixture of **1**, $\mathbf{1}\cdot 1\mathrm{Cl^-}$ and $\mathbf{1}\cdot 2\mathrm{Cl^-}$.

The $\gamma(\mathrm{SO_4^{2-}})[\mathrm{SO_4^{2-}}]$ and $[\mathbf{1}]$ terms in Supplementary Equation 2 were substituted using Supplementary Equations 3 and 6, respectively, giving Supplementary Equation 1. Note that here we have neglected the activity coefficient terms of **1**-anion complexes, considering the charge delocalisation provided by macrocycle **1**.[10]

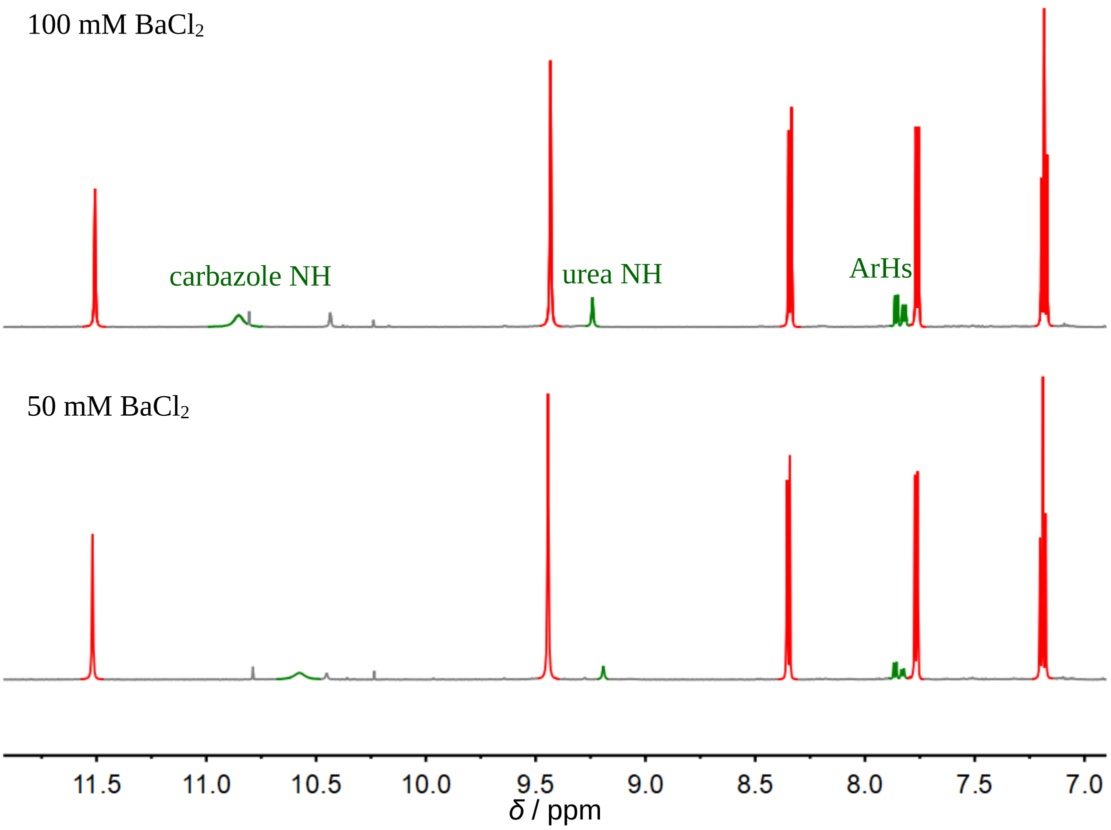


**Supplementary Figure 15** $^1$H NMR (600 MHz) spectra of **1**-$SO_4^{2-}$ (100 µM) in the presence of $BaCl_2$ at 50 mM (top) and 100 mM (bottom). The solutions were incubated for a week before data acquisition. The signals from **1**-$Cl^-$ (a fast-exchanged mixture of **1**, **1**-1$Cl^-$ and **1**-2$Cl^-$) are shown in green and assigned. One of the ArHs from **1**-$Cl^-$ is not visible due to overlapping with the **1**-$SO_4^{2-}$ signal. The **1**-$SO_4^{2-}$ signals are shown in red.

# S5. Fluorescence titrations in C12E8 micelles

## S5.1 Determination of sulfate and phosphate affinity

Titrations of **1** with $SO_4^{2-}$ and $H_2PO_4^-$ were performed in C12E8 micelles. 12.5 µL of a DMSO solution of **1**-$SO_4^{2-}$ (10 µM) was added to 2.5 mL of a micellar solution of C12E8 (2 mM) to a final macrocycle concentration of 50 nM. Because of the low concentration used, the **1**-$SO_4^{2-}$ complex completely dissociated into free macrocycle **1** (see Supplementary Figure 28 for evidence of its stability under the C12E8 micellar conditions). The solution in a 4 mL quartz cuvette was stirred and thermostated at 25 °C. Fluorescence spectra of **1** ($\lambda_{ex}$ = 265 nm) before and after adding increasing concentrations of $Na_2SO_4$ or $NaH_2PO_4$ were recorded. For $SO_4^{2-}$, the fluorescence intensity at 357 (corrected against dilution) was plotted against the concentration of $SO_4^{2-}$ and the data was fitted to a 1:1 binding models using OriginPro. For $H_2PO_4^-$, the fluorescence intensity values from 350 to 380 nm were globally fitted to the 1:2 (host:guest) model with dilution correction in BindFit. Note that here the use of the 1:2 model was to account for the slight dynamic fluorescence quenching observed with high concentrations of $H_2PO_4^-$ and the actual formation of the 1:2 complex should be negligible under the competitive aqueous conditions.

The $Na_2SO_4$ titration of **1** was also performed using 9:1 C12E8/POPC and 8:2 C12E8/POPC (mol:mol) mixed micelles to estimate competitive phosphate headgroup binding, following the abovementioned procedure except that POPC was used with the total C12E8 + POPC concentration kept constant at 2 mM. Note that the presence of 10% or 20% of POPC did not appear to impact the self-assembly of C12E8 micelles, as DLS measurement showed an identical hydrodynamic radius of 3.5 ± 0.1 nm for 100% C12E8, 9:1 C12E8/POPC and 8:2 C12E8/POPC micelles.

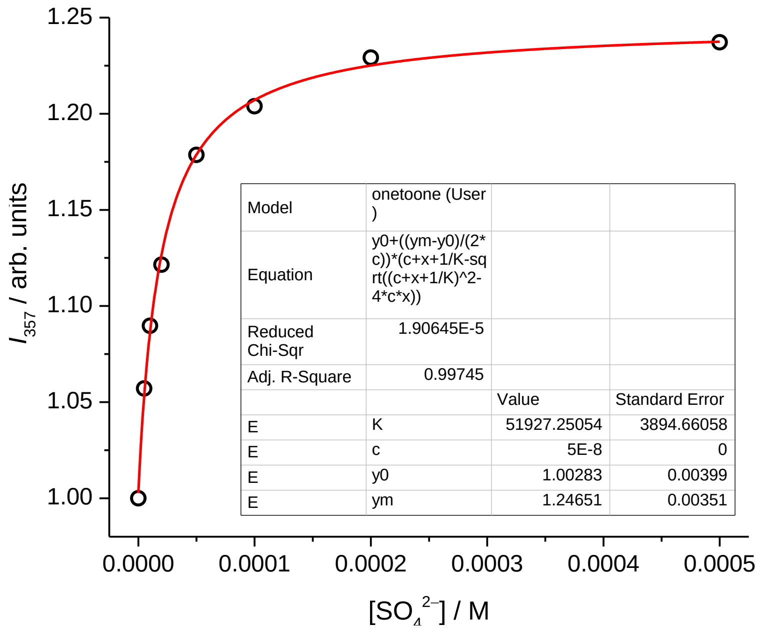


| Model | onetoone (User ) | | |
|---|---|---|---|
| Equation | y0+((ym-y0)/(2*c))*(c+x+1/K-sqrt((c+x+1/K)^2-4*c*x)) | | |
| Reduced Chi-Sqr | 1.90645E-5 | | |
| Adj. R-Square | 0.99745 | | |
| | | Value | Standard Error |
| E | K | 51927.25054 | 3894.66058 |
| E | c | 5E-8 | 0 |
| E | y0 | 1.00283 | 0.00399 |
| E | ym | 1.24651 | 0.00351 |

**Supplementary Figure 16** Fluorescence intensity of **1** (50 nM) at 357 nm with increasing concentrations of $Na_2SO_4$ in C12E8 (2 mM) micelles in $H_2O$. The data was fitted to a 1:1 binding model, giving a $SO_4^{2-}$ binding constant of 54000 ± 3000 $M^{-1}$ from two titrations.

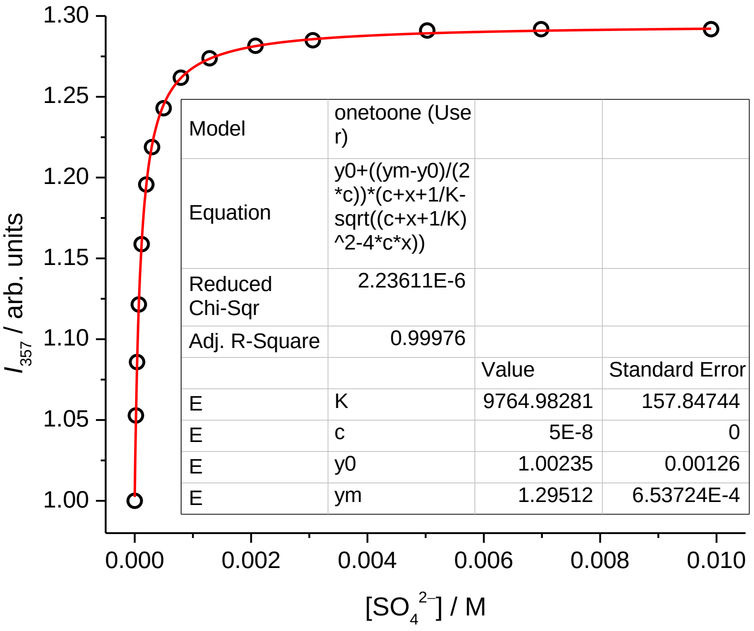


**Supplementary Figure 17** Fluorescence titration of **1** (50 nM) with $Na_2SO_4$ in 9:1 C12E8/POPC (total concentration 2 mM) micelles in $H_2O$. The data was fitted to a 1:1 binding model, giving a $SO_4^{2-}$ binding constant of 9900 ± 300 $M^{-1}$ from two titrations.

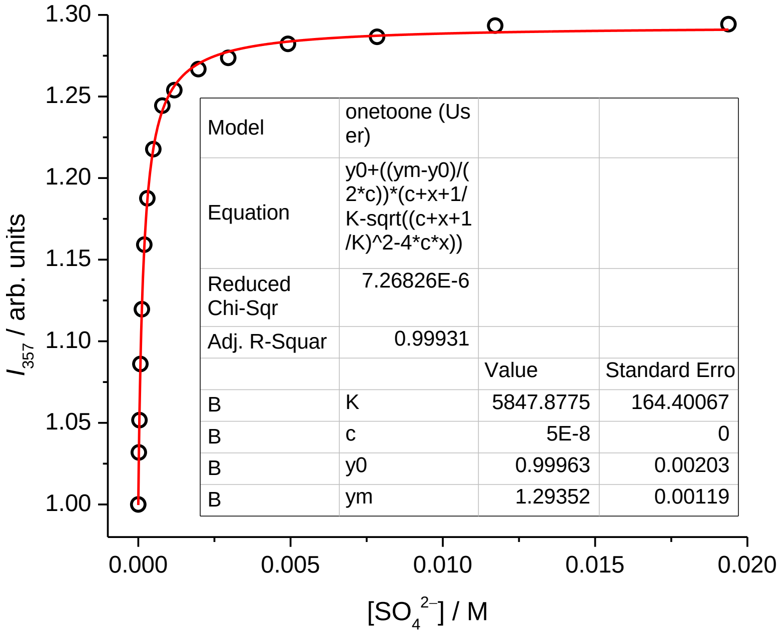


**Supplementary Figure 18** Fluorescence titration of **1** (50 nM) with $Na_2SO_4$ in 8:2 C12E8/POPC (total concentration 2 mM) micelles in $H_2O$. The data was fitted to a 1:1 binding model, giving a $SO_4^{2-}$ binding constant of 5400 ± 600 $M^{-1}$ from two titrations.

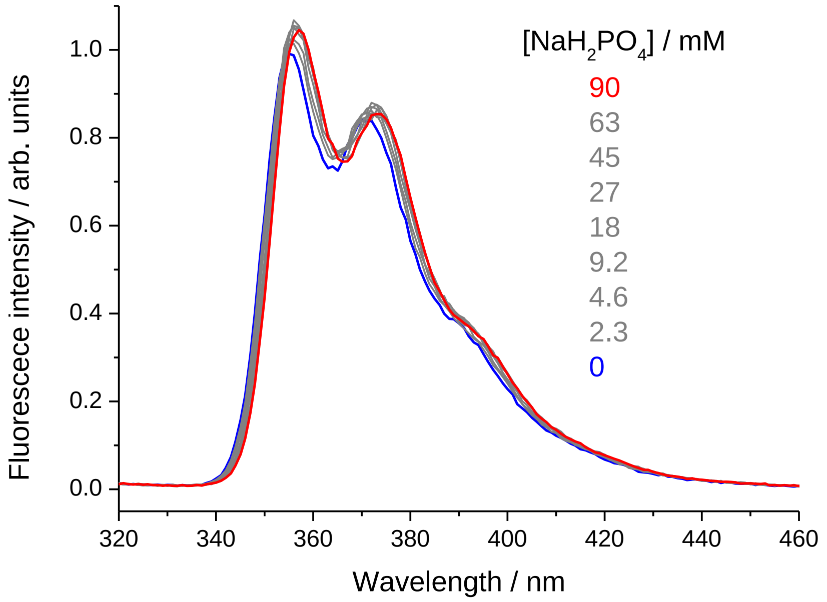


**Supplementary Figure 19** Fluorescence spectra of **1** (50 nM) with increasing concentrations of $NaH_2PO_4$ in C12E8 (2 mM) micelles in $H_2O$. $\lambda_{ex}$ = 265 nm.

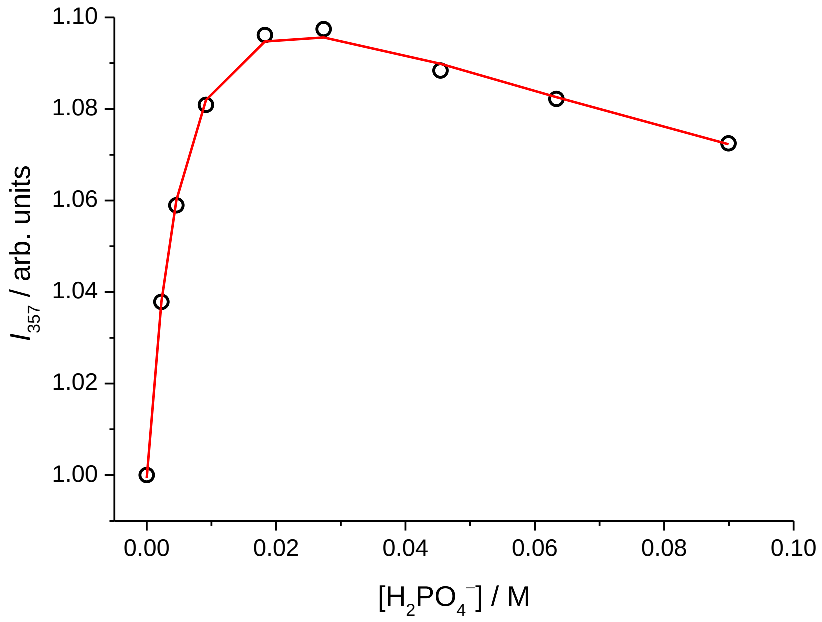


**Supplementary Figure 20** Fluorescence intensity of **1** (50 nM) at 357 nm with increasing concentrations of $NaH_2PO_4$ in C12E8 (2 mM) micelles in $H_2O$. Global fitting of 350–380 nm to a 1:2 binding model gave a $H_2PO_4^-$ binding constant ($K_{11}$) of 140 ± 20 $M^{-1}$ from two titrations. For full data fitting, see: http://app.supramolecular.org/bindfit/view/5d5fed84-a3bd-4c11-b21a-355b29ac5c91

## S5.2 Determination of affinities of other anions

Supplementary Figure 21 shows fluorescence enhancement responses of **1** to $SO_4^{2-}$ (red) and $H_2PO_4^-$ (orange), which has allowed for binding constant determination by fluorescence titrations (Supplementary Figures 16–20). The fluorescence response of **1** to $Cl^-$, however, was too weak for binding constant determination (Supplementary Figure 21 green). $Br^-$ (brown), $NO_3^-$ (blue), $I^-$ (purple) and $ClO_4^-$ (pink) induced fluorescence responses, which in part originated from a dynamic quenching mechanism, so the binding constants calculated from the fluorescence titration data would be incorrect in those cases.

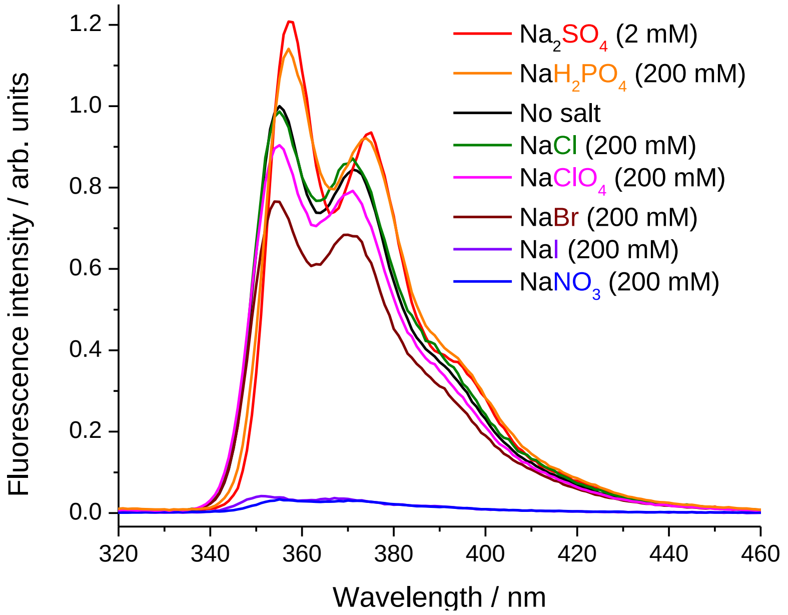


**Supplementary Figure 21** Fluorescence spectra of **1** (50 nM) in the absence and presence of salts in C12E8 (2 mM) micelles in $H_2O$. $\lambda_{ex}$ = 265 nm.

To determine the affinities of **1** for anions other than $SO_4^{2-}$ and $H_2PO_4^-$, we performed the $Na_2SO_4$ titration in the presence of 200 mM of the tested anion as a competitive binder. Instead of collecting the full fluorescence spectra, we only recorded the fluorescence intensities at 357 nm and 365 nm in competition titrations to minimise photobleaching. The binding constant of the competitive binder could be calculated by a competition binding scheme using the $SO_4^{2-}$ affinities in the absence and presence of the competitive binder. However, surface potential effects need to be corrected because anions can adsorb to non-ionic micelles[11] resulting in a negative surface potential which depletes $SO_4^{2-}$ ions from the solution by a Boltzmann factor.

To correct for surface potential effects, we determined zeta potential of C12E8 (2 mM) micelles in the presence of 200 mM of salts by electrophoretic mobility measurements at 25 °C, using DTS1070 folded capillary cells. Note that in the case of NaI, it is necessary to reduce the applied voltage to minimise the electrolysis of $I^-$, which produces more lipophilic $I_3^-$ and enhances the electrophoretic mobility of micelles. We also determined the size of the micelles in the presence of salts at 200 mM and found the hydrodynamic diameter to be 3.7 ± 0.3 nm (similar to the value without salts) regardless of the identity of the salt. The zeta potential ($\zeta$) was calculated using Henry's equation:

$$\zeta = \frac{3\mu_e\eta}{2\varepsilon_0\varepsilon_r f(\kappa a)} \qquad \text{Supplementary Equation 7}$$

where $\mu_e$ is the electrophoretic mobility, $\eta$ is the viscosity of the water, $\varepsilon_0$ is the vacuum permittivity, $\varepsilon_r$ is the relative permittivity of water, and $f(\kappa a)$ is Henry's function (calculated to be 1.16 using the Malvern Zetasizer software for C12E8 micelles with a hydrodynamic radius of 3.7 nm).

The zeta potentials were then converted to surface potentials ($\Phi_0$) using the following relationship from the Gouy-Chapman model:[12]

$$\kappa x = \ln \left[\frac{(e^{\frac{ZF\Phi_x}{2RT}}+1)(e^{\frac{ZF\Phi_0}{2RT}}-1)}{(e^{\frac{ZF\Phi_x}{2RT}}-1)(e^{\frac{ZF\Phi_0}{2RT}}+1)}\right] \qquad \text{Supplementary Equation 8}$$

where $\Phi_x$ is the zeta potential ($\zeta$), $Z = -1$, $F$ is the Faraday constant, $R$ is the gas constant, $\kappa$ is the inverse of Debye length, and $x$ is the distance of the shear plane from the surface (assumed to be 0.6 Å as determined by Yang et al. for similarly-sized micelles at $I$ = 0.2 M[13]).

The zeta potential and surface potential values are summarised in Supplementary Table 2.

**Supplementary Table 2** Zeta potential ($\zeta$) and calculated surface potential ($\Phi_0$) of C12E8 (2 mM) micelles in the presence of 200 mM of salts in $H_2O$. The values are shown as average ± SD from at least three measurements.

| Salt (200 mM) | $\zeta$ / mV | $\Phi_0$ / mV |
|---|---|---|
| $NaH_2PO_4$ | –18.1 ± 1.6 | –19.80 ± 1.8 |
| NaCl | –5.5 ± 1.0 | –6.0 ± 1.0 |
| NaBr | –11.0 ± 1.2 | –12.0 ± 1.3 |
| $NaNO_3$ | –5.6 ± 1.2 | –6.1 ± 1.3 |
| NaI | –14.0 ± 2.0 | –15.3 ± 2.2 |
| $NaClO_4$ | –13.3 ± 0.9 | –14.5 ± 1.0 |

From the surface potential ($\Phi_0$) values, Boltzmann factors can be calculated for monovalent (*i.e.*, the competing anion) and divalent (*i.e.*, $SO_4^{2-}$) anions. Given the sensitivity of the $SO_4^{2-}$ activity coefficient to the ionic strength of the solution, it is preferable to perform the competition experiments at a fixed ionic strength. We calculated the $SO_4^{2-}$ binding constant at $I$ = 0.2 M, using the following equation:

$$K_a(\mathrm{SO_4^{2-}})^{I=0.2\ \mathrm{M}} = \frac{K_{app}(\mathrm{SO_4^{2-}})^{\mathrm{NaH_2PO_4}}}{e^{\frac{-2F\Phi_0}{RT}}}\left(K(\mathrm{H_2PO_4^-})[\mathrm{NaH_2PO_4}]e^{\frac{-F\Phi_0}{RT}} + 1\right)$$

Supplementary Equation 9

where $K_a(\mathrm{SO_4^{2-}})^{I=0.2\ \mathrm{M}}$ is the $SO_4^{2-}$ binding constant at $I$ = 0.2 M, $K_{app}(\mathrm{SO_4^{2-}})^{\mathrm{NaH_2PO_4}}$ is the apparent binding constant of $SO_4^{2-}$ in the presence of 200 mM of $NaH_2PO_4$ (1400 $M^{-1}$, Supplementary Figure 22), $\Phi_0$ is the surface potential of C12E8 micelles in the presence of 200 mM of $NaH_2PO_4$ (–19.80 mV), [$NaH_2PO_4$] = 200 mM, and $K(\mathrm{H_2PO_4^-})$ is the $H_2PO_4^-$ affinity of **1** (140 $M^{-1}$, Supplementary Figure 20). Here we assumed the $H_2PO_4^-$ affinities of **1** at $I$ = 0 and $I$ = 0.2 to be identical as an approximation, given the monovalent nature of $H_2PO_4^-$. $K_a(\mathrm{SO_4^{2-}})^{I=0.2}$ is calculated to be 94000 ± 3000 $M^{-1}$, which is similar to $K_a(SO_4^{2-})$ at $I$ = 0 (54000 $M^{-1}$, Supplementary Figure 16). The slight enhancement of $K_a(\mathrm{SO_4^{2-}})$ at $I$ = 0.2 M might result from $Na^+$ ions interacting with the carbonyl groups of **1** thus polarising the urea NH binding sites.

Finally, the binding constant of **1** for a competing anion at $I$ = 0.2 is calculated using the following equation:

$$K_a(\mathrm{anion})^{I=0.2\ \mathrm{M}} = \frac{\frac{K_a(\mathrm{SO_4^{2-}})^{I=0.2\ \mathrm{M}}}{K_{app}(\mathrm{SO_4^{2-}})^{\mathrm{anion}}}e^{\frac{-2F\Phi_0}{RT}} - 1}{[\mathrm{anion}]e^{\frac{-F\Phi_0}{RT}}} \qquad \text{Supplementary Equation 10}$$

where $K_a(\mathrm{anion})^{I=0.2\ \mathrm{M}}$ is the affinity of **1** for a competing anion at $I$ = 0.2 M, $K_{app}(\mathrm{SO_4^{2-}})^{\mathrm{anion}}$ is the apparent binding constant of $SO_4^{2-}$ in the presence of the competing anion, $\Phi_0$ is the surface

potential in the presence of the competing anion (Supplementary Table 2), and [anion] is the concentration of the competing anion.

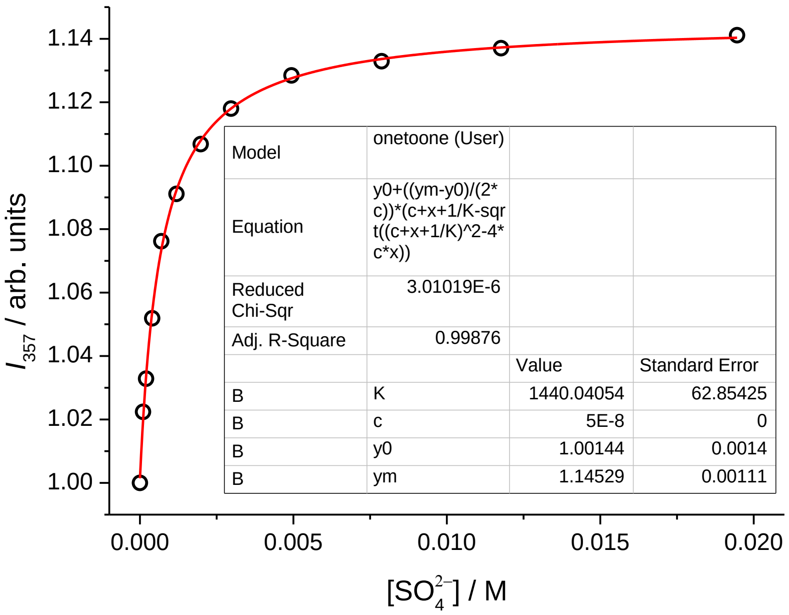


**Supplementary Figure 22** Competition fluorescence titration of **1** (50 nM) with $Na_2SO_4$ in the presence of $NaH_2PO_4$ (200 mM) in C12E8 (2 mM) micelles in $H_2O$. The data was fitted to a 1:1 binding model, giving an apparent $SO_4^{2-}$ binding constant of 1400 ± 100 $M^{-1}$ from two titrations. This value was used to calculate $K_a(SO_4^{2-})^{I=0.2}$ using Supplementary Equation 9.

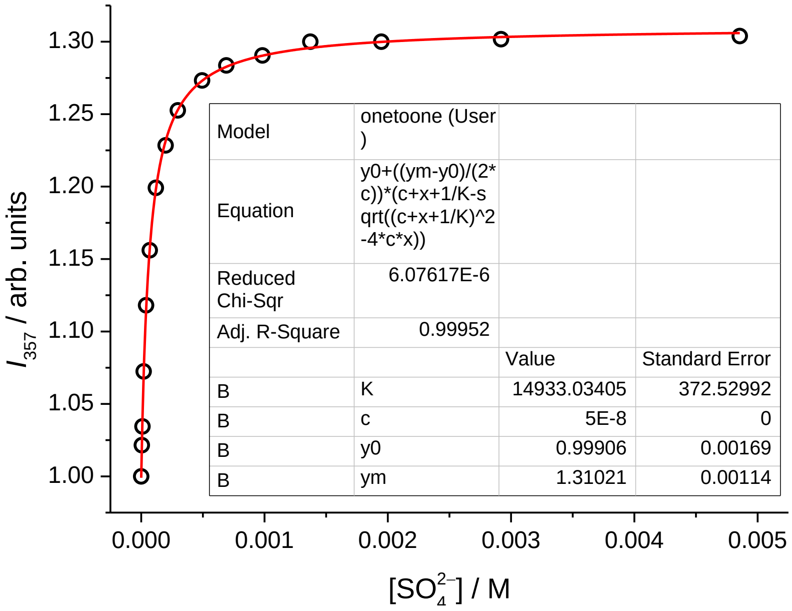


**Supplementary Figure 23** Competition fluorescence titration of **1** (50 nM) with $Na_2SO_4$ in the presence of NaCl (200 mM) in C12E8 (2 mM) micelles in $H_2O$. The data was fitted to a 1:1 binding model, giving an apparent $SO_4^{2-}$ binding constant of 14000 ± 1000 $M^{-1}$ from two titrations. From this value, the $Cl^-$ binding constant of **1** was calculated to be 19 ± 1 $M^{-1}$ using Supplementary Equation 10.

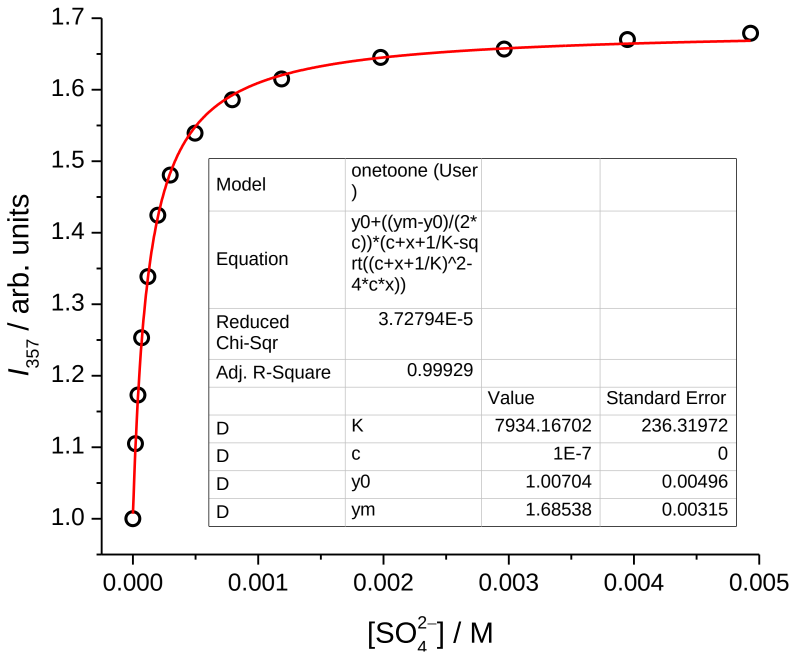


**Supplementary Figure 24** Competition fluorescence titration of **1** (50 nM) with $Na_2SO_4$ in the presence of NaBr (200 mM) in C12E8 (2 mM) micelles in $H_2O$. The data was fitted to a 1:1 binding model, giving an apparent $SO_4^{2-}$ binding constant of 7900 ± 100 $M^{-1}$ from two titrations. From this value, the $Br^-$ binding constant of **1** was calculated to be 29 ± 1 $M^{-1}$ using Supplementary Equation 10.

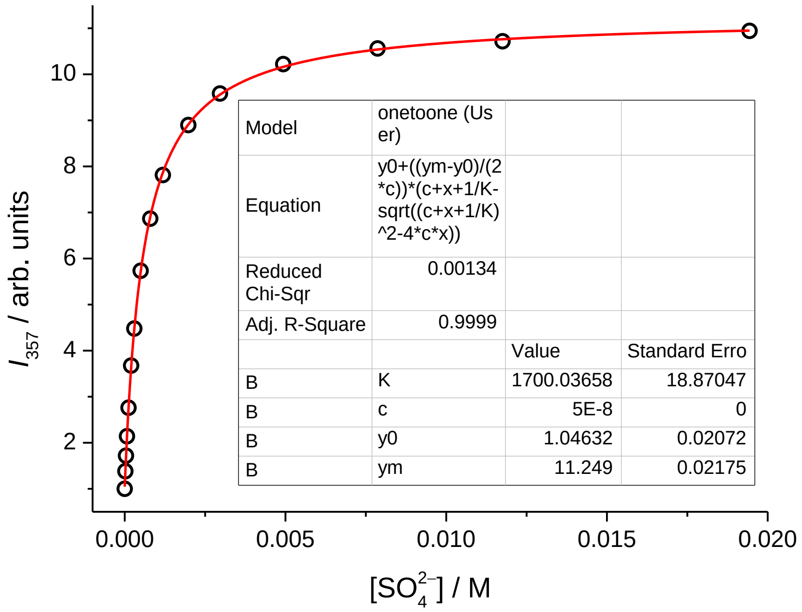


**Supplementary Figure 25** Competition fluorescence titration of **1** (50 nM) with $Na_2SO_4$ in the presence of $NaNO_3$ (200 mM) in C12E8 (2 mM) micelles in $H_2O$. The data was fitted to a 1:1 binding model, giving an apparent $SO_4^{2-}$ binding constant of 1700 ± 100 $M^{-1}$ from two titrations. From this value, the $NO_3^-$ binding constant of **1** was calculated to be 210 ± 10 $M^{-1}$ using Supplementary Equation 10.

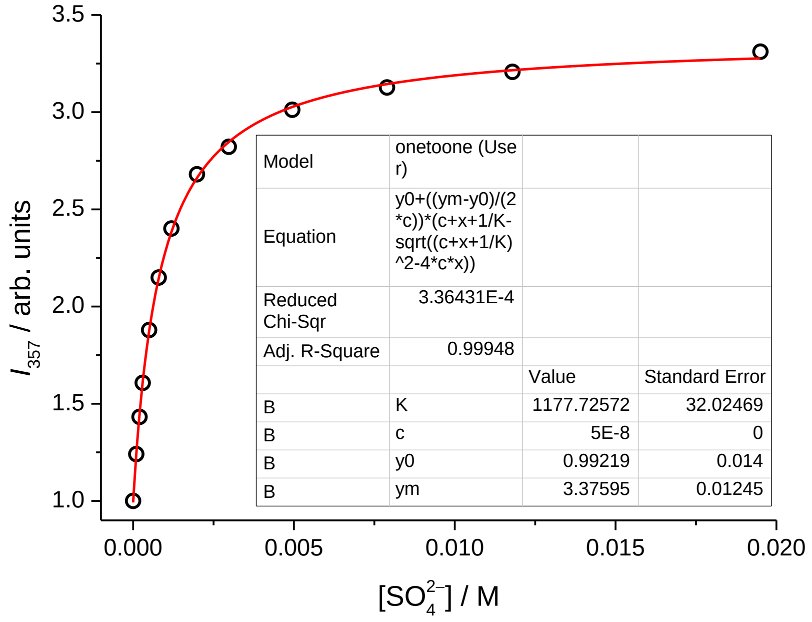


**Supplementary Figure 26** Competition fluorescence titration of **1** (50 nM) with $Na_2SO_4$ in the presence of NaI (200 mM) in C12E8 (2 mM) micelles in $H_2O$. The data was fitted to a 1:1 binding model, giving an apparent $SO_4^{2-}$ binding constant of 1200 ± 100 $M^{-1}$ from two titrations. From this value, the $I^-$ binding constant of **1** was calculated to be 200 ± 10 $M^{-1}$ using Supplementary Equation 10.

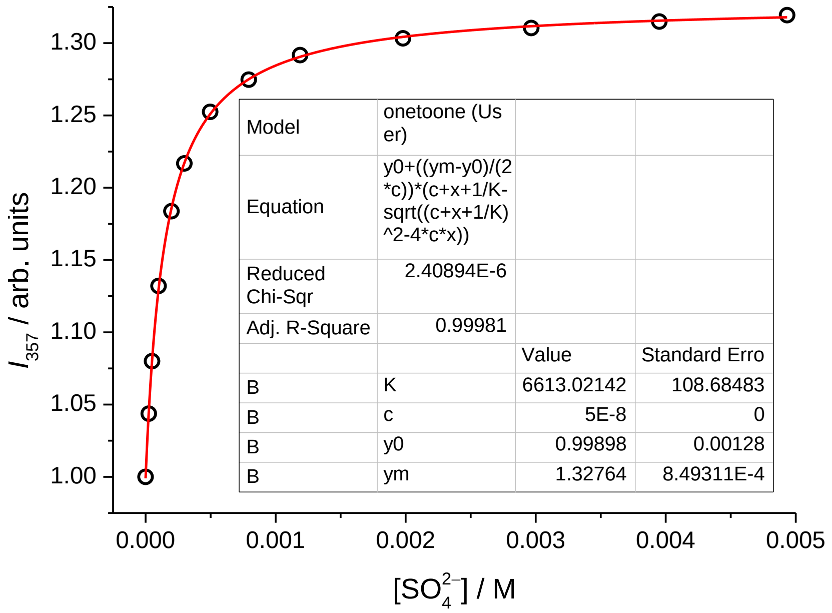


**Supplementary Figure 27** Competition fluorescence titration of **1** (50 nM) with $Na_2SO_4$ in the presence of $NaClO_4$ (200 mM) in C12E8 (2 mM) micelles in $H_2O$. The data was fitted to a 1:1 binding model, giving an apparent $SO_4^{2-}$ binding constant of 6500 ± 200 $M^{-1}$ from two titrations. From this value, the $I^-$ binding constant of **1** was calculated to be 32 ± 1 $M^{-1}$ using Supplementary Equation 10.

# S6. $^{1}$H NMR evidence of anion binding in C12E8 micelles

## S6.1 Evidence of anion binding

We found that good-to-reasonable quality $^{1}$H NMR spectra of anion complexes of macrocycle **1** (at µM concentrations) in C12E8 micelles could be obtained using a 600 MHz NMR spectrometer equipped with a cryoprobe, providing direct evidence of anion binding in C12E8 micelles. For solution preparation, 5 µL of a DMSO-*d*$_6$ solution of **1**-$SO_4^{2-}$ (0.5 mM) was added to a 495 µL solution of C12E8 (50 mM) in 9:1 (v:v) $H_2O/D_2O$, to a final macrocycle concentration of 5.0 µM. Solutions containing 2 mM of $Na_2SO_4$ or 200 mM of $NaH_2PO_4$, NaCl, NaBr, $NaNO_3$, NaI, or $NaClO_4$ were also prepared. The zgesgppe pulse programme was used to suppress the water signal. The spectra were referenced to the DMSO-*d*$_5$ signal (set as 2.71 ppm).

$^{1}$H NMR spectrum of **1**-$SO_4^{2-}$ (5.0 µM) without any added salt (Supplementary Figure 28 bottom) only showed signals from the **1**-$SO_4^{2-}$ (which are similar to its spectrum in DMSO-*d*$_6$, Supplementary Figure 1, featuring highly downfield shifted carbazole and urea NHs). The integration of **1**-$SO_4^{2-}$ signals increased by 1.6-fold upon addition of $Na_2SO_4$ (2 mM). This indicates that in the absence of any added salt, 62% of **1** existed as the free macrocycle when **1**-$SO_4^{2-}$ was used at 5.0 µM, but the free macrocycle signals are not visible by solution $^{1}$H NMR due to aggregation of the free macrocycle leading to signal broadening. The clean $^{1}$H NMR of **1**-$SO_4^{2-}$ after the addition of $Na_2SO_4$ (2 mM, Supplementary Figure 28 top) confirmed the stability of the free macrocycle **1** under aqueous conditions.

In the presence of 200 mM of $NaH_2PO_4$, $NaNO_3$, or NaI, the signals of the **1**-$SO_4^{2-}$ complex disappeared, with the emergence of new sets of signals assigned to the respective anion complexes of **1** (Supplementary Figure 29a–c). By contrast, with 200 mM of NaCl, NaBr, or $NaClO_4$, the **1**-$SO_4^{2-}$ complex coexisted with the respective anion complexes (Supplementary Figure 29d–f). This is consistent with fluorescence binding data (Table 1) showing low affinities of $Cl^-$, $Br^-$ and $ClO_4^-$ ($K_a$ < 32 $M^{-1}$) and relatively high affinities of $H_2PO_4^-$, $NO_3^-$ and $I^-$ ($K_a$ > 140 $M^{-1}$) which were able to fully displace $SO_4^{2-}$ from **1** in C12E8 micelles.

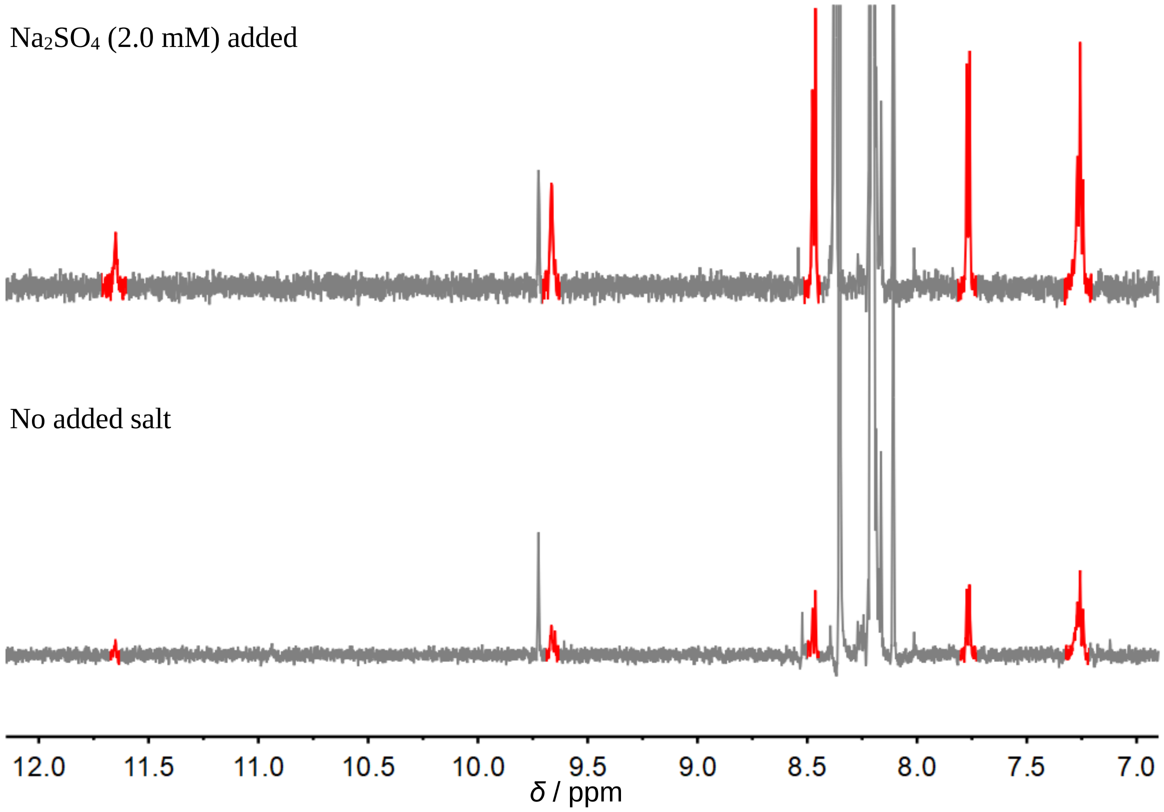


**Supplementary Figure 28** $^{1}$H NMR (600 MHz) of **1**-$SO_4^{2-}$ (5.0 µM) in C12E8 (50 mM) micelles in 9:1 $H_2O/D_2O$ in the absence (bottom) and presence of $Na_2SO_4$ (2 mM). The signals from **1**-$SO_4^{2-}$ are shown in red. The remaining signals are from C12E8 or impurities in the solvent/C12E8.

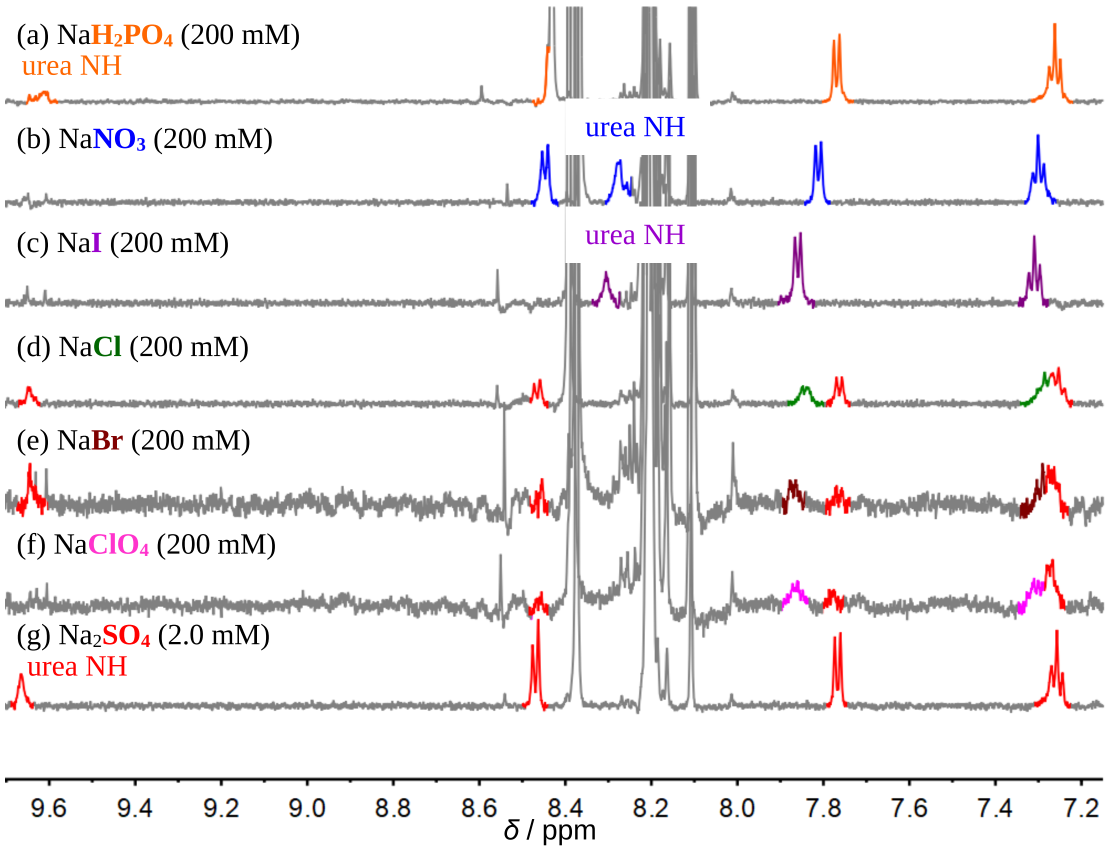


**Supplementary Figure 29** $^1$H NMR (600 MHz) of **1**-$SO_4^{2-}$ (5.0 µM) in C12E8 (50 mM) micelles in 9:1 $H_2O/D_2O$ in the presence of 200 mM of $NaH_2PO_4$, $NaNO_3$, NaI, NaCl, NaBr, or $NaClO_4$, or 2 mM of $Na_2SO_4$. The signals from the respective anion complexes are colour coded according to the labels on the left. Some of the urea NH signals are assigned. See Supplementary Figure 28 for the carbazole NH signal of **1**-$SO_4^{2-}$ at 11.7 ppm. Note that some signals are invisible due to broadening or overlapping with signals from C12E8. The spectra for NaBr and $NaClO_4$ were scaled up due to low signal intensities, likely indicating $Br^-$ and $ClO_4^-$ enhancing aggregation of **1** under the current conditions. In fluorescence titrations, we used 100-fold diluted macrocycle **1** to minimise aggregation of **1**. The remaining signals are from C12E8 or impurities in the solvent/C12E8.

## S6.2 Evidence of lipid headgroup binding

We also performed $^{1}$H NMR on **1**-$SO_4^{2-}$ (5.0 μM) in 9:1 C12E8/POPC and 8:2 C12E8/POPC mixed micelles, to obtain evidence of **1** binding to the lipid phosphate headgroup under aqueous biphasic conditions. The mixed micelles were preparing by dissolving POPC in C12E8 micellar solutions in 9:1 $H_2O/D_2O$, with sonication to assist dissolution of POPC solids. The total POPC + C12E8 concentration was 50 mM. **1**-$SO_4^{2-}$ was then added to the micellar solutions in a small volume of DMSO-$d_6$ and $^{1}$H NMR was recorded following the procedure in S7.1. The $^{1}$H NMR spectra of the resultant solutions show a progressive transformation of **1**-$SO_4^{2-}$ into the **1**-POPC complex (Supplementary Figure 30), supporting that **1** can bind to the lipid headgroup under aqueous conditions, although the microenvironments are different in the mixed micelles and in lipid bilayers.

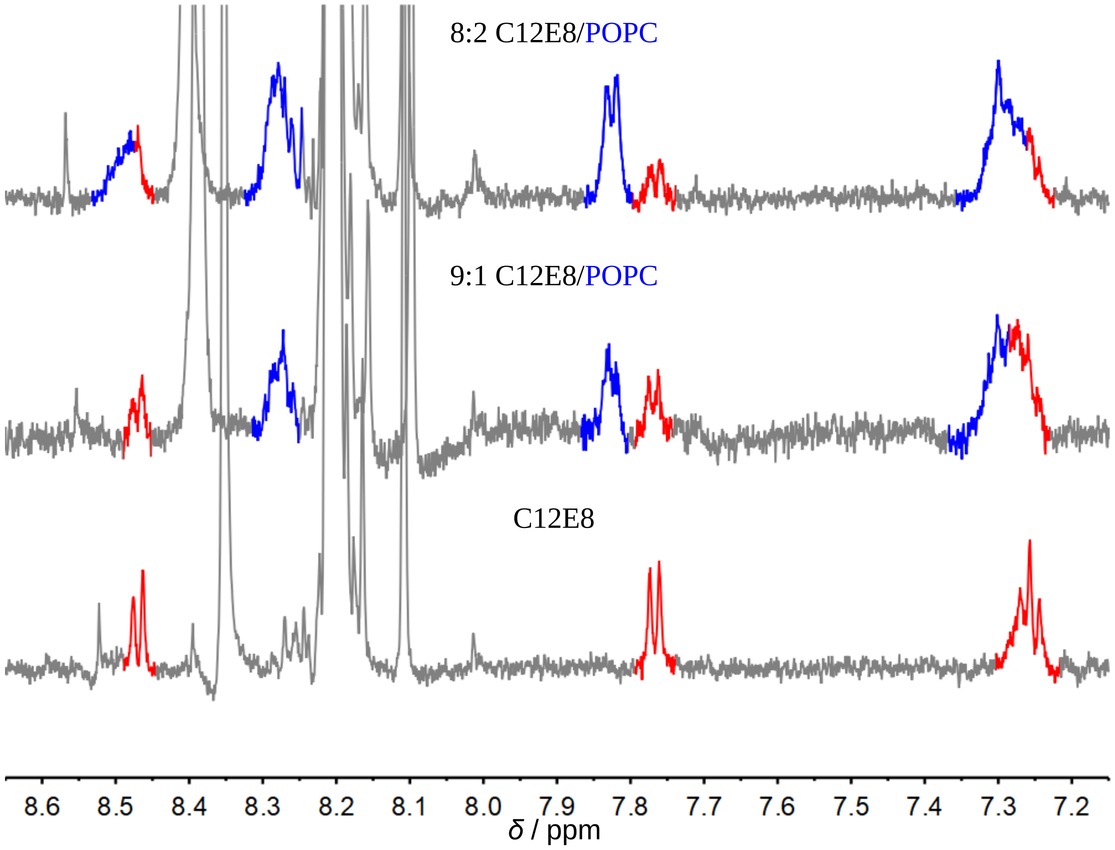


**Supplementary Figure 30** $^{1}$H NMR (600 MHz) of **1**-$SO_4^{2-}$ (5.0 μM) in C12E8 (bottom), 9:1 C12E8/POPC (middle), and 8:2 C12E8/POPC (top) micelles in 9:1 $H_2O/D_2O$. [POPC] + [C12E8] = 50 mM. The signals from **1**-$SO_4^{2-}$ and **1**-POPC complexes are shown in red and blue, respectively. The remaining signals are from C12E8 or impurities in the solvent/C12E8.

# S7. Fluorescence titrations in POPC vesicles

## S7.1 Determination of sulfate affinity

POPC LUVs with a diameter of 100 nm were prepared as follows. A lipid film was formed by evaporating 2 mL of a chloroform solution of POPC (1 g in 35 mL) in a 10 mL round bottom flask. After being dried under vacuum for at least 6 hours, the lipids were hydrated with 2 mL of Milli-Q water to form vesicles, vortexed, and then subject to 9 freeze-thaw cycles. The vesicles were extruded 39 times through a 100 nm polycarbonate membrane. The concentration of POPC was calculated based on the weight difference of the round bottom flask before POPC addition and after drying. For each titration, the vesicles were diluted to a POPC concentration of 0.2 mM and a final volume of 2.5 mL in water. The suspension in a 4 mL quartz cuvette was stirred and thermostated at 25 °C. The emission spectrum ($\lambda_{ex}$ = 265 nm, $\lambda_{em}$ = 300–500 nm) of POPC vesicles without **1** was collected for baseline subtraction. 12.5 µL of a DMSO solution of **1**-$SO_4^{2-}$ (10 µM) was added, to a final macrocycle concentration of 50 nM. Because of the low concentration used, the **1**-$SO_4^{2-}$ complex completely dissociated into free macrocycle **1**. Fluorescence emission spectra ($\lambda_{ex}$ = 265 nm, $\lambda_{em}$ = 300–500 nm) of **1** before and after adding increasing concentrations of $Na_2SO_4$ were recorded. The fluorescence intensity at 358 (corrected against vesicle scattering and dilution) was plotted against the concentration of $SO_4^{2-}$ and the data was fitted to the 1:1 binding model using OriginPro.

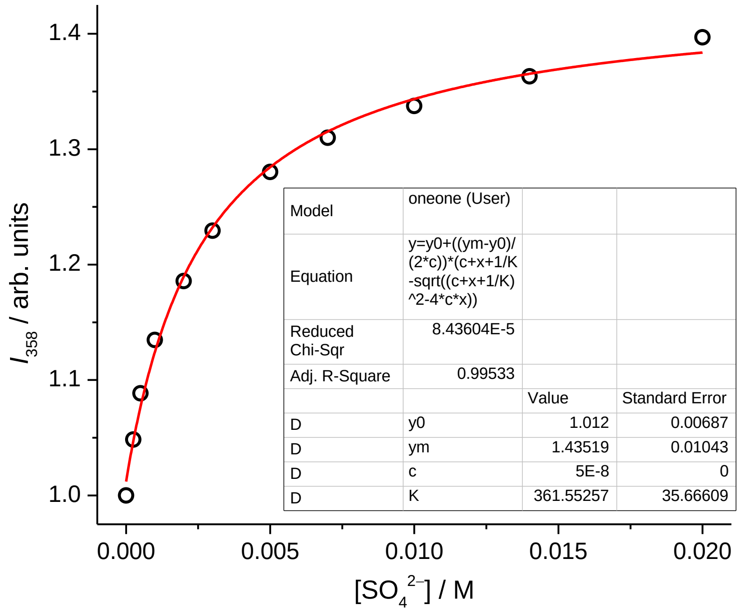


**Supplementary Figure 31** Fluorescence intensity of **1** (50 nM) at 358 nm with increasing concentrations of $Na_2SO_4$ in POPC (0.2 mM) vesicles in $H_2O$. The data was fitted to a 1:1 binding model, giving a $SO_4^{2-}$ binding constant of 350 ± 20 $M^{-1}$ from two titrations.

## S7.2 Determination of affinities of other anions

Similar to the observation in C12E8 micelles (Supplementary Figure 21), anions other than $SO_4^{2-}$ induced negligible responses or fluorescence quenching responses (Supplementary Figure 32), necessitating the use of competitive experiments to determine their affinities for **1**. Although in C12E8 micelles, $H_2PO_4^-$ induced a fluorescence enhancement response similar to $SO_4^{2-}$ (Supplementary Figure 21 orange), only a slight fluorescence quenching response attributed to dynamic quenching was observed in POPC vesicles (Supplementary Figure 32 orange), indicating the low $H_2PO_4^-$ affinity in POPC vesicles.

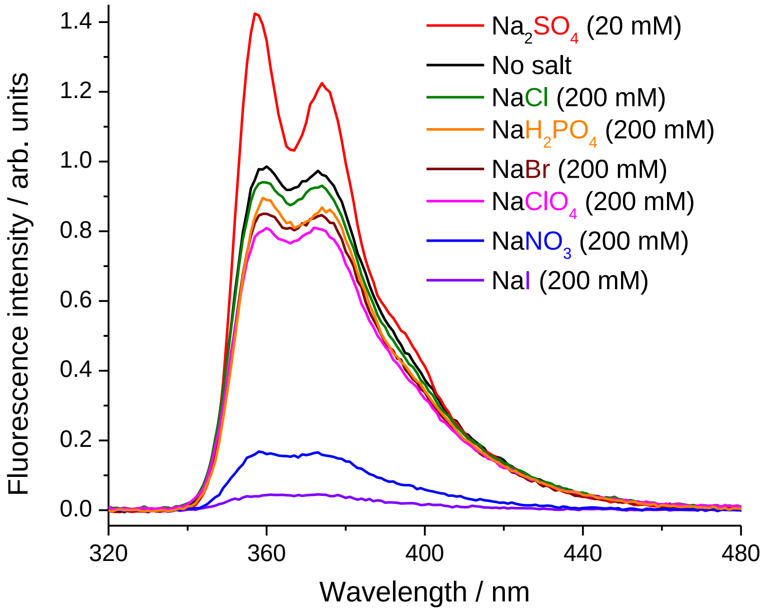


**Supplementary Figure 32** Fluorescence spectra of **1** (50 nM) in the absence and presence of salts in POPC (0.2 mM) vesicles. $\lambda_{ex}$ = 265 nm.

Competition titrations were performed with the ionic strength fixed at 0.2 M. We assumed no $Cl^-$ binding to **1** in vesicles, and hence used NaCl to balance the ionic strength. In support of this assumption, the $SO_4^{2-}$ affinities of **1** in POPC vesicles in the absence and presence of NaCl (200 mM) are very similar (350 $M^{-1}$, Supplementary Figure 31 and 300 $M^{-1}$, Supplementary Figure 33, respectively), indicating negligible $Cl^-$ binding (< 1 $M^{-1}$). The vesicles were prepared following the procedure in S8.1, except that 200 mM NaCl solution instead of pure water was used to hydrate the lipids. Fluorescence titration of **1** (50 nM) with $Na_2SO_4$ was performed following the procedure in S8.1, except that the vesicles were suspended in an isotonic NaX + NaCl (total concentration 200 mM, $X^-$ is a competing anion) solution and that only fluorescence intensities at 358 nm and 349 nm were collected to minimise photobleaching. For each competing anion, we performed competitive $Na_2SO_4$ titrations at two concentrations.

We corrected surface potential effects by determining zeta potential of POPC (2 mM) vesicles in the presence of salts via electrophoretic mobility measurements at 25 °C, using DTS1070 folded capillary cells. The vesicles after extrusion were degassed for 8 min under vacuum.[14] Note that in the case of NaI, it was necessary to reduce the applied voltage to minimise the electrolysis of $I^-$, which produces more lipophilic $I_3^-$ and enhances the electrophoretic mobility of vesicles. We also determined the size of the vesicles in the presence of salts at 200 mM and found the hydrodynamic radii to be consistently 65 ± 5 nm regardless of the identity of the salt. The zeta potential ($\zeta$) was calculated by Henry's equation (Supplementary Equation 7) using a $f(\kappa a)$ value of 1.45 calculated by the Malvern Zetasizer software. The surface potential was then calculated from the zeta potential using Supplementary Equation 8, assuming the shear plane to be 2 Å[15] from the surface (Supplementary Table 3).

**Supplementary Table 3** Zeta potential ($\zeta$) and calculated surface potential ($\Phi_0$) of POPC (2 mM) vesicles in the presence of 200 mM of salts. The values are shown as average ± SD from at least three measurements.

| Salt (200 mM) | $\zeta$ / mV | $\Phi_0$ / mV |
|---|---|---|
| $NaH_2PO_4$ | 2.4 ± 0.4 | 3.3 ± 0.6 |
| NaCl | –2.1 ± 2.0 | –2.8 ± 2.7 |
| NaBr | –5.1 ± 0.7 | –6.9 ± 1.0 |
| $NaNO_3$ | –6.3 ± 0.1 | –8.5 ± 0.1 |
| NaI | –12.6 ± 2.1 | –17.0 ± 2.9 |
| $NaClO_4$ | –19.5 ± 0.5 | –26.4 ± 0.6 |

Note that vesicles in 200 mM $NaH_2PO_4$ exhibit a positive zeta potential because of the preferential adsorption of $Na^+$ cations[14] over $H_2PO_4^-$ anions to vesicles.

For competition titrations with mixed salts (*e.g.*, 50 mM of $NaNO_3$ and 150 mM of NaCl to keep the ionic strength at 0.2 M), we calculated the $\Phi_0$ values assuming linear relationship between $\Phi_0$ and the anion fraction.[16] For $I^-$ and $ClO_4^-$, however, the relationship is not linear due to high affinities of these anions for the lipids (Table 1). In these cases, we experimentally determined the $\zeta$ and calculated the $\Phi_0$ values under mixed salt conditions (Supplementary Table 4).

**Supplementary Table 4** Zeta potential ($\zeta$) and calculated surface potential ($\Phi_0$) of POPC vesicles (2 mM) in the presence of mixed salts. The values are shown as average ± SD from at least three measurements.

| Salts | $\zeta$ / mV | $\Phi_0$ / mV |
|---|---|---|
| NaI (50 mM) + NaCl (150 mM) | –6.0 ± 0.1 | –8.1 ± 0.2 |
| NaI (100 mM) + NaCl (100 mM) | –9.4 ± 0.6 | –12.6 ± 0.8 |
| $NaClO_4$ (10 mM) + NaCl (190 mM) | –6.7 ± 0.8 | –9.0 ± 1.0 |
| $NaClO_4$ (20 mM) + NaCl (180 mM) | –9.4 ± 0.8 | –12.6 ± 1.0 |

Finally, the binding constant of **1** for a competing anion at $I$ = 0.2 is calculated using Eq 5, where $K_a(\mathrm{SO_4^{2-}})^{I=0.2\ \mathrm{M}}$ is the binding constant of **1** for $SO_4^{2-}$ in the presence of 200 mM of NaCl (assuming no affinity of $Cl^-$) after correction against the slightly negative surface potential of vesicles in a 200 mM NaCl solution.

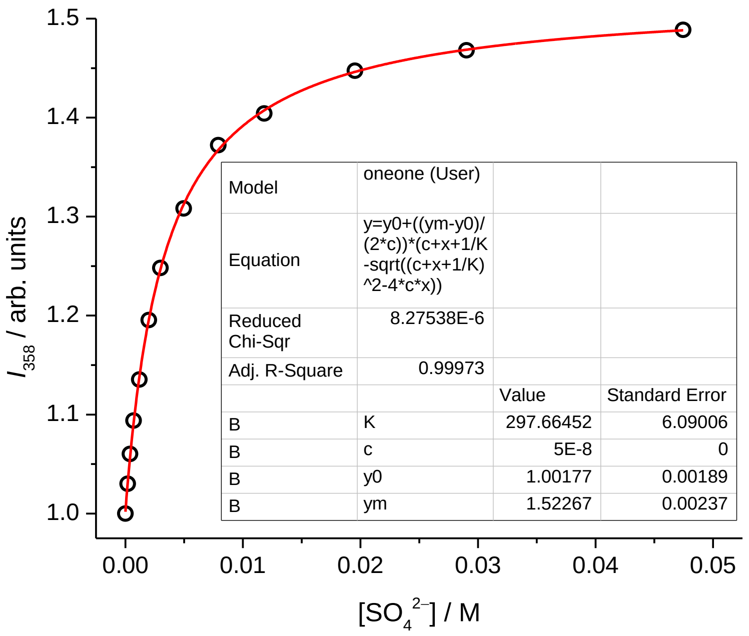


**Supplementary Figure 33** Fluorescence titration of **1** (50 nM) with $Na_2SO_4$ in the presence of NaCl (200 mM) in POPC (0.2 mM) vesicles. The data was fitted to a 1:1 binding model, giving an apparent $SO_4^{2-}$ binding constant of 300 ± 10 $M^{-1}$ from two titrations. This is converted to 370 ± 10 $M^{-1}$ after correction against the $\Phi_0$ of –2.8 mV (Supplementary Table 2).

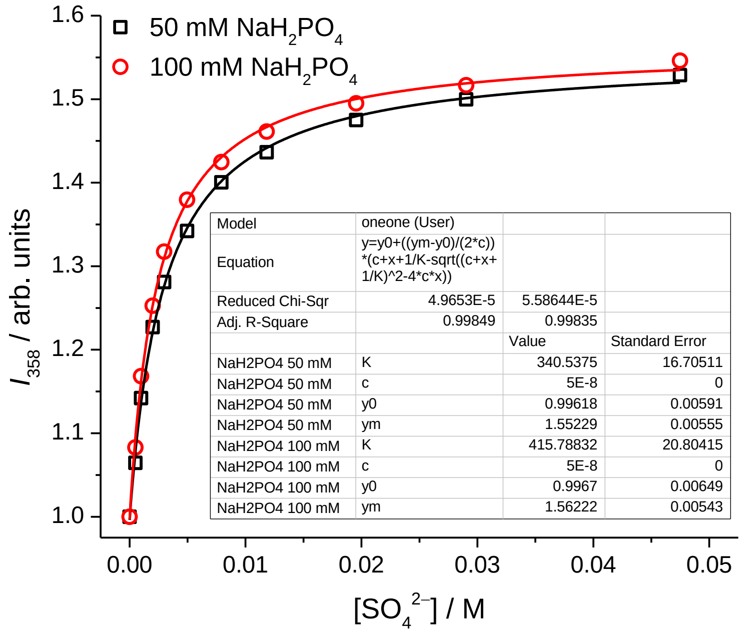


**Supplementary Figure 34** Competition fluorescence titration of **1** (50 nM) with $Na_2SO_4$ in the presence of $NaH_2PO_4$ (50 and 100 mM) and NaCl (150 and 100 mM, respectively, to fix the ionic strength at 0.2 M) in POPC (0.2 mM) vesicles. The data was fitted to a 1:1 binding model, giving apparent $SO_4^{2-}$ binding constants of 340 and 420 $M^{-1}$ with $NaH_2PO_4$ present at 50 and 100 mM, respectively. From these values, the $H_2PO_4^-$ binding constant of **1** was calculated to be –0.7 ± 0.3 $M^{-1}$ using Supplementary Equation 10, indicating a very low $H_2PO_4^-$ affinity.

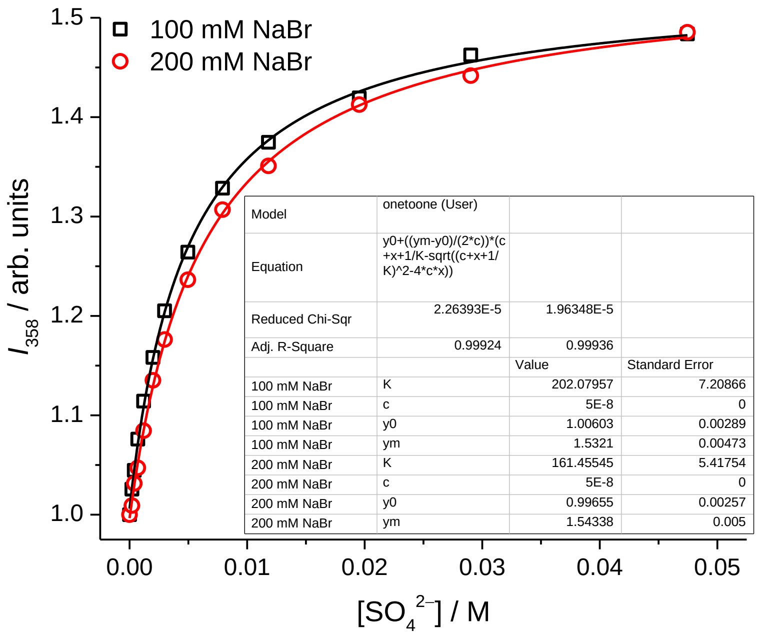


**Supplementary Figure 35** Competition fluorescence titration of **1** (50 nM) with $Na_2SO_4$ in the presence of NaBr (100 and 200 mM) and NaCl (100 and 0 mM, respectively, to fix the ionic strength at 0.2 M) in POPC (0.2 mM) vesicles. The data was fitted to a 1:1 binding model, giving apparent $SO_4^{2-}$ binding constants of 200 and 160 $M^{-1}$ with NaBr present at 100 and 200 mM, respectively. From these values, the $Br^-$ binding constant of **1** was calculated to be 2.6 ± 0.6 $M^{-1}$ using Supplementary Equation 10.

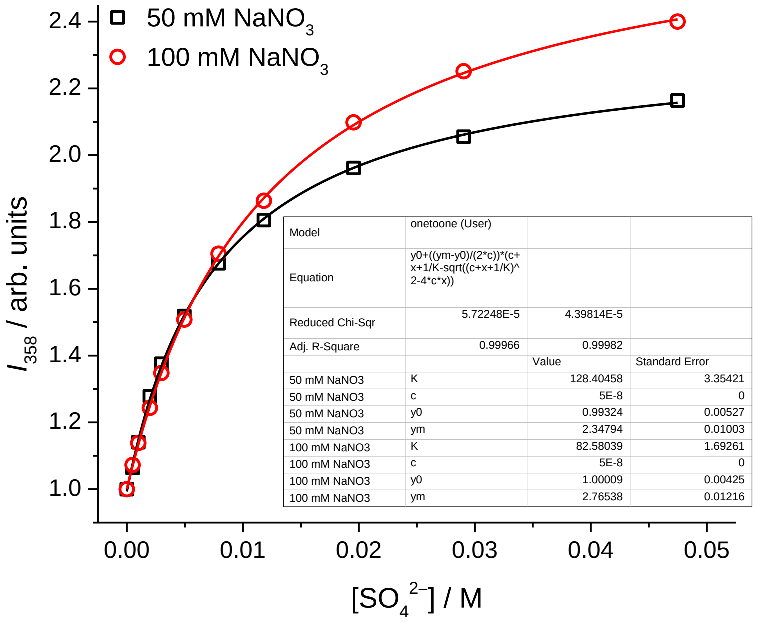


**Supplementary Figure 36** Competition fluorescence titration of **1** (50 nM) with $Na_2SO_4$ in the presence of $NaNO_3$ (50 and 100 mM) and NaCl (150 and 100 mM, respectively, to fix the ionic strength at 0.2 M) in POPC (0.2 mM) vesicles. The data was fitted to a 1:1 binding model, giving apparent $SO_4^{2-}$ binding constants of 130 and 83 $M^{-1}$ with $NaNO_3$ at 50 and 100 mM, respectively. From these values, the $NO_3^-$ binding constant of **1** was calculated to be 24 ± 4 $M^{-1}$ using Supplementary Equation 10.

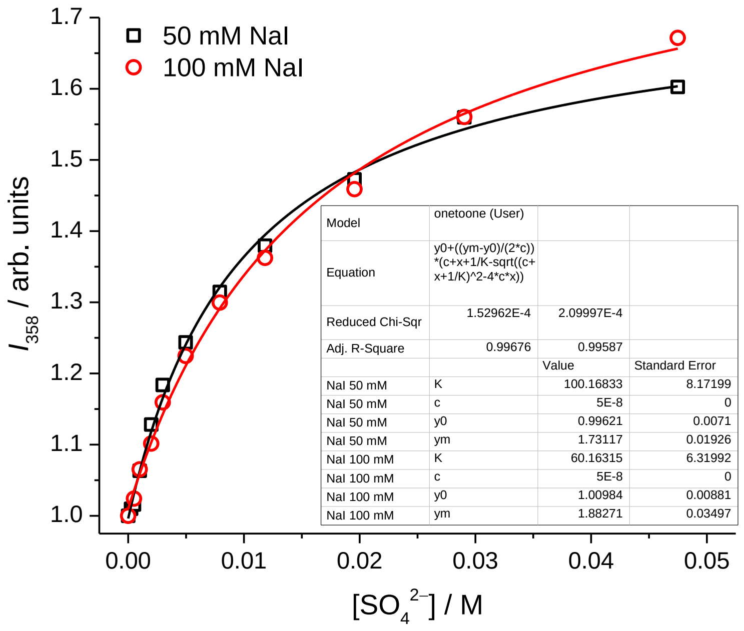


**Supplementary Figure 37** Competition fluorescence titration of **1** (50 nM) with $Na_2SO_4$ in the presence of NaI (50 and 100 mM) and NaCl (150 and 100 mM, respectively, to fix the ionic strength at 0.2 M) in POPC (0.2 mM) vesicles. The data was fitted to a 1:1 binding model, giving apparent $SO_4^{2-}$ binding constants of 100 and 60 $M^{-1}$ with NaI at 50 and 150 mM, respectively. From these values, the $I^-$ binding constant of **1** was calculated to be 24 ± 2 $M^{-1}$ using Supplementary Equation 10.

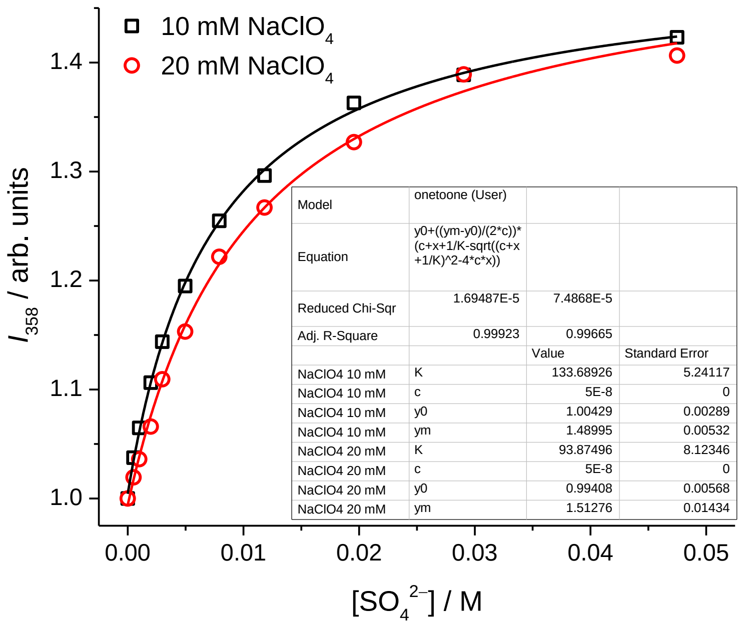


**Supplementary Figure 38** Competition fluorescence titration of **1** (50 nM) with $Na_2SO_4$ in the presence of $NaClO_4$ (10 and 20 mM) and NaCl (190 and 180 mM, respectively, to fix the ionic strength at 0.2 M) in POPC (0.2 mM) vesicles. The data was fitted to a 1:1 binding model, giving apparent $SO_4^{2-}$ binding constants of 130 and 94 $M^{-1}$ with $NaClO_4$ at 10 and 20 mM, respectively. From these values, the $ClO_4^-$ binding constant of **1** was calculated to be 45 ± 9 $M^{-1}$ using Supplementary Equation 10.

# S8. Control fluorescence studies

To verify that the formation of the **1**-$SO_4^{2-}$ complex occurs within C12E8 micelles or POPC membrane, instead of in the bulk solution, we recorded the fluorescence spectra of **1** in the absence and presence of $Na_2SO_4$ in pure water. 12.5 µL of a DMSO solution of **1**-$SO_4^{2-}$ (10 µM) was added to 2.5 mL of water or a $Na_2SO_4$ (20 mM) solution in water, stirred and thermostated at 25 °C in a 4 mL quartz cuvette. Supplementary Figure 39 shows that neither the free macrocycle **1** or its $SO_4^{2-}$ complex is fluorescent in pure water due to insolubility, confirming that $SO_4^{2-}$ binding occurs in the micelle/membrane phase instead of in the bulk solution.

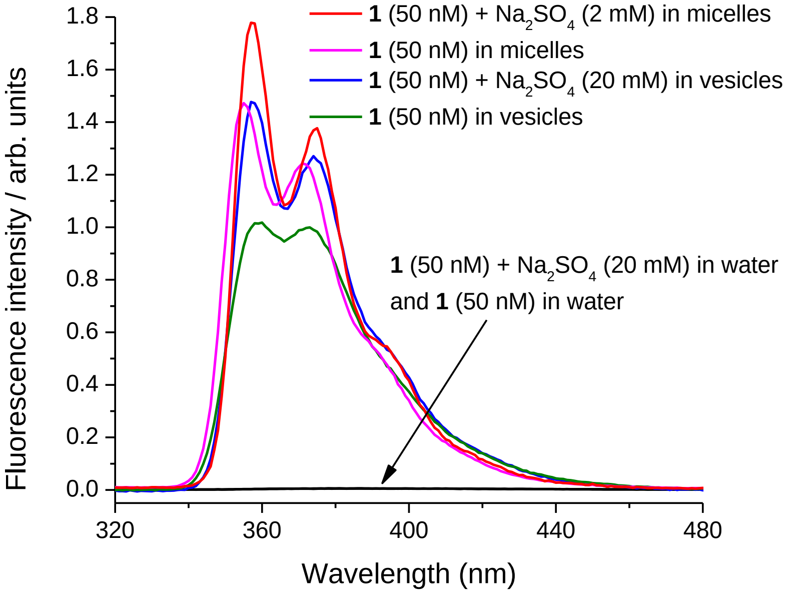


**Supplementary Figure 39** Comparison of fluorescence spectra of **1** (50 nM) with and without $Na_2SO_4$ in C12E8 (2 mM) micelles, POPC (0.2 mM) vesicles and pure water. $\lambda_{ex}$ = 265 nm.

We also examined the $SO_4^{2-}$ responses of an acyclic carbazole bis-urea receptor **3**[17] (Supplementary Figure 40) in C12E8 micelles and POPC vesicles. 12.5 µL of a DMSO solution of **3** (10 µM) was added to 2.5 mL of C12E8 (2 mM) micellar solution or POPC (0.2 mM) vesicle suspension, and then $Na_2SO_4$ was added, either from a 1.0 M stock solution (to a final concentration of 20 mM), or as a solid (to 200 mM). For the fluorescence spectrum in POPC vesicles, the scattering baseline of POPC was subtracted. Receptor **3** demonstrates a weak (Supplementary Figure 41) or negligible (Supplementary Figure 42) fluorescence response to $SO_4^{2-}$. The weak fluorescence enhancement observed with 200 mM of $SO_4^{2-}$ in C12E8 micelles (Supplementary Figure 41) is likely due to slightly increased solubility of **3** because the shape of the fluorescence spectrum with $SO_4^{2-}$ is identical to that of the free receptor. Therefore, the strong fluorescence response to $SO_4^{2-}$ is unique to macrocycle **1**.

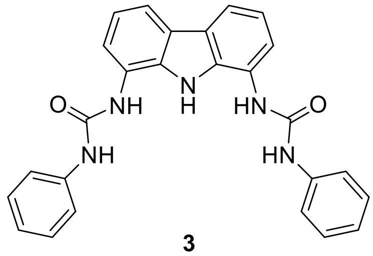


**3**

**Supplementary Figure 40** Structure of a carbazole bis-urea receptor **3**.

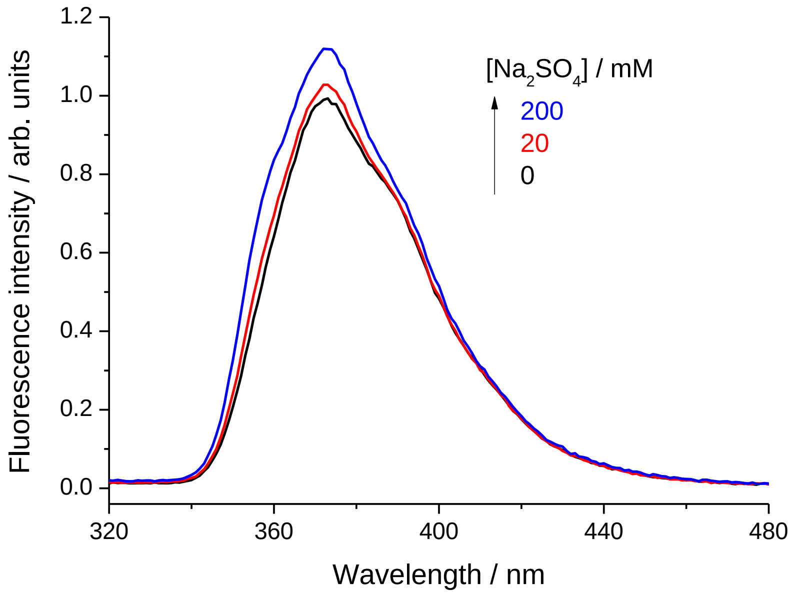


**Supplementary Figure 41** Fluorescence spectra of **3** (50 nM) with increasing concentrations of $Na_2SO_4$ in C12E8 (2 mM) micelles in $H_2O$. $\lambda_{ex}$ = 265 nm.

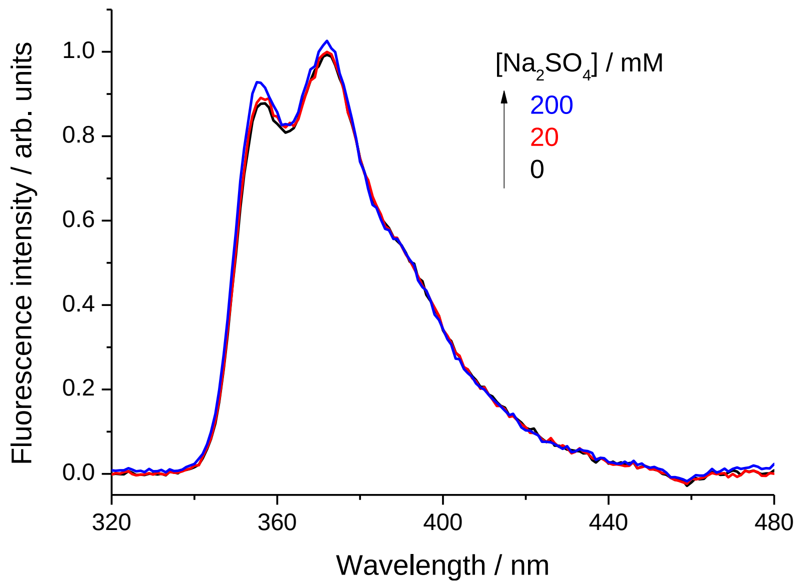


**Supplementary Figure 42** Fluorescence spectra of **3** (50 nM) with increasing concentrations of $Na_2SO_4$ in POPC (0.2 mM) vesicles. $\lambda_{ex}$ = 265 nm.

## S9. Fluorescence penetration depth studies

Nitroxide spin-labelled lipids (Supplementary Figure 43) were used to determine the penetration depth of **1** and its anion complexes.[18]

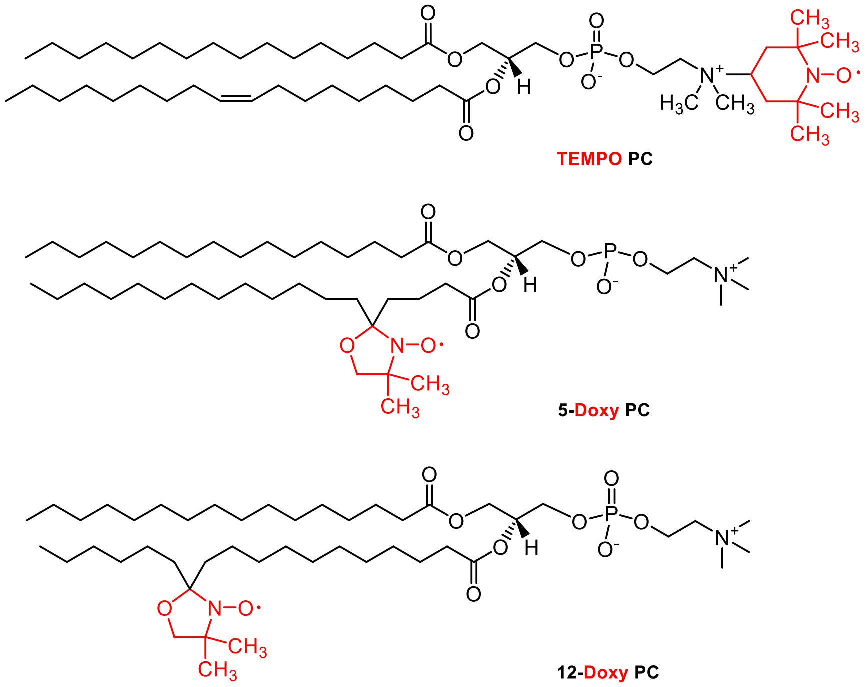


**Supplementary Figure 43** Spin-labelled lipids for fluorescence-quenching penetration depth studies.

A 1 mg/mL chloroform solution of a spin-labelled lipid was mixed with a 1 g / 35 mL chloroform solution of POPC in a 10 mL round-bottom flask, to a labelled/unlabelled lipid molar ratio of 1:9 and a total lipid amount of 3.2 μmol. The chloroform was evaporated and the lipid film was dried under vacuum for at least 6 hours. The lipids were then hydrated with 1 mL of Milli-Q water to form vesicles, vortexed, and then subject to 9 freeze-thaw cycles. The vesicles were extruded 39 times through a 100 nm polycarbonate membrane, to obtain a vesicle stock solution with a total lipid concentration of 3.2 mM. For each experiment, the lipids were diluted to a total lipid concentration of 0.2 mM in 2.5 mL of water, a 20 mM $Na_2SO_4$ solution, or a 200 mM $NaClO_4$ solution. The solution in a 4 mL quartz cuvette was stirred and thermostated at 25 °C. The spectrum ($\lambda_{ex}$ = 265 nm, $\lambda_{em}$ = 300–500 nm) of vesicles without **1** was collected for baseline subtraction. 12.5 μL of a DMSO solution of **1**-$SO_4^{2-}$ (10 μM) was added to 2.5 mL of the diluted vesicle suspension, to a final macrocycle concentration of 50 nM. Because of the low concentration used, the **1**-$SO_4^{2-}$ complex completely dissociated into free macrocycle **1**. Fluorescence spectra ($\lambda_{ex}$ = 265 nm, $\lambda_{em}$ = 300–500 nm) of **1** in the absence and presence of $Na_2SO_4$ (20 mM) and $NaClO_4$ (200 mM) with 9:1 POPC/TEMPO PC, 9:1 POPC/5-doxyl PC, 9:1 POPC/12-doxyl PC, and 100% POPC were recorded. Here the fluorescence contribution from **1** in the solution phase[18] is negligible (Supplementary Figure 39). The following equation was used to calculate the membrane penetration depth of **1** using the data obtained with the strongest quenching pair (*i.e.*, TEMPO PC and 5-doxyl PC):

$$z_{cf} = L_{c1} + \left[ -\frac{\ln\left(\frac{F1}{F2}\right)}{\pi C} - L_{21}^2 \right] / 2L_{21} \qquad \text{Supplementary Equation 11}$$

where $z_{cf}$ is the distance of macrocycle **1** from the bilayer centre, $F_1$ is the fluorescence intensity (we used the intensity at 360 nm) of macrocycle **1** in the presence of quencher 1 (the shallow quencher TEMPO PC), $F_2$ is the fluorescence intensity (we used the intensity at 360 nm) of macrocycle **1** in the presence of quencher 2 (the deeper quencher 5-doxyl PC), $L_{c1}$ is the distance of quencher 1 from the bilayer centre, $L_{21}$ is the distance between quenchers 1 and 2, $C$ is the quencher concentration in

molecules/Å (= mole fraction of spin-labelled lipids/70 Å$^2$). The distances of the nitroxide quencher from bilayer centre was previously estimated to be 19.5 Å for TEMPO PC and 12.2 Å for 5-doxyl PC.[18]

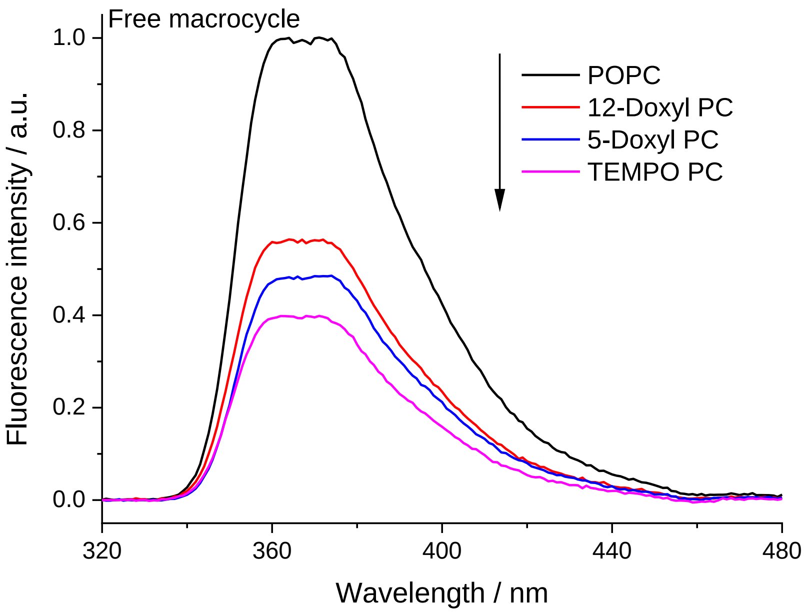


**Supplementary Figure 44** Fluorescence spectra of **1** (50 nM) in pure POPC (0.2 mM) vesicles, or POPC vesicles containing 10 mol% of spin-labelled lipids (total lipid concentration 0.2 mM). $\lambda_{ex}$ = 265 nm. Here we used a higher slit width than in Supplementary Figure 39, which caused a slight signal broadening. A penetration depth of 19 Å was calculated using Supplementary Equation 11, from a fluorescence intensity ratio (at 360 nm) of 0.82 between TEMPO PC and 5-doxyl PC.

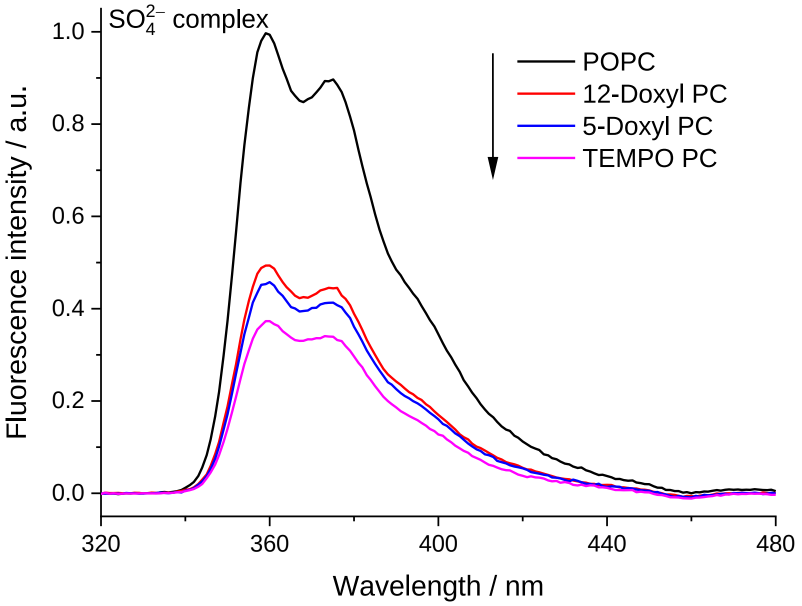


**Supplementary Figure 45** Fluorescence spectra of **1** (50 nM) in the presence of 20 mM of $Na_2SO_4$ in pure POPC (0.2 mM) vesicles, or POPC vesicles containing 10 mol% of spin-labelled lipids (total lipid concentration 0.2 mM). $\lambda_{ex}$ = 265 nm. Here we used a higher slit width than in Supplementary Figure 39, which caused a slight signal broadening. A penetration depth of 19 Å was calculated using Supplementary Equation 11, from a fluorescence intensity ratio (at 360 nm) of 0.83 between TEMPO PC and 5-doxyl PC.

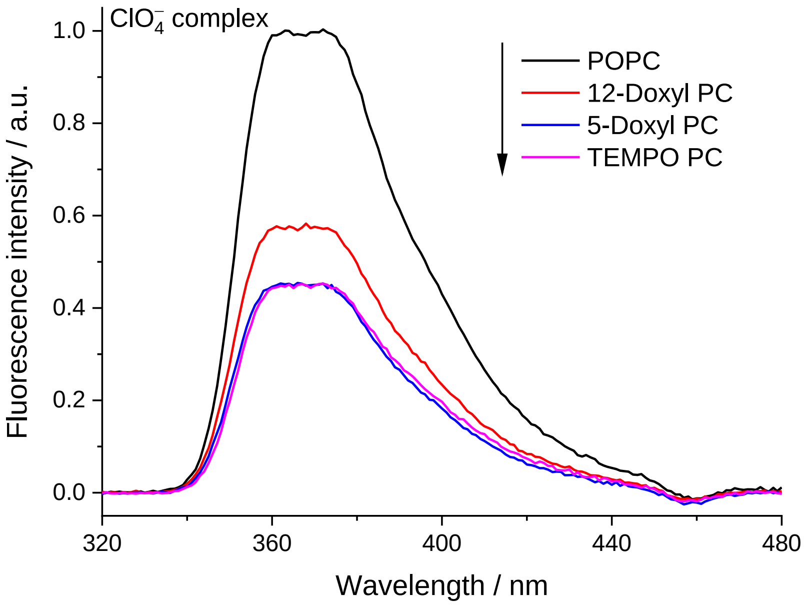


**Supplementary Figure 46** Fluorescence spectra of **1** (50 nM) in the presence of 200 mM of $NaClO_4$ in pure POPC (0.2 mM) vesicles, or POPC vesicles containing 10 mol% of spin-labelled lipids (total lipid concentration 0.2 mM). $\lambda_{ex}$ = 265 nm. Here we used a higher slit width than in Supplementary Figure 39, which caused a slight signal broadening. A penetration depth of 16 Å was calculated using Supplementary Equation 11, from a fluorescence intensity ratio (at 360 nm) of 0.99 between TEMPO PC and 5-doxyl PC.

# S10. Transmembrane anion transport

## S10.1 HPTS assay

We have decided to use the salt-pulse assay[19] for quantitative determination of anion transport rates and selectivity, because the more commonly used base-pulse assay is compromised by potential anion-dependent transporter partitioning[20] and surface charge effects due to membrane adsorption of chaotropic anions.[16,21,22] These effects are more significant in the base-pulse assay (anions used at 100 mM) than in the salt-pulse assay (anion concentration can be reduced to 20 mM or lower). In this assay, the anion transporter facilitates anion$^-$ and H$^+$ influx driven by the anion concentration gradient, leading to acidification of the vesicle interior as monitored by the ratiometric fluorescence response of the intra-vesicular pH indicator HPTS. We used sodium gluconate (NaGluc) as the main osmolyte because Gluc$^-$ is a large, hydrophilic anion that cannot be transported across the membrane.

The assay was conducted using POPC LUVs (mean diameter 200 nm) loaded with the pH-sensitive fluorescence dye HPTS (1 mM). The HPTS-loaded POPC LUVs were prepared as follows. 2 mL of a chloroform solution of POPC (1 g in 35 mL) was evaporated in a round-bottom flask and the lipid film formed was dried under vacuum for at least 6 h. Then, the lipid film was hydrated by vortexing with a 2 mL internal solution containing HPTS (1 mM) and NaGluc (100 mM) buffered at pH 7.0 with 10 mM HEPES. The lipid suspension was subjected to nine freeze/thaw cycles and then extruded 25 times through a 200 nm polycarbonate membrane. The unentrapped HPTS was removed by a Sephadex G-25 column using an external solution of NaGluc (100 mM) buffered at pH 7.0 with 10 mM HEPES as the eluent, to obtain a vesicle stock suspension with POPC concentration of ~10 mM. The concentration of POPC in the stock suspension was calculated based on the weight difference of the round bottom flask before POPC addition and after drying, and the volume of the lipid suspension after the Sephadex column, taking into account the loss of lipids during the extrusion.

For each measurement, the vesicles were diluted to a final POPC concentration of 0.10 mM. The sample in a 4 mL disposable polystyrene cuvette was stirred and thermostated at 25 °C. The ratiometric fluorescence response of HPTS $I_{460}/I_{403}$ ($\lambda_{ex}$ = 460 nm, $\lambda_{em}$ = 510 nm divided by $\lambda_{ex}$ = 403 nm, $\lambda_{em}$ = 510 nm) was recorded using a fluorometer. 5 μL of a DMSO solution of **1**-$SO_4^{2-}$ (0.5 mM) was added (final concentration of **1** was 1.0 μM or 1.0 mol% with respect to lipids) at t = −50 s, followed by a salt pulse of 20 mM NaX ($X^-$ = Gluc$^-$, $SO_4^{2-}$, $H_2PO_4^-$, Cl$^-$, Br$^-$, $NO_3^-$, I$^-$, or $ClO_4^-$, using 5 M or 1 M stock solutions) at t = 0 s. For $H_2PO_4^-$, the $NaH_2PO_4$ stock solution was adjusted to pH 7.0 using $Na_2HPO_4$ prior to addition to the vesicles. The transport kinetics was monitored for at least 200 s.

To convert the raw HPTS fluorescence data into transport rates in number of anions per second per carrier, we first performed a calibration of the HPTS response against pH for NaGluc$^{in}$/NaGluc$^{out}$ vesicles using a previously described procedure.[20] Here the relationship between the pH ($y$) and $I_{460}/I_{403}$ of HPTS ($x$) is described by the following derived from the Henderson-Hasselbalch equation:

$$y = \log_{10}\left(\frac{\mathrm{a}x-\mathrm{b}}{\mathrm{c}-x}\right) \qquad \text{Supplementary Equation 12}$$

where the values of parameters a, b and c are shown in Supplementary Figure 47.

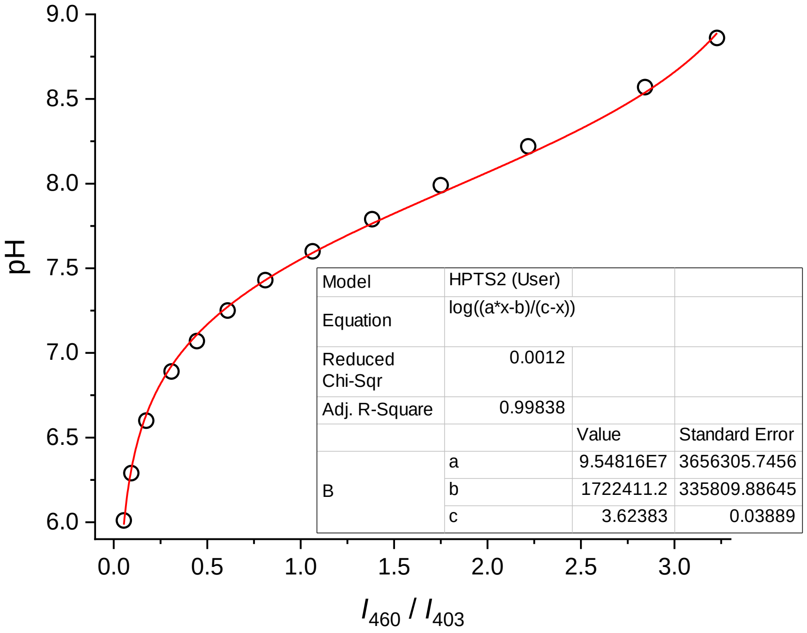


**Supplementary Figure 47** Calibration for the HPTS assay using NaGluc vesicles.

Based on Supplementary Equation 12 which converts pH to $I_{460}/I_{403}$, we then simulated the relationship between $H^+$ influx ($[\Delta H^+]_{in}$) and the change in the fluorescence ratio $\Delta(I_{460}/I_{403})$, using the the Henderson-Hasselbalch equation for HEPES buffer to convert ($[\Delta H^+]_{in}$) into pH:

$$\mathrm{pH} = 7.54 + \log_{10}\left(\frac{2.24\ \mathrm{mM} - [\Delta H^+]_{in}}{7.76\ \mathrm{mM} + [\Delta H^+]_{in}}\right) \qquad \text{Supplementary Equation 13}$$

where 7.54 is the apparent $pK_a$ of HEPES at $I$ = 0.1 M, and 2.24 mM & 7.76 mM are the initial concentrations of the base and acid forms of HEPES, respectively. The simulated curve, shown in Supplementary Figure 48, can be approximated by a linear function which converts experimental $\Delta(I_{460}/I_{403})$ value into $[\Delta H^+]_{in}$. Note that we have previously demonstrated a linear relationship experimentally.[23]

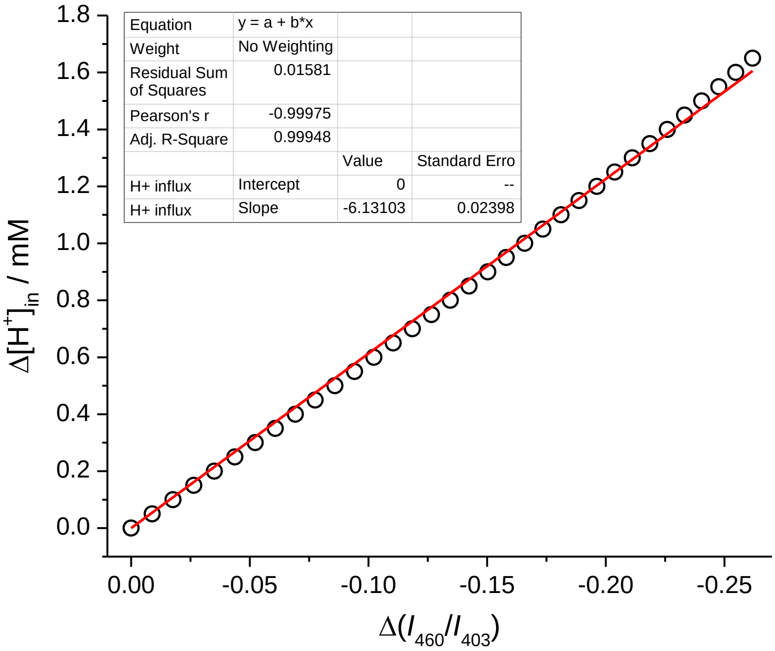


**Supplementary Figure 48** Simulated relationship between the change of HPTS fluorescence ratio ($\Delta(I_{460}/I_{403})$ ) and $H^+$ influx ($[\Delta H^+]_{in}$), based on the HPTS calibration and the the Henderson-Hasselbalch equation for HEPES buffer. This relationship can be approximated by a linear function.

After converting the experimental $\Delta(I_{460}/I_{403})$ values into $[\Delta H^+]_{in}$, we fitted the kinetic curves to the biphasic exponential decay function (ExpDec2) using OriginPro:

$$y = y_0 + A_1 e^{-x/t_1} + A_2 e^{-x/t_2} \qquad \text{Supplementary Equation 14}$$

The initial rate ($k$) is then calculated using the following equation:

$$k = -A_1/t_1 - A_2/t_2 \qquad \text{Supplementary Equation 15}$$

Finally, the initial rate of $H^+$ influx in mM $s^{-1}$ using a transporter at a given mol% loading is converted to anions $s^{-1}$ carrier$^{-1}$, using the following equation obtained after taking literature values of the vesicle volume and the number of POPC molecules per vesicle for 200 nm (diameter) POPC LUVs,[24] noting that the influx of each $H^+$ is accompanied by an anion (for monovalent anions):

$$\text{transport rate in anions s}^{-1}\text{ carrier}^{-1} = \frac{0.67 \times \text{transport rate in mM s}^{-1}}{\text{carrier loading in mol\% with respect to lipid}}$$

Supplementary Equation 16

For $SO_4^{2-}$, the value calculated by Supplementary Equation 16 was divided by 2.

Note that: (1) Here without correction of the carrier deliverability,[25] the carrier refers to all carriers added into the solution instead of active carriers that partitioned in the membrane; (2) The rate depends on the ion concentration used and usually increases linearly with increasing ion concentration until the carriers are saturated by ion binding.[26]

## S10.2 Osmotic response assay

The HPTS assay has established macrocycle **1** as an $H^+/X^-$ symporter. To examine whether **1** can also function as an $X^-$ uniporter, we performed an osmotic response assay to monitor $NO_3^-$ transport in vesicles (Supplementary Figure 49). In this assay, under the conditions shown in Supplementary Figure 49a, the anion transporter needs to facilitate $NO_3^-$ uniport to couple to $K^+$ uniport by valinomycin (Vln) to give overall $KNO_3$ efflux. In Supplementary Figure 49b, the anion transporter needs to facilitate $H^+/NO_3^-$ symport to couple to $K^+/H^+$ antiport facilitated by monensin to give overall $KNO_3$ efflux.[27] $KNO_3$ efflux causes osmotic shrinkage and an increase of light scattering intensity of vesicles as monitored using a fluorometer.[28]

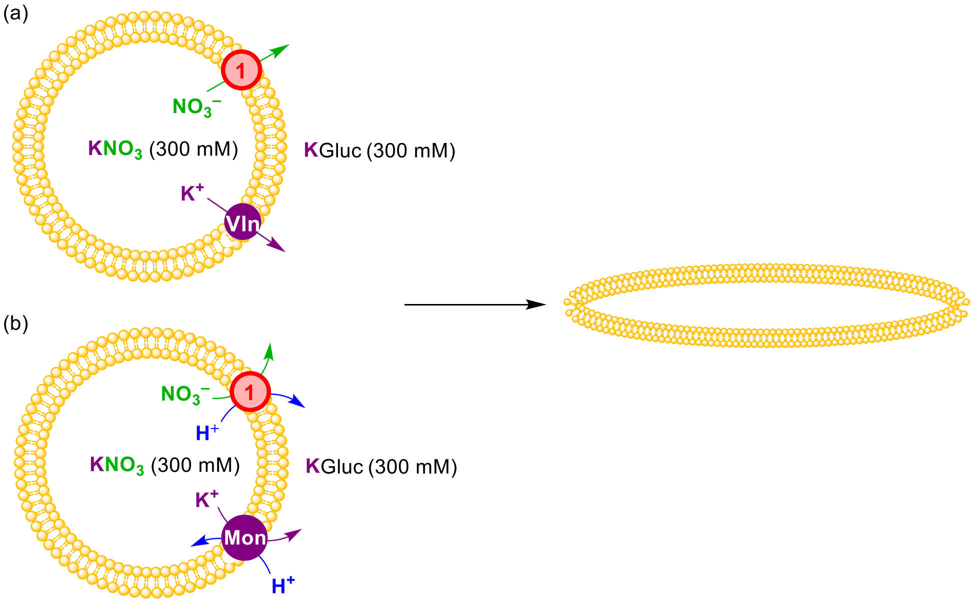


**Supplementary Figure 49** Schematic representation of the osmotic response assay to investigate $NO_3^-$ uniport (a, with valinomycin, Vln) and $H^+/NO_3^-$ symport (b, with monensin, Mon) facilitated by **1**.

The assay was conducted using POPC LUVs (mean diameter 400 nm) loaded with $KNO_3$ (300 mM) and suspended in KGluc (300 mM). Both the internal and external solutions were buffered at pH 7.0 with 10 mM of HEPES. The POPC LUVs were prepared as follows. 2 mL of a chloroform solution of POPC (1 g in 35 mL) was evaporated in a round-bottom flask and the lipid film formed was dried under vacuum for at least 6 h. Then, the lipid film was hydrated by vortexing with a 2 mL internal solution of $KNO_3$ (300 mM) buffered at pH 7.0. The lipid suspension was subjected to nine freeze/thaw cycles and then extruded 21 times through a 400 nm polycarbonate membrane. The concentration of POPC (~40 mM) was calculated based on the weight difference of the round bottom flask before POPC addition and after drying.

For each measurement, the vesicles were diluted to a final POPC concentration of 0.2 mM using an external KGluc (300 mM) solution buffered at pH 7.0. The sample in a 4 mL disposable polystyrene cuvette was stirred and thermostated at 25 °C. The 90° light scattering intensity of vesicles ($\lambda_{ex}$ = 600 nm, $\lambda_{em}$ = 600 nm) was recorded by a fluorometer. 10 µL of a DMSO solution of **1**-$SO_4^{2-}$ (0.5 mM) was added (final concentration of **1** was 2 µM or 1 mol% with respect to lipids), followed by valinomycin (0.1 µM, 0.05 mol%) or monensin (1 µM, 0.5 mol%) at t = 0 s. The transport kinetics was monitored for at least 1 h. At the end of the experiment, *N*,*N'*-bis[3,5-bis(trifluoromethyl)phenyl]-thiourea (4 µM, 2 mol%, 1 µM of monensin was also added if no cationophore was present previously) was added to complete the salt transport and normalise the light scattering intensity to 100% salt efflux.

Macrocycle **1** induced significant $NO_3^-$ transport in the presence of monensin, but did not increase $NO_3^-$ transport relative to the valinomycin baseline (Supplementary Figure 50), confirming that **1** functions as a $H^+/NO_3^-$ symporter but not as a $NO_3^-$ uniporter. Note that salt efflux could be observed with valinomycin alone, which is attributed to facilitated transport via a valinomycin-$KNO_3$ ion pair complex.[29]

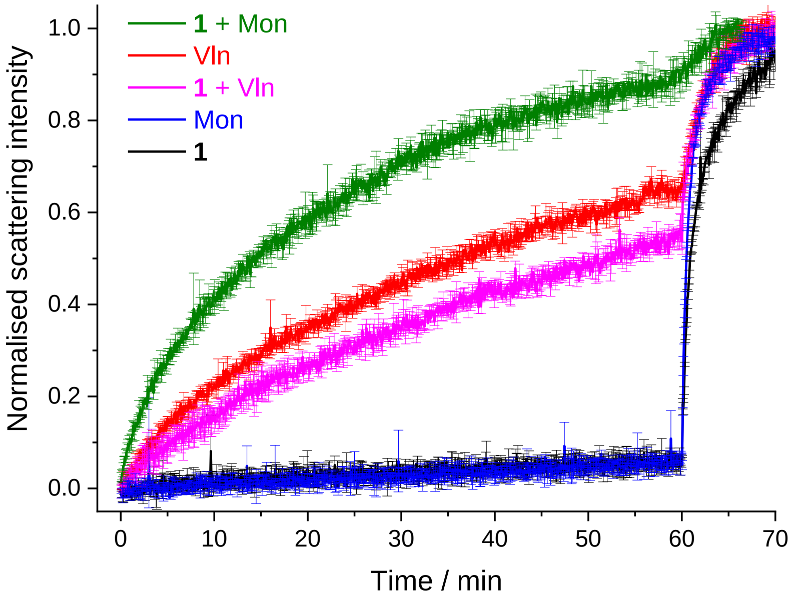


**Supplementary Figure 50** $KNO_3$ transport by **1** (1 mol%), valinomycin (Vln, 0.05 mol%), monensin (Mon, 0.5 mol%), **1** + Vln, and **1** + Mon. At 60 min, *N*,*N'*-bis[3,5-bis(trifluoromethyl)phenyl]-thiourea (2 mol%, 0.5 mol% of monensin was also added if no cationophore was present previously) was added to complete salt transport and normalise the light scattering intensity to 100% salt efflux. Error bars represent SD from two experiments.

# Supplementary References


1 Bąk, K. M., Chabuda, K., Montes, H., Quesada, R. & Chmielewski, M. J. 1,8-Diamidocarbazoles: an easily tuneable family of fluorescent anion sensors and transporters. *Org. Biomol. Chem.* **16**, 5188-5196 (2018).
2 Dolomanov, O. V., Bourhis, L. J., Gildea, R. J., Howard, J. A. K. & Puschmann, H. OLEX2: a complete structure solution, refinement and analysis program. *J. Appl. Crystallogr.* **42**, 339-341 (2009).
3 Bourhis, L. J., Dolomanov, O. V., Gildea, R. J., Howard, J. A. K. & Puschmann, H. The anatomy of a comprehensive constrained, restrained refinement program for the modern computing environment - Olex2 dissected. *Acta Crystallogr. A* **71**, 59-75 (2015).
4 Sheldrick, G. Crystal structure refinement with SHELXL. *Acta Crystallogr. C* **71**, 3-8 (2015).
5 Bauzá, A., Frontera, A. & Mooibroek, T. J. $NO_3^-$ anions can act as Lewis acid in the solid state. *Nat. Commun.* **8**, 14522 (2017).
6 http://app.supramolecular.org/bindfit/.
7 Gale, P. A. *et al.* Anion–Anion Proton Transfer in Hydrogen Bonded Complexes. *Chem. Asia J.* **5**, 555-561 (2010).
8 Fillingham, R., Boon, M., Javaid, S., Saunders, J. A. & Jones, F. Barium sulfate crystallization in non-aqueous solvent. *CrystEngComm* **23**, 2249-2261 (2021).
9 Dunnett, J. S. & Gasser, R. P. H. Electrolyte solutions in dimethyl sulphoxide. Part 1.—Lithium chloride. *Transactions of the Faraday Society* **61**, 922-927 (1965).
10 Gibson, H. W., Jones, J. W., Zakharov, L. N., Rheingold, A. L. & Slebodnick, C. Complexation Equilibria Involving Salts in Non-Aqueous Solvents: Ion Pairing and Activity Considerations. *Chem. Eur. J.* **17**, 3192-3206 (2011).
11 Murgia, S., Monduzzi, M. & Palazzo, G. Quantification of Specific Anion Binding to Non-Ionic Triton X-100 Micelles. *Langmuir* **28**, 1283-1289 (2012).
12 Sathappa, M. & Alder, N. N. Ionization Properties of Phospholipids Determined by Zeta Potential Measurements. *Bio-protocol* **6**, e2030 (2016).
13 Yang, Z., Zhao, J., Lu, Y., Du, Z. & Yang, Z. Ultramicroelectrode voltammetric measurement of the hydrodynamic radii and shear plane of micellar particles for sodium dodecylsulfonate in aqueous NaCl solution. *Chem. Phys.* **307**, 71-75 (2004).
14 Klasczyk, B., Knecht, V., Lipowsky, R. & Dimova, R. Interactions of Alkali Metal Chlorides with Phosphatidylcholine Vesicles. *Langmuir* **26**, 18951-18958 (2010).
15 Eisenberg, M., Gresalfi, T., Riccio, T. & McLaughlin, S. Adsorption of monovalent cations to bilayer membranes containing negative phospholipids. *Biochemistry* **18**, 5213-5223 (1979).
16 Clarke, R. J. & Lüpfert, C. Influence of Anions and Cations on the Dipole Potential of Phosphatidylcholine Vesicles: A Basis for the Hofmeister Effect. *Biophys. J.* **76**, 2614-2624 (1999).
17 Wang, P., Wu, X. & Gale, P. A. Carbazole-based bis-ureas and thioureas as electroneutral anion transporters. *Supramol. Chem.*, DOI: 10.1080/10610278.10612021.11946539 (2021).
18 Kondo, M., Mehiri, M. & Regen, S. L. Viewing Membrane-Bound Molecular Umbrellas by Parallax Analyses. *J. Am. Chem. Soc.* **130**, 13771-13777 (2008).
19 Milano, D. *et al.* Anion transport across phospholipid membranes mediated by a diphosphine-Pd(II) complex. *Chem. Commun.* **50**, 9157-9160 (2014).
20 Wu, X. & Gale, P. A. Measuring anion transport selectivity: a cautionary tale. *Chem. Commun.* **57**, 3979-3982 (2021).
21 Tatulian, S. A. Binding of alkaline-earth metal cations and some anions to hosphatidylcholine liposomes. *Eur. J. Biochem.* **170**, 413-420 (1987).
22 Rydall, J. R. & Macdonald, P. M. Investigation of anion binding to neutral lipid membranes using deuterium NMR. *Biochemistry* **31**, 1092-1099 (1992).
23 Clarke, H. J., Wu, X., Light, M. E. & Gale, P. A. Selective anion transport mediated by strap-extended calixpyrroles. *J. Porphyr. Phthalocya* **24**, 473-479 (2020).
24 Luisi, P. L. *The Emergence of Life: From Chemical Origins to Synthetic Biology*. (Cambridge University Press, 2006).

25 Li, H. *et al.* Efficient, non-toxic anion transport by synthetic carriers in cells and epithelia. *Nat. Chem.* **8**, 24-32 (2016).
26 Läuger, P. Carrier-mediated ion transport: electrical relaxation experiments give insight into the kinetics of ion transport through artificial lipid membrane. *Science* **178**, 24-30 (1972).
27 Wu, X., Howe, E. N. W. & Gale, P. A. Supramolecular Transmembrane Anion Transport: New Assays and Insights. *Acc. Chem. Res.* **51**, 1870-1879 (2018).
28 Stockbridge, R. B. *et al.* Fluoride resistance and transport by riboswitch-controlled CLC antiporters. *Proc. Natl. Acad. Sci. USA* **109**, 15289-15294 (2012).
29 Su, Z. *et al.* How Valinomycin Ionophores Enter and Transport $K^+$ across Model Lipid Bilayer Membranes. *Langmuir* **35**, 16935-16943 (2019).
