# Supplementary material for: Measuring anion binding at biomembrane interfaces
Source: Nat Commun. 2022 Aug 8;13:4623. doi: 10.1038/s41467-022-32403-z (PMC9359984; doi:10.1038/s41467-022-32403-z)
Supplement: Supplementary file 2 — Description of Additional Supplementary Files [file 41467_2022_32403_MOESM2_ESM.pdf]

Supplementary Data File 1: xyz coordinates for the optimized structures
