# Supplementary material for: Measuring anion binding at biomembrane interfaces
Source: Nat Commun. 2022 Aug 8;13:4623. doi: 10.1038/s41467-022-32403-z (PMC9359984; doi:10.1038/s41467-022-32403-z)
Supplement: Supplementary file 3 — Supplementary Data 1 [file 41467_2022_32403_MOESM3_ESM.pdf]

1-Br

N	3.868356	0.000000	0.000000
C	4.677724	1.128725	0.000000
C	4.300434	2.482664	0.000000
C	5.328192	3.428223	0.000000
C	6.675028	3.027643	0.000000
C	7.043931	1.690715	0.000000
C	6.030492	0.724410	0.000000
C	6.030492	-0.724410	0.000000
C	7.043931	-1.690715	0.000000
C	6.675028	-3.027643	0.000000
C	5.328192	-3.428223	0.000000
C	4.300434	-2.482664	0.000000
C	4.677724	-1.128725	0.000000
N	2.924941	2.776886	0.000000
N	2.924941	-2.776886	0.000000
C	2.324039	4.025354	0.000000
N	0.942383	3.921516	0.000000
O	2.933637	5.081208	0.000000
N	-1.934178	3.350095	0.000000
C	-3.316367	3.486665	0.000000
C	-4.300267	2.482953	0.000000
C	-5.633024	2.900238	0.000000
C	-5.959529	4.266922	0.000000
C	-4.986167	5.254866	0.000000
C	-3.642604	4.860355	0.000000
C	-2.387889	5.584765	0.000000
C	-2.057764	6.945581	0.000000
C	-0.715498	7.294565	0.000000
C	0.304832	6.328461	0.000000
C	-0.000167	4.965617	0.000000
C	-1.361357	4.615390	0.000000
N	-3.867324	1.144631	0.000000
N	-1.934178	-3.350095	0.000000
C	-1.361357	-4.615390	0.000000
C	-0.000167	-4.965617	0.000000
C	0.304832	-6.328461	0.000000
C	-0.715498	-7.294565	0.000000
C	-2.057764	-6.945581	0.000000
C	-2.387889	-5.584765	0.000000
C	-3.642604	-4.860355	0.000000
C	-4.986167	-5.254866	0.000000
C	-5.959529	-4.266922	0.000000
C	-5.633024	-2.900238	0.000000
C	-4.300267	-2.482953	0.000000
C	-3.316367	-3.486665	0.000000
N	0.942383	-3.921516	0.000000
N	-3.867324	-1.144631	0.000000
C	2.324039	-4.025354	0.000000
O	2.933637	-5.081208	0.000000
C	-4.648079	0.000000	0.000000
O	-5.867273	0.000000	0.000000
H	2.856788	0.000000	0.000000

H	5.071454	4.475857	0.000000
H	8.088404	1.400017	0.000000
H	8.088404	-1.400017	0.000000
H	5.071454	-4.475857	0.000000
H	2.298435	1.983199	0.000000
H	2.298435	-1.983199	0.000000
H	0.568283	2.982102	0.000000
H	-1.428394	2.474051	0.000000
H	-6.411933	2.154079	0.000000
H	-5.256652	6.304755	0.000000
H	-2.831752	7.704771	0.000000
H	1.340479	6.629936	0.000000
H	-2.866718	0.998904	0.000000
H	-1.428394	-2.474051	0.000000
H	1.340479	-6.629936	0.000000
H	-2.831752	-7.704771	0.000000
H	0.568283	-2.982102	0.000000
H	-2.866718	-0.998904	0.000000
H	-6.411933	-2.154079	0.000000
H	-5.256652	-6.304755	0.000000
Br	0.000000	0.000000	0.000000
H	7.438993	-3.797428	0.000000
H	-0.430827	-8.341070	0.000000
H	-7.008165	-4.543643	0.000000
H	-7.008165	4.543643	0.000000
H	-0.430827	8.341070	0.000000
H	7.438993	3.797428	0.000000

1-Cl

N	3.864207	0.000000	0.000000
C	4.674510	1.128053	0.000000
C	4.296865	2.481064	0.000000
C	5.323445	3.427727	0.000000
C	6.670396	3.027652	0.000000
C	7.040163	1.690932	0.000000
C	6.027201	0.724257	0.000000
C	6.027201	-0.724257	0.000000
C	7.040163	-1.690932	0.000000
C	6.670396	-3.027652	0.000000
C	5.323445	-3.427727	0.000000
C	4.296865	-2.481064	0.000000
C	4.674510	-1.128053	0.000000
N	2.921185	2.771789	0.000000
N	2.921185	-2.771789	0.000000
C	2.320754	4.019664	0.000000
N	0.939848	3.915715	0.000000
O	2.930392	5.075588	0.000000
N	-1.932104	3.346502	0.000000
C	-3.314177	3.484218	0.000000
C	-4.297097	2.480662	0.000000
C	-5.630221	2.896375	0.000000
C	-5.957221	4.262907	0.000000

C	-4.984472	5.251494	0.000000
C	-3.640826	4.857581	0.000000
C	-2.386376	5.581838	0.000000
C	-2.055692	6.942426	0.000000
C	-0.713175	7.290558	0.000000
C	0.306776	6.324102	0.000000
C	0.000232	4.961726	0.000000
C	-1.360333	4.612271	0.000000
N	-3.861032	1.143926	0.000000
N	-1.932104	-3.346502	0.000000
C	-1.360333	-4.612271	0.000000
C	0.000232	-4.961726	0.000000
C	0.306776	-6.324102	0.000000
C	-0.713175	-7.290558	0.000000
C	-2.055692	-6.942426	0.000000
C	-2.386376	-5.581838	0.000000
C	-3.640826	-4.857581	0.000000
C	-4.984472	-5.251494	0.000000
C	-5.957221	-4.262907	0.000000
C	-5.630221	-2.896375	0.000000
C	-4.297097	-2.480662	0.000000
C	-3.314177	-3.484218	0.000000
N	0.939848	-3.915715	0.000000
N	-3.861032	-1.143926	0.000000
C	2.320754	-4.019664	0.000000
O	2.930392	-5.075588	0.000000
C	-4.641508	0.000000	0.000000
O	-5.860784	0.000000	0.000000
H	2.852638	0.000000	0.000000
H	5.066185	4.475106	0.000000
H	8.084795	1.400890	0.000000
H	8.084795	-1.400890	0.000000
H	5.066185	-4.475106	0.000000
H	2.295372	1.977630	0.000000
H	2.295372	-1.977630	0.000000
H	0.564992	2.976665	0.000000
H	-1.426319	2.470457	0.000000
H	-6.408648	2.149891	0.000000
H	-5.255604	6.301193	0.000000
H	-2.829191	7.702083	0.000000
H	1.342463	6.624998	0.000000
H	-2.860363	0.999035	0.000000
H	-1.426319	-2.470457	0.000000
H	1.342463	-6.624998	0.000000
H	-2.829191	-7.702083	0.000000
H	0.564992	-2.976665	0.000000
H	-2.860363	-0.999035	0.000000
H	-6.408648	-2.149891	0.000000
H	-5.255604	-6.301193	0.000000
Cl	0.000000	0.000000	0.000000
H	7.434036	-3.797771	0.000000
H	-0.428052	-8.336950	0.000000
H	-7.005984	-4.539179	0.000000

H	-7.005984	4.539179	0.000000
H	-0.428052	8.336950	0.000000
H	7.434036	3.797771	0.000000

1-ClO4

N	-3.868830	0.000000	0.157545
C	-4.665532	-1.132503	-0.022853
C	-4.300649	-2.492012	0.021801
C	-5.314476	-3.431827	-0.190232
C	-6.639339	-3.028221	-0.424254
C	-7.000685	-1.686376	-0.447813
C	-6.000799	-0.725227	-0.250423
C	-6.000799	0.725227	-0.250423
C	-7.000685	1.686376	-0.447813
C	-6.639339	3.028221	-0.424254
C	-5.314476	3.431827	-0.190232
C	-4.300649	2.492012	0.021801
C	-4.665532	1.132503	-0.022853
N	-2.959891	-2.822971	0.297857
N	-2.959891	2.822971	0.297857
C	-2.299586	-3.983000	-0.086756
N	-0.964819	-3.974826	0.297857
O	-2.831723	-4.904688	-0.693395
N	1.934415	-3.350505	0.157545
C	3.313543	-3.474218	-0.022853
C	4.308470	-2.478465	0.021801
C	5.629287	-2.886557	-0.190232
C	5.942186	-4.235726	-0.424254
C	4.960787	-5.219583	-0.447813
C	3.628464	-4.834231	-0.250423
C	2.372335	-5.559458	-0.250423
C	2.039898	-6.905959	-0.447813
C	0.697153	-7.263947	-0.424254
C	-0.314812	-6.318385	-0.190232
C	-0.007822	-4.970477	0.021801
C	1.351989	-4.606721	-0.022853
N	3.924710	-1.151855	0.297857
N	1.934415	3.350505	0.157545
C	1.351989	4.606721	-0.022853
C	-0.007822	4.970477	0.021801
C	-0.314812	6.318385	-0.190232
C	0.697153	7.263947	-0.424254
C	2.039898	6.905959	-0.447813
C	2.372335	5.559458	-0.250423
C	3.628464	4.834231	-0.250423
C	4.960787	5.219583	-0.447813
C	5.942186	4.235726	-0.424254
C	5.629287	2.886557	-0.190232
C	4.308470	2.478465	0.021801
C	3.313543	3.474218	-0.022853
N	-0.964819	3.974826	0.297857

N	3.924710	1.151855	0.297857
C	-2.299586	3.983000	-0.086756
O	-2.831723	4.904688	-0.693395
C	4.599173	0.000000	-0.086756
O	5.663445	0.000000	-0.693395
H	-2.865348	0.000000	0.029788
H	-5.053865	-4.481206	-0.183678
H	-8.029900	-1.387162	-0.627257
H	-8.029900	1.387162	-0.627257
H	-5.053865	4.481206	-0.183678
H	-2.420932	-2.119761	0.789320
H	-2.420932	2.119761	0.789320
H	-0.625300	-3.156469	0.789320
H	1.432674	-2.481465	0.029788
H	6.407771	-2.136173	-0.183678
H	5.216267	-6.260516	-0.627257
H	2.813633	-7.647678	-0.627257
H	-1.353905	-6.617378	-0.183678
H	3.046233	-1.036708	0.789320
H	1.432674	2.481465	0.029788
H	-1.353905	6.617378	-0.183678
H	2.813633	7.647678	-0.627257
H	-0.625300	3.156469	0.789320
H	3.046233	1.036708	0.789320
H	6.407771	2.136173	-0.183678
H	5.216267	6.260516	-0.627257
H	6.982805	4.506624	-0.583356
H	0.411448	8.300599	-0.583356
H	-7.394253	3.793975	-0.583356
H	-7.394253	-3.793975	-0.583356
H	0.411448	-8.300599	-0.583356
H	6.982805	-4.506624	-0.583356
Cl	0.000000	0.000000	1.719917
O	1.399984	0.000000	1.173530
O	-0.699992	1.212422	1.173530
O	-0.699992	-1.212422	1.173530
O	0.000000	0.000000	3.190403

1-I

N	-0.108292	-3.866995	-0.110136
C	-1.259710	-4.646376	-0.032380
C	-2.599576	-4.218903	-0.007040
C	-3.582759	-5.212033	0.052263
C	-3.228385	-6.573042	0.061133
C	-1.904066	-6.991189	0.001436
C	-0.901186	-6.012989	-0.044280
C	0.548937	-6.059045	-0.106785
C	1.486576	-7.100299	-0.145928
C	2.835137	-6.768498	-0.198068
C	3.274925	-5.433032	-0.228846
C	2.357606	-4.377504	-0.198940
C	0.994275	-4.717783	-0.125395

N	-2.842756	-2.834085	-0.074004
N	2.688669	-3.009478	-0.240024
C	-4.048283	-2.179618	0.127475
N	-3.927503	-0.812930	-0.074807
O	-5.091043	-2.739288	0.445817
N	-3.283106	2.041399	-0.126158
C	-3.390814	3.430734	-0.147524
C	-2.358621	4.384291	-0.219581
C	-2.738335	5.730436	-0.256818
C	-4.096375	6.094436	-0.233988
C	-5.113038	5.148766	-0.182938
C	-4.756416	3.794114	-0.136329
C	-5.513674	2.557415	-0.069686
C	-6.882330	2.258309	-0.026044
C	-7.260667	0.922669	0.042242
C	-6.318553	-0.121117	0.043164
C	-4.948493	0.153610	-0.015299
C	-4.568324	1.507368	-0.048230
N	-1.032887	3.910859	-0.248429
N	3.395322	1.839543	-0.071215
C	4.646815	1.228521	-0.066753
C	4.954211	-0.145822	-0.066046
C	6.308975	-0.492753	-0.034073
C	7.306149	0.498098	0.010852
C	6.998879	1.852578	0.032358
C	5.648331	2.225063	-0.008848
C	4.962667	3.503646	-0.004427
C	5.395976	4.836020	0.040045
C	4.435547	5.839747	0.025993
C	3.058586	5.554881	-0.012315
C	2.602342	4.233142	-0.047865
C	3.579095	3.219466	-0.059277
N	3.882653	-1.058780	-0.098413
N	1.250153	3.839476	-0.072168
C	3.952131	-2.443916	-0.143125
O	4.994532	-3.087016	-0.103343
C	0.132721	4.657870	-0.149987
O	0.168777	5.882555	-0.134379
H	-0.066541	-2.903677	0.206074
H	-4.620936	-4.913641	0.090564
H	-1.650867	-8.047997	-0.004841
H	1.165321	-8.138456	-0.132760
H	4.328982	-5.200974	-0.281193
H	-2.049441	-2.261228	-0.342617
H	1.924566	-2.373411	-0.443535
H	-3.015399	-0.469795	-0.357296
H	-2.458888	1.553472	0.208861
H	-1.967613	6.486073	-0.308737
H	-6.156990	5.450718	-0.175762
H	-7.626037	3.050656	-0.039353
H	-6.639732	-1.152084	0.087647
H	-0.918527	2.924424	-0.457856
H	2.545715	1.387802	-0.392459

H	6.576235	-1.539731	-0.033423
H	7.782163	2.604692	0.077566
H	2.960244	-0.664639	0.053156
H	1.072374	2.859594	0.119778
H	2.331564	6.354163	-0.004948
H	6.455224	5.075677	0.082397
I	0.011460	-0.003977	-0.201554
H	3.582989	-7.557002	-0.226107
H	8.345378	0.180188	0.039098
H	4.745001	6.881527	0.056224
H	-4.346439	7.151879	-0.267889
H	-8.315459	0.663380	0.085606
H	-4.024645	-7.311917	0.104338

1-NO3

N	1.937636	3.356084	0.000000
C	1.359492	4.624113	0.000000
C	-0.003449	4.990578	0.000000
C	-0.293083	6.356738	0.000000
C	0.733831	7.314970	0.000000
C	2.072409	6.953270	0.000000
C	2.390401	5.589390	0.000000
C	3.645353	4.864843	0.000000
C	4.985504	5.271394	0.000000
C	5.968034	4.293001	0.000000
C	5.651638	2.924552	0.000000
C	4.323692	2.492302	0.000000
C	3.324853	3.489411	0.000000
N	-0.966228	3.966185	0.000000
N	3.917931	1.146314	0.000000
C	-2.351750	4.073350	0.000000
N	-2.951702	2.819871	0.000000
O	-2.961650	5.129729	0.000000
N	-3.875273	0.000000	0.000000
C	-4.684345	-1.134702	0.000000
C	-4.320242	-2.498276	0.000000
C	-5.358555	-3.432186	0.000000
C	-6.701865	-3.021968	0.000000
C	-7.057913	-1.681876	0.000000
C	-6.035755	-0.724547	0.000000
C	-6.035755	0.724547	0.000000
C	-7.057913	1.681876	0.000000
C	-6.701865	3.021968	0.000000
C	-5.358555	3.432186	0.000000
C	-4.320242	2.498276	0.000000
C	-4.684345	1.134702	0.000000
N	-2.951702	-2.819871	0.000000
N	1.937636	-3.356084	0.000000
C	3.324853	-3.489411	0.000000
C	4.323692	-2.492302	0.000000
C	5.651638	-2.924552	0.000000
C	5.968034	-4.293001	0.000000

C	4.985504	-5.271394	0.000000
C	3.645353	-4.864843	0.000000
C	2.390401	-5.589390	0.000000
C	2.072409	-6.953270	0.000000
C	0.733831	-7.314970	0.000000
C	-0.293083	-6.356738	0.000000
C	-0.003449	-4.990578	0.000000
C	1.359492	-4.624113	0.000000
N	3.917931	-1.146314	0.000000
N	-0.966228	-3.966185	0.000000
C	4.703499	0.000000	0.000000
O	5.923300	0.000000	0.000000
C	-2.351750	-4.073350	0.000000
O	-2.961650	-5.129729	0.000000
H	1.433187	2.482352	0.000000
H	-1.327439	6.664574	0.000000
H	2.853855	7.704787	0.000000
H	6.435410	2.182691	0.000000
H	-0.599985	3.025473	0.000000
H	2.920129	0.993135	0.000000
H	-2.320144	2.032338	0.000000
H	-2.866374	0.000000	0.000000
H	-5.107971	-4.481883	0.000000
H	-8.099469	-1.380883	0.000000
H	-8.099469	1.380883	0.000000
H	-5.107971	4.481883	0.000000
H	-2.320144	-2.032338	0.000000
H	1.433187	-2.482352	0.000000
H	6.435410	-2.182691	0.000000
H	5.245614	-6.323904	0.000000
H	2.853855	-7.704787	0.000000
H	2.920129	-0.993135	0.000000
H	-0.599985	-3.025473	0.000000
N	0.000000	0.000000	0.000000
O	1.249585	0.000000	0.000000
O	-0.624792	1.082172	0.000000
H	-1.327439	-6.664574	0.000000
H	5.245614	6.323904	0.000000
O	-0.624792	-1.082172	0.000000
H	-7.472732	3.784940	0.000000
H	0.458512	8.364045	0.000000
H	7.014220	4.579106	0.000000
H	0.458512	-8.364045	0.000000
H	7.014220	-4.579106	0.000000
H	-7.472732	-3.784940	0.000000
